# Use of Electron-Transfer Mediators to Access Higher Electrochemical Current Densities in Ni-Catalyzed Cross-Electrophile Coupling

**DOI:** 10.1021/jacs.5c10599

**Published:** 2025-09-25

**Authors:** Jieru Zhu, Shannon S. Stahl

**Affiliations:** † Department of Chemistry, 5228 University of Wisconsin−Madison, Madison, Wisconsin 53706, United States

## Abstract

Ni-catalyzed cross-electrophile coupling (XEC) reactions are versatile methods for carbon–carbon bond formation, and they often use electrochemistry to supply the requisite electrons. These reactions, however, typically exhibit slow rates and operate at low current densities (e.g., 1–4 mA/cm^2^) to avoid formation of undesirable side products. Here, we show that much higher current densities and reaction selectivity can be accessed by using a homogeneous electron-transfer (ET) mediator. Bis­(ethylcyclopentadienyl)­cobalt­(II), which has a redox potential similar to that of the bipyridyl-ligated Ni catalyst (−1.45 and –1.50 V vs Fc/Fc^+^, respectively), exhibits the best performance. This ET-mediator strategy is applied to numerous C­(sp^2^)–C­(sp^3^) coupling reactions and extended to a flow-based reaction on 10 mmol-scale that proceeds in 96% yield with 91% Faradaic efficiency at a current density of 18 mA/cm^2^.

## Graphical abstract

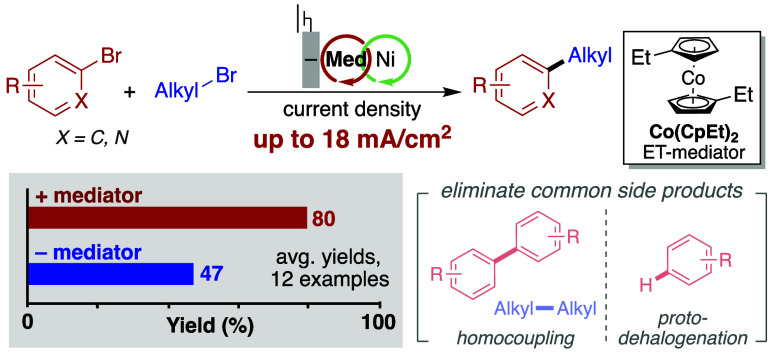

Nickel-catalyzed cross-electrophile coupling (XEC) reactions are the focus of active research effort and application.^1,2^ Electrochemical XEC methods (eXEC) are being widely developed,^3,4^ and they offer an appealing approach for larger scale applications.^5–17^ Such methods would retain the ability to use the more-abundant, lower-cost building blocks associated with XEC, while avoiding complications associated with large-scale use of metal-powder reductants.^18,19^ A survey of electrochemical Ni-catalyzed XEC reactions, however, highlights a major complication. The majority of these applications operate at current densities of ≤4 mA/cm^2^ (Figure 1A).^4,13,14,16,20–29^ The low productivity associated with these current densities (≤0.75 mol/h·m^2^) will limit larger scale applications by requiring untenably long production runs or larger reactors that incur high capital cost. This performance contrasts other mediated electrochemical reactions, such as nitroxyl-catalyzed alcohol oxidation, which can access current densities of ≥300 mA/cm^2^ (≥56 mol/h·m^2^).^30,31^ Attempts to operate eXEC reactions at higher current densities, however, leads to catalyst decomposition and/or lower reaction selectivity.^13,15,32,33^ A representative example shown in Figure 1B^11^ shows how excellent product yield and selectivity at current densities of ≤4 mA/cm^2^ is severely compromised at higher current densities, leading to lower yields and significant side product formation (biaryl, protodehalogenated arene). This limitation can be partially addressed by using higher catalyst loading to offset the slow catalytic turnover rates. Ni-eXEC reactions that operate above 2 mA/cm^2^ often feature Ni catalyst loadings of 10–30 mol %.^20,33–36^ Here, we explore an alternative strategy to address the current density limitations of Ni-eXEC reactions, using an electron-transfer (ET) mediator to enable catalytic turnover, even when the catalysts is not in proximity to the electrode (Figure 1C).^37–41^ This strategy, which differs from the ″overcharge protection″ strategy introduced by Sevov and co-workers,^9^ leads to significant improvement in reaction performance, supporting higher current densities at high faradaic efficiency and excellent product selectivity.

**Figure 1:**
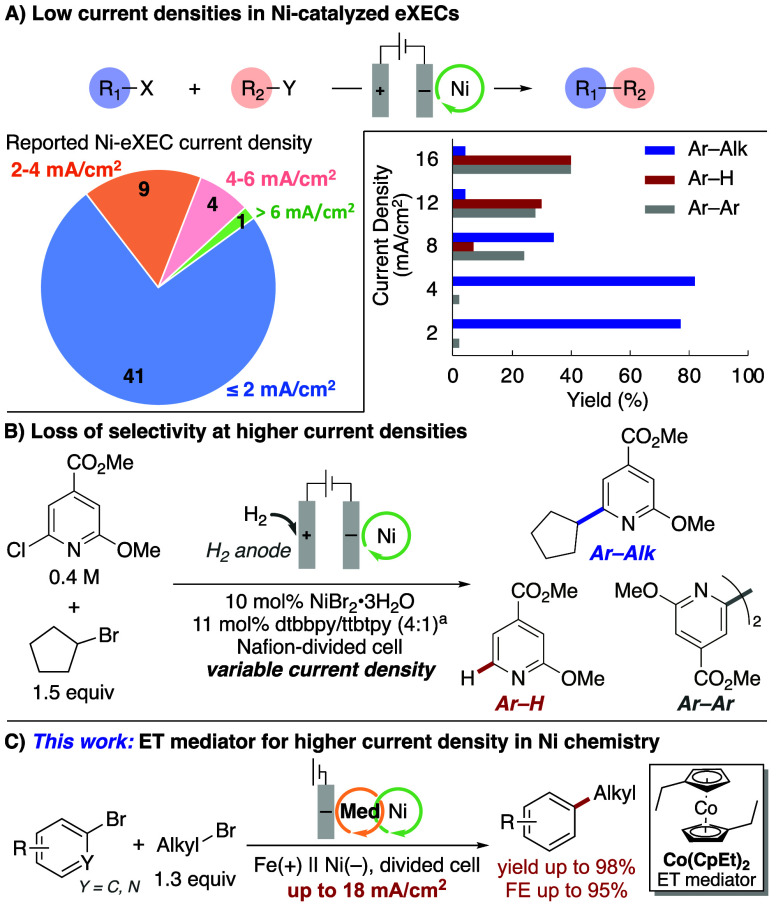
(A) Low current density is a general limitation in Ni-eXEC reactions. (B) Example of selectivity degradation in Ni-eXEC at higher current densities. (C) Present effort using an ET mediator to support higher current density. ^a^dtbbpy = 4,4′-di*tert*-butyl-2,2′-bipyridine, ttbtpy = 4,4′,4″-tri*tert*-butyl-2,2′:6′,2″-terpyridine.

Ni-catalyzed eXEC of ethyl 4-bromobenzoate (**1a**) and 1-bromo-3-phenylpropane (**2a**) was selected for our initial studies. Initial reactions were carried out using a 1 cm^2^ Ni foam cathode as the working electrode and an Fe rod sacrificial anode in a divided H-cell equipped with a Nafion 115 membrane. Electrolysis was performed at constant current (8 mA; 4 mA/cm^2^).^42,43^ Two metrics were assessed in the initial screening efforts, the yield of cross-coupled product **3a** and the cross-selectivity, defined as the ratio of the yields of **3a** and the side products, **3a**/(**1a–H** + **1aa**). Tetrakis­(dimethylamino)­ethylene (TDAE), an effective homogeneous reductant in Ni XEC reactions,^44^ was selected as the ET mediator to evaluate with established Ni catalyst systems. A survey of different conditions showed optimal results with 1 mol % NiBr_2_/dtbbpy (dtbbpy = 4,4′-di*tert*-butyl-2,2′-bipyridine) paired with cocatalytic CoPc (2.5 mol %, Pc = phthalocyanine)^45,46^ (see Section 3.1 of the Supporting Information for screening and optimization data). At this low catalyst loading, the inclusion of 10 mol % TDAE led to significantly improved performance relative to a reaction lacking TDAE (Figure 2A): **3a** was obtained in 78% yield and with a cross-selectivity of 3.7, while these metrics dropped to 45% yield and 0.8 cross-selectivity in the absence of TDAE. These promising initial results prompted us to evaluate other homogeneous ET mediators, focusing on tetrakis­(amino)­ethylenes^47^ and cobaltocenes, which have redox potentials above and below the potential of the Ni/dtbbpy catalyst (−1.50 V vs Fc/Fc^+^; all potentials are referenced to Fc/Fc^+^) (Figure 2B).

**Figure 2:**
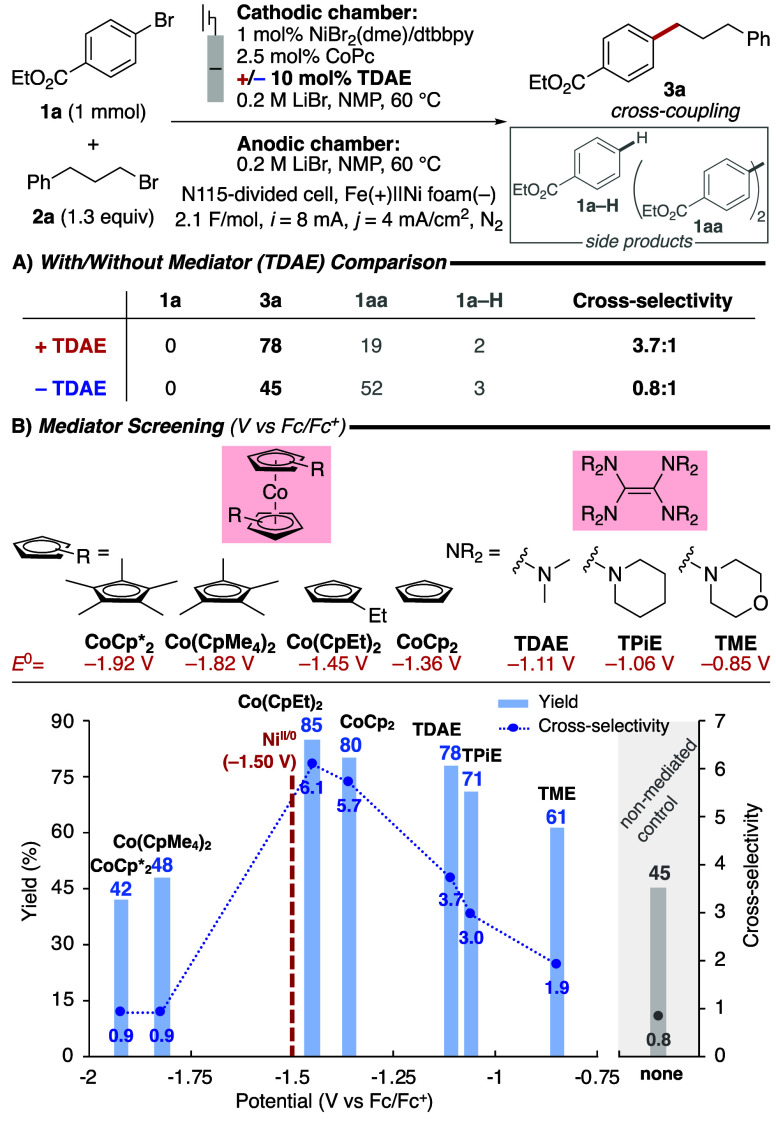
(A) Screening of the optimized reaction condition with (red) or without (blue) TDAE as an ET mediator. (B) ET mediator screening. Yields determined by ^1^H NMR spectroscopy (int. std. = 1,3,5-trimethoxybenzene). Cross-selectivity = **3a** yield/(**1a**–**H** yield + **1aa** yield), with yields of **1aa** determined with respect to the stoichiometry of the substrate, i.e., the maximal theoretical **1aa** yield is 100%.

The data obtained with mediators are depicted in Figure 2B (yields as blue bars, selectivity as blue dots) alongside the results obtained in the absence of a mediator (gray bar/dot). The least reducing mediator, tetrakis­(morpholino)­ethylene (TME), yielded 61% **3a** with 1.9 cross-selectivity. These metrics steadily improved as the mediator potential approached the value of the Ni/dtbbpy catalyst (red), with optimal results observed with 1,1′-diethylcobaltocene (Co­(CpEt)_2_): 85% yield of **3a** with 6.1 cross-selectivity. Use of mediators with a potential below that of the Ni/dtbbpy catalyst had a deleterious effect, resulting in reaction outcomes comparable to those under nonmediated conditions (Figure 2B). CV analysis confirmed that cobaltocene ET mediators do not react directly with substrates (see Figure S12 and S15 in the Supporting Information), contrasting the role of CoPc, which activates the alkyl halide electrophile.^45,46^ A full catalytic mechanism highlighting these different roles of Co­(CpEt)_2_ and CoPc is provided in Figure S10 of the Supporting Information.

These results indicate that the optimal ET mediator undergoes slightly endergonic ET to the Ni catalyst, aligning with other mediated reactions.^37,38,41^ This feature differs from the “overcharge protection” strategy,^9^ which uses a redox-active species with a potential below that of the Ni catalyst. The latter approach supports improved reaction selectivity by maintaining a steady cell potential via nonproductive redox cycling of the mediator in an undivided cell. The overcharge protection method was tested in the present reaction, but it is effective only at current densities ≤4 mA/cm^2^. The significantly lower yields and Faradaic efficiencies observed above 4 mA/cm^2^ illustrates the conceptual and practical differences between these two mediated methods for Ni-eXEC (see Section 3.2 of the Supporting Information for data and additional discussion).

To assess the generality of ET-mediated Ni-eXEC, we examined the cross-coupling of a series of aryl and alkyl bromides featuring different functional groups and steric properties (Figure 3A). For each product, we show three sets of yield and FE results obtained from eXEC using the optimized Co­(CpEt)_2_-mediated condition (red), from eXEC using the optimized condition without the mediator (blue), and literature data, if available (gray). Cross-coupling reactions of the model alkyl substrate **2a** with aryl bromides containing either an electron-withdrawing substituent (**3a**,^48^ **3b** ^46^) or an electron-donating substituent (**3c** ^46^) at the para position were all effective and gave comparable or better yields than the literature precedents. More hindered ortho- and meta-substituted aryl substrates (**3d**,^49^ **3e** ^50^) were tolerated and gave substantially better yields than previously reported. Structurally complex dihydronaphthalenone, indole, tetrahydroquinoline, and pyridine substrates were also successful, providing access to novel products (**3f**, **3g**, **3h**, **3i**). Varying the alkyl coupling partner showed that the synthesis was effective with a different primary alkyl bromide (**3j** ^50^). Coupling of **1a** with secondary (**3k** ^7^) and benzylic (**3l**) alkyl bromides was achieved with slightly modified conditions and gave good yields. In all cases, the mediated conditions outperformed the nonmediated conditions, exhibiting higher yields and Faradaic efficiencies; however, the beneficial effect can vary with different substrates. Less reactive aryl electrophiles (electron-rich, sterically hindered) appear to benefit less than more reactive aryl electrophiles. For example, the formation of **3c** and **3e**, derived from electron-rich and ortho-substituted aryl substrates, respectively, show comparatively modest influence of the ET mediator (12–15% yield improvement). These observations suggest that mass transport between bulk solution and the electrode can adequately support reactions that undergo slower catalytic turnover (i.e., with kinetically challenging substrates) and thereby lead to less benefit from the ET mediator. The mediator benefit is not limited to the present catalyst system, as revealed by tests of previously reported Ni-catalyzed (e)­XEC conditions with different catalyst systems and/or electrophile pairs.^7,13,44^ Even without reoptimization of the conditions, the inclusion of 10 mol % Co­(CpEt)_2_ to the reported conditions led to modest-to-major improvement in the product yields (Figure 3B, see section 4 of the Supporting Information for additional examples and experimental description).

**Figure 3:**
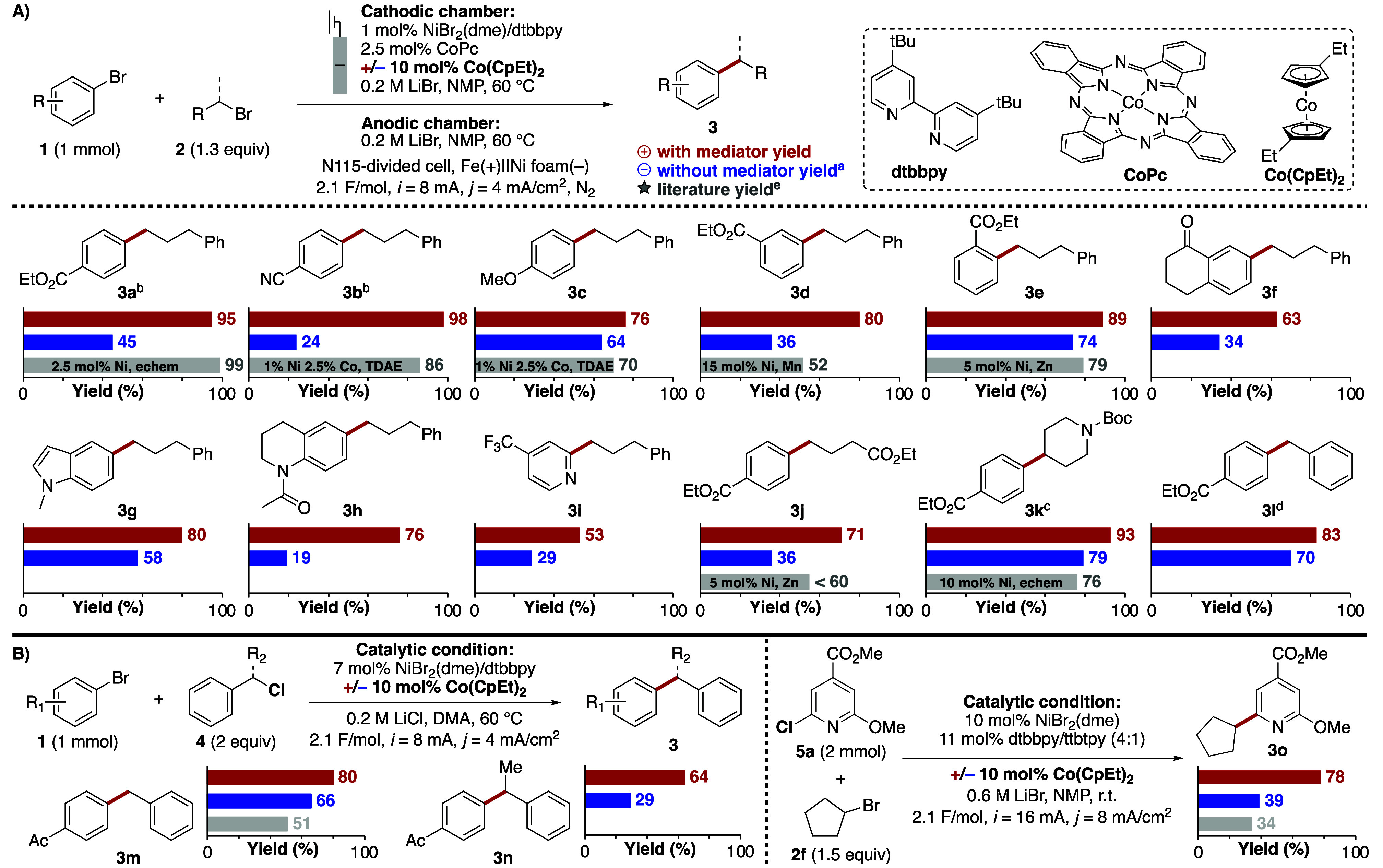
Substrate scope for eXEC of (A) aryl bromides with alkyl bromides under optimized conditions and (B) other literature methods with different electrophile pairs. For each product, three sets of yields are provided. Red: isolated yields from the optimized mediated condition. Blue: NMR yields from the optimized catalytic system without mediator. Gray: literature-reported results, where applicable. ^a^NMR spectroscopy yield (int. std. = 1,3,5-trimethoxybenzene). ^b^2 mol % NiBr_2_(dme)/dtbbpy, 5 mol % CoPc. ^c^5 mol % NiBr_2_(dme), 5 mol % dtbbpy/ttbtpy (4:1), no CoPc. ^d^5 mol % NiBr_2_(dme)/dtbbpy, no CoPc, DMA as reaction solvent. ^e^Literature refs: **3a**;^48^ **3b**;^46^ **3c**;^46^ **3d**;^49^ **3e**;^50^ **3j**;^50^ **3k**;^7^ **3m**;^44^ **3o**.^13^

To investigate the role of the mediator further, we monitored the time course of the reaction of **1a** and **2a** using a three-electrode configuration to probe differences in the cathode potential in the presence and absence of the Co­(CpEt)_2_ mediator (Figure 4). The two conditions, probed at a common current density of 6 mA/cm^2^, gave >90% yield with mediator and <40% yield without mediator (Table S7). The cathode potential was monitored throughout the reaction in both cases (Figure 4A), and the substrates and products were monitored by withdrawing aliquots from the reaction mixture every 30 min and analyzing them by ^1^H NMR spectroscopy (Figures 4B and 4C). The reaction containing the mediator maintained a steady working potential of approximately –1.5 V over the first 3 h of the reaction, during which only cross-coupled product **3a** was formed as a product (Figures 4A and 4B). A drop in applied potential beyond this time correlated with observation of small amounts of side product **1aa**. The latter observation is rationalized by reductively induced aryl transmetalation and biaryl formation at lower applied potentials.^51^ In spite of this dropoff, the mediated reaction generated the desired product **3a** in 91% yield after passing 2.1 F/mol of charge. The reaction performed in the absence of the mediator started at a lower potential of –2.3 V and dropped sharply to –4.2 V after 30 min, possibly reflecting partial fouling of the electrode, and then lowered further after 2 h through the end of the reaction (Figures 4A and 4C). This lower operating potential correlates with lower product yield and lower selectivity, with formation of side products **1aa** and **1a–H** starting at the beginning of the reaction. The reaction did not reach completion after passing 2.1 F/mol of charge, and formed **3a** in only 32% yield, together with 16% **1aa** and 12% **1a–H**. These observations indicate that the mediator supports a stable working potential at this elevated current density, limiting catalyst decomposition and side product formation.

**Figure 4:**
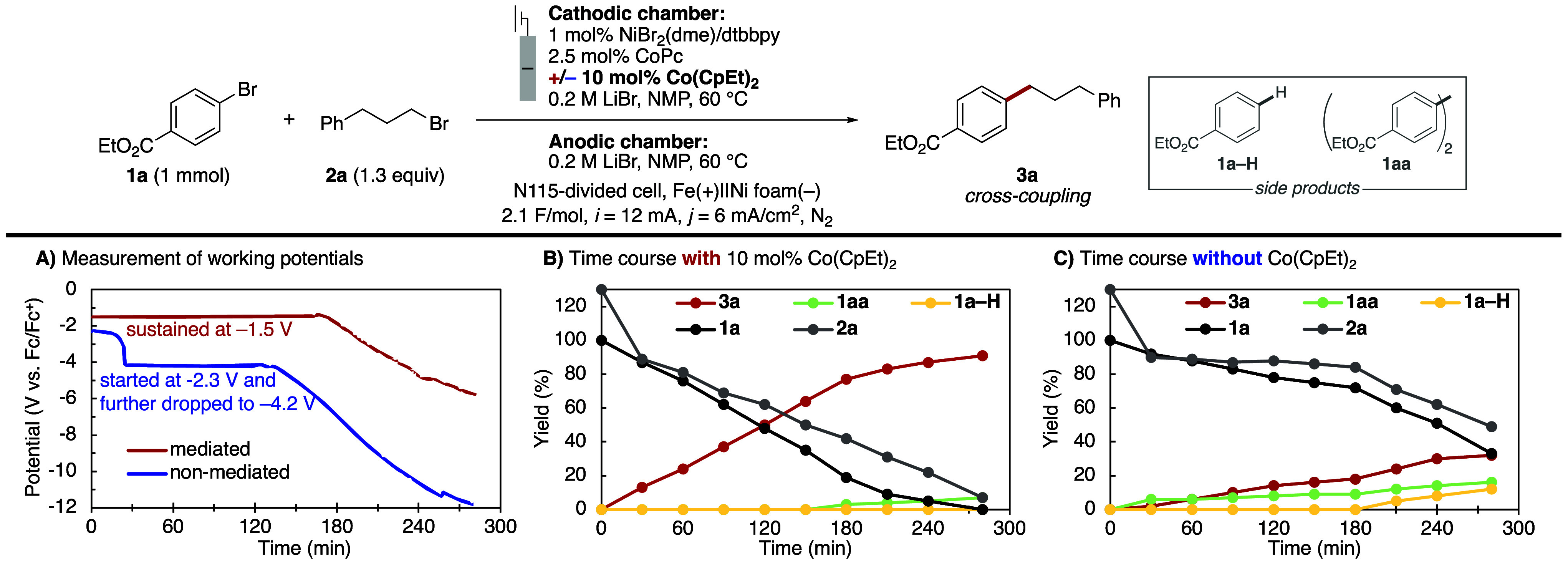
Reaction progress of the optimized condition with a three-electrode setup at 6 mA/cm^2^ current density. (A) Monitored working potential throughout the reaction with (red) or without (blue) Co­(CpEt)_2_. (B) Time course with Co­(CpEt)_2_. (C) Time course without Co­(CpEt)_2_. Yields determined by ^1^H NMR spectroscopy (int. std. = 1,3,5-trimethoxybenzene), with yields of **1aa** determined with respect to the stoichiometry of the substrate; i.e., the maximal theoretical **1aa** yield is 100%.

The benefits of this ET-mediator strategy will be especially beneficial at larger scales, where the increased productivity and shorter reaction times with higher current densities are more impactful. In the same batch reaction configuration, the current density could be increased to 12 mA/cm^2^ while forming product **3a** in 95% yield by doubling the catalyst loading (Figure 5, left) (2 mol % Ni/dtbbpy, 5 mol % CoPc). This effect was amplified by using a parallel-plate flow reactor, which enabled even higher throughput. After slight adjustment of the substrate, electrolyte, concentrations and catalyst loading, the reaction was performed on 10 mmol scale with a current density of 18 mA/cm^3^. The reaction reached completion within 3.5 h and generated the desired cross-coupled product in 96% yield upon passing 2.1 F/mol of charge (91% Faradaic efficiency) (Figure 5, right). To our knowledge, this current density is the highest yet reported for Ni-catalyzed eXEC. This metric translates to a throughput of 820 g/h·m^2^ or 6.6 kg/day·m^2^ (for an 8 h day). In the absence of the ET mediator, the current density in this flow electrolysis configuration was limited to 3 mA/cm^2^ and formed the product in only 73% yield (Figure 5, right, gray numbers).

**Figure 5:**
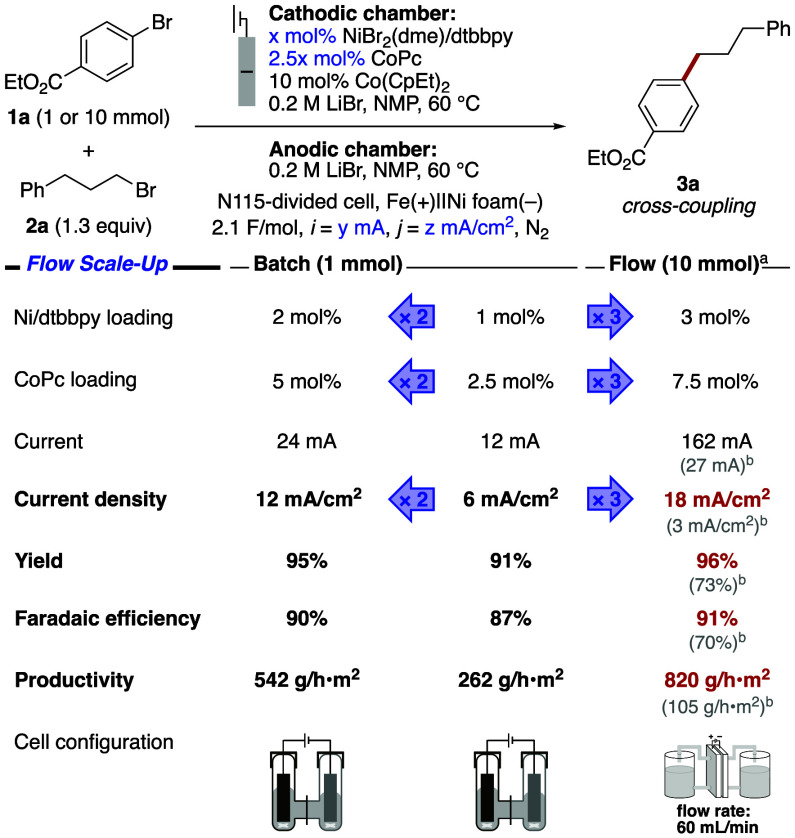
Current density optimization in batch (left) and large-scale flow (right) electrolysis. The reaction outcomes of nonmediated flow electrolysis are presented (gray) below the mediated results (red). ^a^0.5 M **1a** and 0.65 M **2a** in the cathodic reservoir, and 0.4 M LiBr in both reservoirs. ^b^No Co­(CpEt)_2_.

The results outlined above highlight the ability of an ET mediator to support significantly improved performance in Ni-catalyzed eXEC reactions. The higher accessible current densities provide access to much higher reaction productivity, while also enabling higher selectivity for the desired product formation. The ET mediator achieves these benefits by serving as a “soluble electrode”, capable of supplying electrons to the XEC (co)­catalysts, even when they are not in proximity to the electrode surface. Concepts of this type could play a crucial role in supporting practical, large-scale applications of Ni XEC reactions.

## Supplementary Material



## Notes

Additional tables of optimization data and screenings, detailed experimental procedures, CV studies, characterization of products, and copies of NMR spectra (PDF)
