# Supplementary material for: Use of Electron-Transfer Mediators to Access Higher Electrochemical Current Densities in Ni-Catalyzed Cross-Electrophile Coupling
Source: J Am Chem Soc. 2025 Sep 25;147(40):36053–8. doi: 10.1021/jacs.5c10599 (PMC12512185; doi:10.1021/jacs.5c10599)

Supporting Information

Use of Electron-Transfer Mediators to Access Higher Electrochemical Current Densities in Ni-Catalyzed Cross-Electrophile Coupling

Jieru Zhu[1], Shannon S. Stahl[1*]

[1] Department of Chemistry, University of Wisconsin-Madison, Madison, Wisconsin 53706, United States

stahl@chem.wisc.edu

Table of Contents

1. General Experimental Considerations S2
2. General Procedures for Scope Investigation and Flow Electrolysis S3
3. Reaction Condition Optimization and Additional Screenings S7
4. Improving Literature-Reported Methods with ET-Mediator S18
5. Time Course Analysis of Mediated- and Non-Mediated-Reactions and Mechanistic Cycle . S21
6. Cyclic Voltammetry (CV) Studies S23
7. Electrochemically Active Surface Area (ECSA) Measurement of Ni Foam S28
8. Compound Characterization Data S30
9. References S36
10. NMR Spectra of Compounds S37

1. General Experimental Considerations

Solvents and reagents

All reagents were purchased from commercial sources and used as received without further purification. Starting materials (aryl and alkyl halides) were purchased from commercial sources (MilliporeSigma, Alfa Aesar, Thermo Scientific, TCI, Ambeed, Combi-Blocks). Nickel(II) bromide ethylene glycol dimethyl ether complex ($NiBr_2$(dme)) was purchased from MilliporeSigma. Ligands were purchased from MilliporeSigma. Cobalt phthalocyanine was purchased from MilliporeSigma. Electron-transfer mediators were purchased from MilliporeSigma or supplied by the Hazari group at Yale University. Anhydrous electrolytes were purchased from MilliporeSigma. All metal reagents and electrolytes were stored and handled in a nitrogen-filled glove box and used as-is. Anhydrous solvents were purchased from MilliporeSigma and stored over activated 4 Å molecular sieves in a nitrogen-filled glove box.

Electrodes and membranes

All electrode materials were purchased from commercial sources and used as received. Ni foams (1.6 mm thickness) were purchased from MTI Corporation and cut to size. Fe rods (5 mm diameter) were purchased from American-Scientific. Other metal electrodes (Zn, Mg, Al) were purchased from MilliporeSigma. Glassy carbon working electrodes (MF-2012) and non-aqueous reference electrodes (MF-2062) were purchased from BASi. Pt wires were purchased from MilliporeSigma and custom-made as Pt counter electrodes. Nafion 115 membrane was purchased from Ion Power Inc. as a 30 cm × 30 cm sheet and cut to size.

Characterization of products

All proton (^1H), carbon (^{13}C), and fluorine (^{19}F) nuclear magnetic resonance (NMR) spectra were recorded on a Bruker Avance 400 MHz spectrometer at 25 °C (^1H 400.1 MHz, ^{13}C 100.6 MHz, ^{19}F 376.5 MHz) or a Bruker Avance 500 MHz spectrometer at 25 °C (^1H 500.1 MHz, ^{13}C 125.7 MHz, ^{19}F 470.6 MHz), using $CDCl_3$ (99.8 atom% D, contains 0.03–1% (v/v) TMS) as the solvent. Chemical shifts are given in parts per million (ppm) relative to residual solvent peaks in the ^1H and ^{13}C NMR spectra or are referenced as noted. An absolute referencing method was used for ^{19}F NMR chemical shifts, based on the frequency of solvent peaks in the ^1H NMR spectra. The following abbreviations (and their combinations) are used to label the multiplicities: s (singlet), d (doublet), t (triplet), q (quartet), p (pentet), and m (multiplet). High-resolution mass spectra were obtained using a Thermo Q ExactiveTM Plus in the mass spectrometry facility at the University of Wisconsin (data were collected by facility staff). Automatic normal phase column chromatography was performed using reusable Silicycle Silia*Sep* premium cartridges (40 g, 25 µm) on a Biotage® Selekt or a Biotage® Isolera One.

Electrochemical experiments

All cyclic voltammetric (CV) and chronoamperometric (CA) experiments were performed using a Pine WaveNow PGstat (for reactions in undivided cells) or a Dr. Meter PS-305DM 30V/5A DC power supply (for reactions in divided cells). The three-electrode setup for monitoring the working potential in a divided cell used a Bio-Logic SAS BP-300 bipotentiostat. The actual current applied by each of the three Dr. Meter power supplies used for electrolysis screening was slightly different from the current displayed on the power supply. For 8 mA applied current, the real currents measured with a Radio Shack digital multimeter were between 9.8 mA to 11.7 mA. For the current density screening in Section 3.2, applied currents of 4 mA were measured to actually be 6.2 mA

to 7.3 mA; applied currents of 12 mA were measured to actually be 13.6 mA to 14.1 mA; applied currents of 14 mA were measured to actually be 14.6 mA to 15.2 mA; applied currents of 16 mA were measured to actually be 16.6 mA to 17.5 mA. For the flow electrolysis, the applied current of 162 mA was measured to actually be 163.2 mA. The CV experiments were carried out in a three-electrode cell configuration with a glassy carbon (GC) working electrode (3 mm diameter), and a platinum wire counter electrode (~1.0 cm, spiral wire). The working electrode potentials were measured versus a $Ag/AgNO_3$ reference electrode (internal solution of 0.1 M $TBAPF_6$ and 0.01 M $AgNO_3$ in DMF). The redox potential of ferrocene/ferrocenium (Fc/Fc^+) was measured (under the same experimental conditions) and used to provide an internal reference. The potential values were then adjusted relative to Fc/Fc^+, and electrochemical studies in organic solvents were reported accordingly. The GC working electrode was polished with alumina powder (5 µm) before each experiment. All solutions used for CV analysis were prepared 3 h before the experiments and kept under nitrogen atmosphere using a thin Teflon tube to allow continuous nitrogen bubbling. Bulk electrolysis experiments were performed in custom-built divided cells, with Ni foam working electrodes and Fe counter electrodes. Divided H–cells were constructed in the University of Wisconsin-Madison Chemistry Department glass-blowing facility from two Ace glass 7646-06 O-ring seal joints (9 mm) and two 14/20 joints. Gram scale flow experiments were conducted in an in-house built parallel plate reactor (see **GP 2**). Masterflex L/S Cole-Palmer peristaltic pumps with size 16 Masterflex PharMed BPT tubing were used to recirculate the anolyte and catholyte through the parallel plate.

2. General Procedures for Scope Investigation and Flow Electrolysis

*General procedure for XEC batch electrolysis (**GP 1**)*

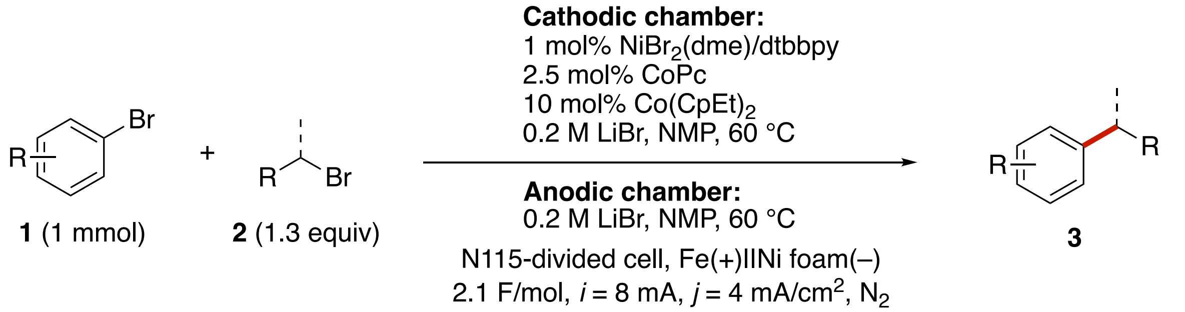

Reaction set-up (Figure S1): In a nitrogen-filled glovebox, $NiBr_2$(dme) (3.1 mg, 1 mol%), 4,4′-di-*tert*-butyl-2,2′-bipyridine (2.7 mg, 1 mol%), and anhydrous NMP (5 mL) were added to a 2-dram vial equipped with a PTFE-coated stir bar. This solution was stirred at 800 rpm in the glovebox for 1 h to allow complexation of Ni with the ligand.

To the cathodic chamber of the divided cell fitted with a cross-shaped stir bar was added aryl bromide substrate (1 mmol, 1.0 equiv), alkyl bromide substrate (1.3 mmol, 1.3 equiv), cobalt phthalocyanine (14.3 mg, 2.5 mol%), bis(ethylcyclopentadienyl)cobalt(II) (24.5 mg, 16.2 µL, 10 mol%), LiBr (87 mg, 1 mmol, 0.2 M), and the pre-stirred catalyst solution (5 mL). Then to the anodic chamber of the divided cell fitted with a cross-shaped stir bar was added LiBr (139.2 mg, 1.6 mmol, 0.2 M) and anhydrous NMP (8 mL). The cathodic chamber was then equipped with a 1.0 cm × 1.0 cm Ni foam cathode, and the anodic chamber was equipped with a Fe rod anode. The two chambers were sealed with rubber septa and removed from the glovebox. To each chamber was introduced a thin Teflon tube to allow continuous nitrogen bubbling. The reaction mixture

was stirred at 1100 rpm for 30 min in a sand bath heated to 60 °C to allow full dissolution of LiBr and exclusion of adventitious oxygen. After that, the reaction mixture was electrolyzed under constant current electrolysis at 8 mA for 7 h (2.1 F/mol) at 60 °C.

GP 1-2: For *reactions with a 2° alkyl bromide* (**3k**), $NiBr_2$(dme) (15.5 mg, 5 mol%), 4,4′-di-*tert*-butyl-2,2′-bipyridine (10.7 mg, 4 mol%) and 4,4′,4″-tri-tert-butyl-2,2′:6′,2″-terpyridine (4.0 mg, 1 mol%) were pre-stirred and used as the catalyst. Cobalt phthalocyanine was excluded from the reaction condition.

GP 1-3: For *reactions with a benzylic bromide* (**3l**), $NiBr_2$(dme) (15.5 mg, 5 mol%) and 4,4′-di-*tert*-butyl-2,2′-bipyridine (13.4 mg, 5 mol%) were pre-stirred and used as the catalyst. Cobalt phthalocyanine was excluded from the reaction condition, and DMA was used as the solvent for the reaction.

GP 1-4: For *controlled reactions without mediator* (results shown as blue bars in Figure 5 in the manuscript), **GP 1, GP 1-2,** or **GP 1-3** was followed with the exclusion of bis(ethylcyclopentadienyl)cobalt(II) from the reaction condition.

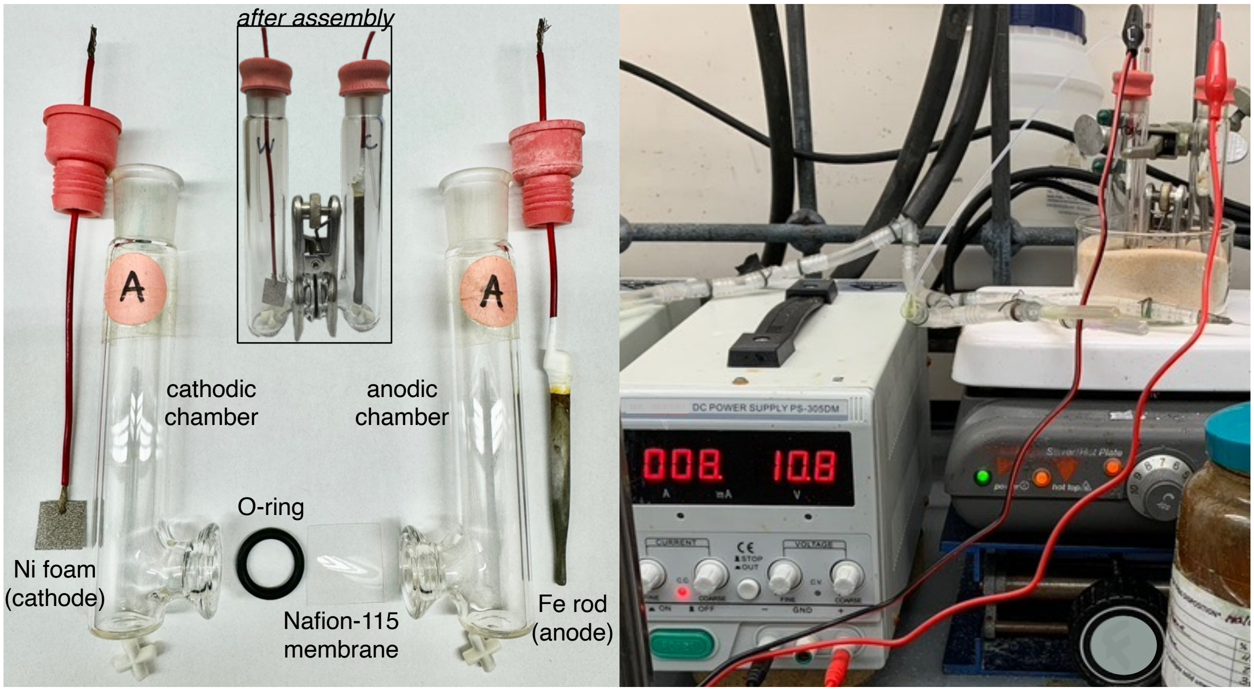

Figure S1. Graphic illustration of the divided cell before and after the assembly (left) and the set up used during the electrolysis (right).

Work-up for NMR analysis: When the electrolysis was done, the resultant reaction mixture was cooled to room temperature, and 1,3,5-trimethoxybenzene (56 mg, 0.33 equiv) was added to the reaction mixture and stirred until fully dissolved. A 100 µL aliquot of the solution was diluted with 500 µL $CDCl_3$ and filtered through a short plug of silica (2.5 cm thick × 7 mm diameter) into a test tube, and the silica plug was washed with additional 1 mL $CDCl_3$. Then 500 µL of the resultant solution was added to an NMR sample tube, and the sample was analyzed by ^1H NMR spectroscopy and yields were determined using 1,3,5-trimethoxybenzene as the internal standard.

Work-up for product isolation: The reaction mixture was quenched with 100 mL H_2O, followed by extraction with 150 mL of ethyl acetate (EtOAc). The organic layer was then washed with H_2O (80 mL × 2) and brine (80 mL). The aqueous layers were combined and back extracted with 100 mL EtOAc. The organic layers were combined and dried over anhydrous Na_2SO_4 and concentrated *in vacuo*. The obtained residue was purified by flash column chromatography with hexane/EtOAc.

*General procedure for XEC flow electrolysis (**GP 2**)*

For the electrochemical flow reactions, an in-house built parallel plate reactor with an electrode area of 9 cm² was used. The active reactor volume is 5 mL, and a Pine WaveNow PGstat was used as the power supply. The divided flow cell consists of PTFE end frames, a Fe plate (99.9%) as the anode, and a stainless steel plate and a graphite plate overlaying together as the cathodic electron collector. The flow cell also contains the flow frames and gaskets. Nickel foam with a dimension of 3.0 cm × 3.0 cm was used as the cathode. A Nafion-115 sheet of the same dimensions of the gaskets was added halfway between the cathode and the anode, taking care to create two identical chambers on both sides (same inner volume for cathode and anode to avoid differences in pressure). Additional PTFE meshes were used in both chambers as turbulence materials for diffusion. All flow electrolysis reactions were performed in NMP solutions. A magnetic stir bar was used in each reservoir which were stirred (600 rpm) during flow electrolysis reactions. The solutions were pumped through the system via a peristaltic pump with a flow rate of 60 mL/min. The components of the electrochemical cell are shown in Figure S2.

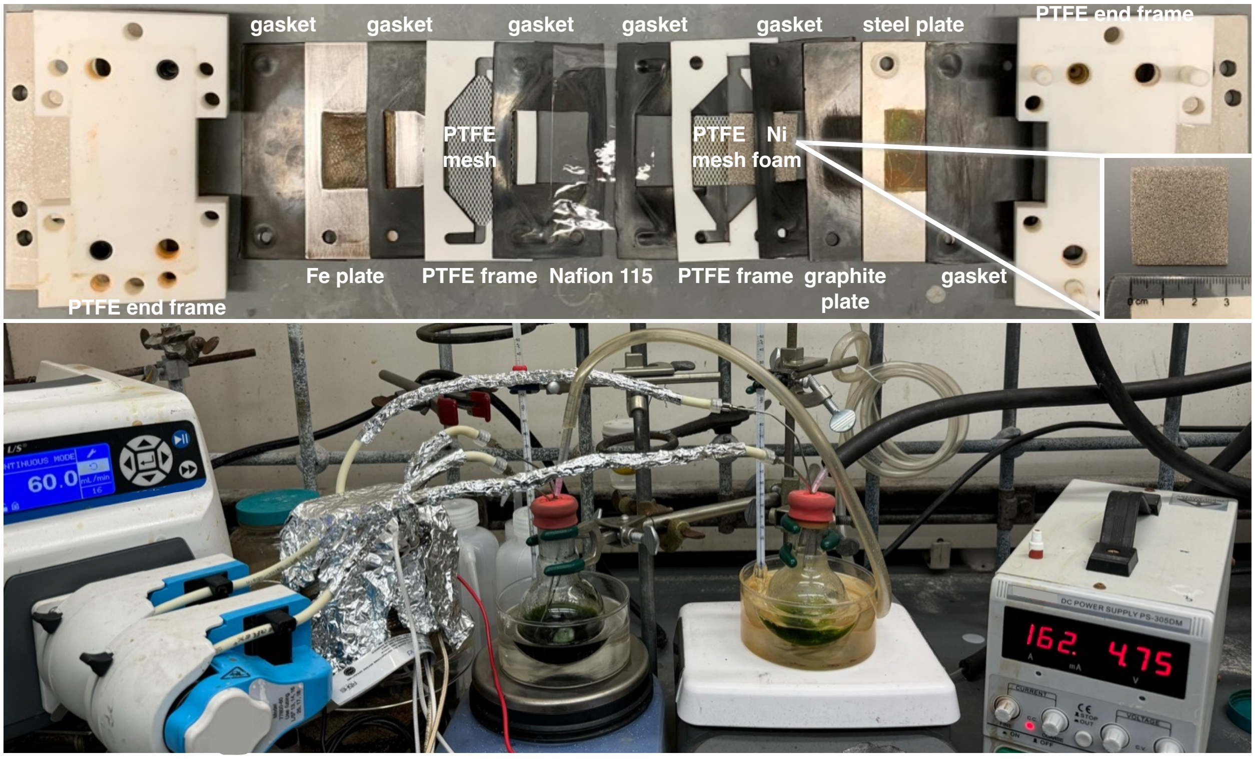

Figure S2. Graphic illustration of the components of the divided flow cell reactor (top) and flow electrolysis setup (bottom).

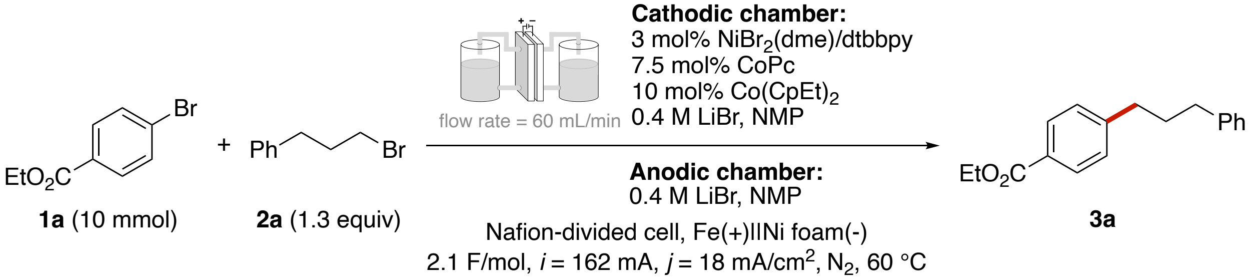

Two 100-mL round bottom flasks were dried in a 120 °C oven for 1 h and then transferred to a nitrogen-filled glovebox. To one of the round bottom flasks (cathodic reservoir) fitted with a cross-shaped stir bar was added $NiBr_2$(dme) (92.7 mg, 3 mol%), 4,4′-di-*tert*-butyl-2,2′-bipyridine (80.4 mg, 3 mol%), **1a** (2.29 g, 10 mmol, 1.0 equiv), **2a** (2.59 g, 13 mmol, 1.3 equiv), cobalt phthalocyanine (428.3 mg, 7.5 mol%), bis(ethylcyclopentadienyl)cobalt(II) (245 mg, 161.6 µL, 10 mol%), LiBr (696 mg, 8 mmol, 0.4 M), 1,3,5-trimethoxybenzene (560 mg, 3.3 mmol, 0.33 equiv), and anhydrous NMP (20 mL). Then to another 100-mL round bottom flask (anodic reservoir) fitted with a cross-shaped stir bar was added LiBr (1.39 g, 16 mmol, 0.4 M) and anhydrous NMP (40 mL). The two reservoirs were sealed with rubber septa. To each flask was introduced a thin Teflon tube to allow continuous nitrogen bubbling after removal from the glovebox. The flasks were removed from the glovebox and both cathodic and anodic solutions were stirred at 600 rpm for 10 min in an oil bath heated to 80 °C to allow full dissolution of LiBr and exclusion of adventitious oxygen. The flow cell reactor was wrapped with heat tape heated to 80 °C and covered with aluminum foil to help maintain the desired temperature in the cell chambers. Note that a temperature greater than that used in batch (**GP 1**) was applied to account for the heat lost during the solution transfer between the reservoirs and the flow reactor. Both cathode and anode solutions were pushed via a peristaltic pump to pass through the divided flow cell, with a flow rate of 60 mL min^{-1}. An infrared laser thermometer was used to measure the temperature of solutions in both reservoirs to be stable at 60 °C. After that, the reaction was electrolyzed under a constant current electrolysis at 162 mA (18 mA/cm^2) for 3.5 h (2.1 F/mol) at 60 °C.

When the electrolysis was done, the resultant reaction mixture was cooled to room temperature, and 5 mL of acetone was pumped through both chambers to remove any leftover reaction mixture inside the reactor. A 100 µL aliquot of the cathodic solution was diluted with 500 µL $CDCl_3$ and filtered through a short plug of silica (2.5 cm thick × 7 mm diameter) into a test tube, and the silica plug was washed with additional 1 mL $CDCl_3$. Then 500 µL of the resultant solution was added to an NMR sample tube, and the sample was analyzed by ^{1}H NMR spectroscopy and the yield was determined using 1,3,5-trimethoxybenzene as the internal standard. For this reaction, a 96% yield of the desired cross-coupled product **3a** was obtained, with 2% of aryl dimer as the side product.

3. Reaction Condition Optimization and Additional Screenings

3.1 Optimization of reaction condition

Table S1. Overview of reaction optimizations[a]

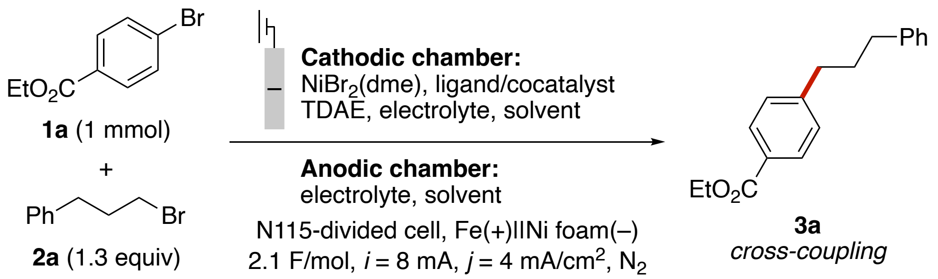

Entry	Catalysts	Mediator	Electrolyte	Solvent	Temp.	Yield (%)
**1**	7 mol% $NiBr_2$(dme) dtbbpy/ttbtpy (1:4)	20 mol% TDAE	$TBAPF_6$	DMA	r.t.	23
**2**	7 mol% $NiBr_2$(dme) dtbbpy/ttbtpy (1:4)	20 mol% TDAE	LiBr	NMP	r.t.	35
**3**	7 mol% $NiBr_2$(dme) dtbbpy/ttbtpy (1:1)	20 mol% TDAE	LiBr	NMP	r.t.	43
**4**	7 mol% $NiBr_2$/dtbbpy 5 mol% CoPc	20 mol% TDAE	LiBr	NMP	r.t.	52
**5**	7 mol% $NiBr_2$/dtbbpy 5 mol% CoPc	20 mol% TDAE	LiBr	NMP	60 °C	83
**6**	1 mol% $NiBr_2$/dtbbpy 2.5 mol% CoPc	10 mol% TDAE	LiBr	NMP	60 °C	**85**

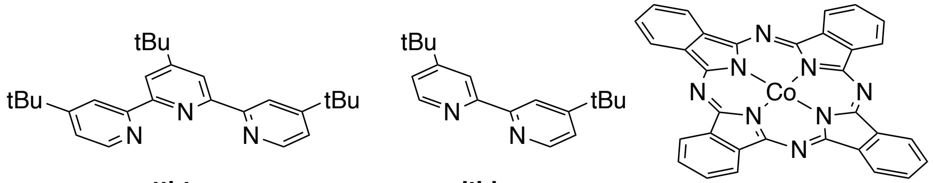

ttbtpy **dtbbpy** **CoPc**

[a]Yields were determined by ^{1}H NMR spectroscopy of the crude reaction mixture following the "Work-up for NMR analysis" procedure described in **GP 1**, with 1,3,5-trimethoxybenzene as an internal standard. Yields of homocoupled dimers are reported with respect to the stoichiometry of the substrate, i.e., the maximal theoretical **1aa** yield is 100%. Cross-selectivity was defined as **3a** yield:(**1aa** yield + **1a–H** yield). MB = mass balance.

Table S2. Initial screening of solvents and electrolytes[a]

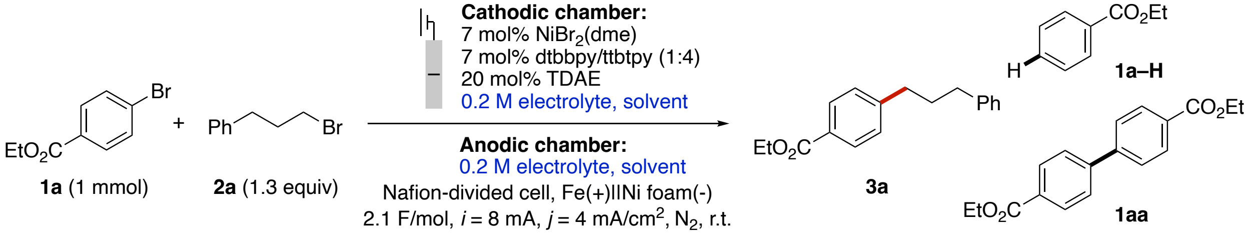

Entry	Electrolyte	Solvent	**1a** (%)	**2a** (%)	**3a** (%)	**1aa** (%)	**1a–H** (%)	Cross-selectivity	MB (%)
1	$TBAPF_6$	DMA	2	5	23	62	4	0.4:1	91
2	$TBAPF_6$	DMF	0	0	22	59	13	0.3:1	94
3	$TBAPF_6$	NMP	0	0	26	58	7	0.5:1	98
4	$TBAPF_6$	MeCN	46	11	14	23	10	0.4:1	93
5	LiBr	NMP	11	0	34	42	12	0.6:1	98
6	LiBr	DMA	0	0	26	45	7	0.9:1	101
7	$LiBF_4$	NMP	10	0	32	54	2	0.6:1	98
8	$LiPF_6$	NMP	6	0	31	52	7	0.5:1	96
9	TBABr	NMP	0	0	23	59	11	0.3:1	93

[a]Yields were determined by ^1H NMR spectroscopy of the crude reaction mixture following the "Work-up for NMR analysis" procedure described in **GP 1**, with 1,3,5-trimethoxybenzene as an internal standard. Yields of homocoupled dimers are reported with respect to the stoichiometry of the substrate, i.e., the maximal theoretical **1aa** yield is 100%. Cross-selectivity was defined as **3a** yield:(**1aa** yield + **1a–H** yield). MB = mass balance.

Table S3. Initial screening of dtbbpy/ttbtpy ratios[a]

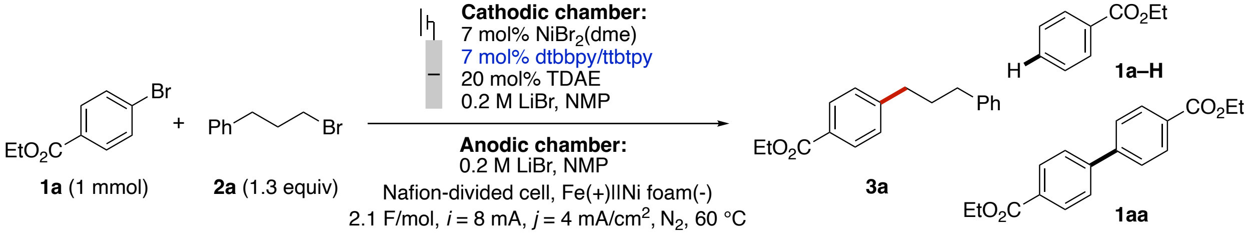

Entry	dtbbpy/ttbtpy ratio	**1a** (%)	**2a** (%)	**3a** (%)	**1aa** (%)	**1a–H** (%)	Cross-selectivity	MB (%)
1	0:10	19	0	8	31	42	0.1:1	100
2	1:9	17	0	17	47	17	0.3:1	98
3	2:8	12	0	35	42	12	0.6:1	101
4	3:7	36	16	41	5	22	1.5:1	104
5	4:6	39	28	30	9	21	1.0:1	99
6	5:5	29	36	43	16	11	1.6:1	99
7	8:2	26	30	38	19	11	1.3:1	94
8	10:0	36	51	29	29	6	0.8:1	100

[a]Yields were determined by ^{1}H NMR spectroscopy of the crude reaction mixture following the "Work-up for NMR analysis" procedure described in **GP 1**, with 1,3,5-trimethoxybenzene as an internal standard. Yields of homocoupled dimers are reported with respect to the stoichiometry of the substrate, i.e., the maximal theoretical **1aa** yield is 100%. Cross-selectivity was defined as **3a** yield:(**1aa** yield + **1a–H** yield). MB = mass balance.

Table S4. Screening of XAT reagents[a]

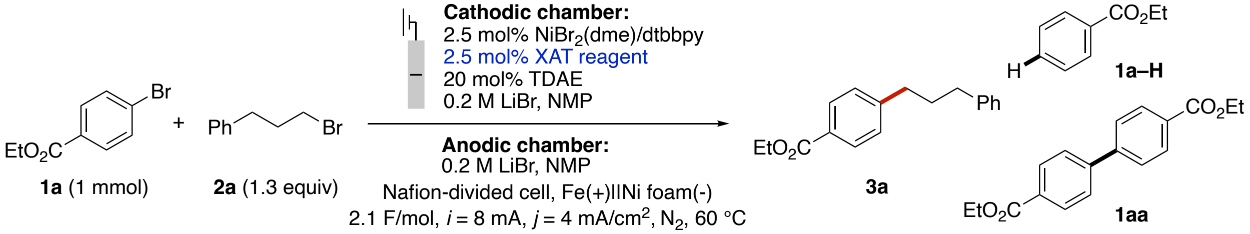

Entry	XAT reagent	1a (%)	2a (%)	3a (%)	1aa (%)	1a–H (%)	Cross-selectivity	MB (%)
1	CoPc	0	0	43	50	0	0.9:1	93
2	FePc	20	0	10	24	14	0.3:1	68
3	Co(Ph-por)	24	0	31	27	13	0.8:1	95
4	Fe(Ph-por)Cl	2	0	17	29	23	0.3:1	71
5	Co(Et-por)	4	0	16	31	25	0.3:1	76
6	Co(MeOPh-por)	11	0	36	10	13	1.6:1	70
7	Co(salen)	0	0	25	52	9	0.4:1	86
8	Cp_2TiCl_2	6	4	19	39	30	0.3:1	98
9	$CrCl_3$	21	16	3	20	7	0.1:1	67

[a]Yields were determined by ^{1}H NMR spectroscopy of the crude reaction mixture following the "Work-up for NMR analysis" procedure described in **GP 1**, with 1,3,5-trimethoxybenzene as an internal standard. Yields of homocoupled dimers are reported with respect to the stoichiometry of the substrate, i.e., the maximal theoretical **1aa** yield is 100%. Cross-selectivity was defined as **3a** yield:(**1aa** yield + **1a–H** yield). MB = mass balance.

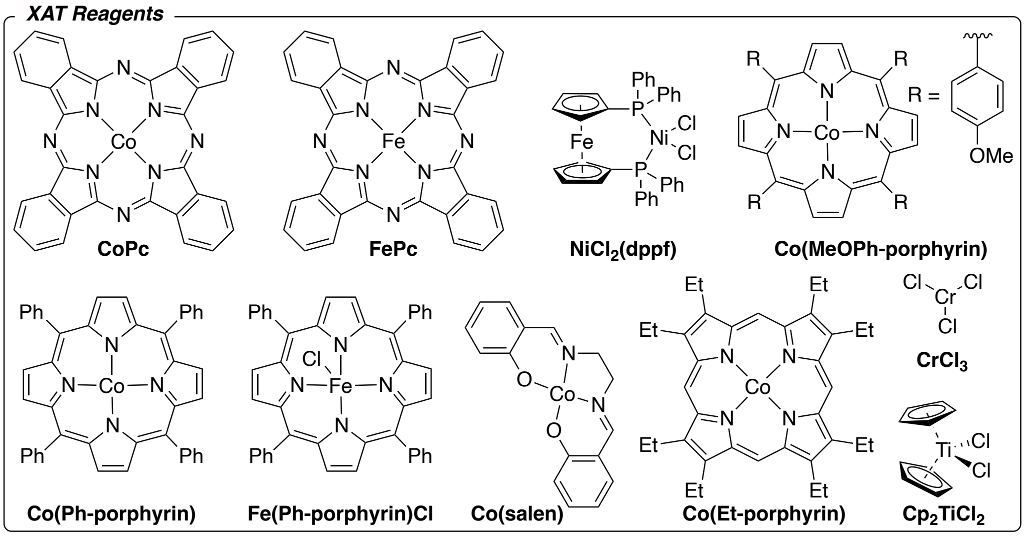

Table S5. Screening of Ni/dtbbpy and CoPc loadings in undivided cells[a]

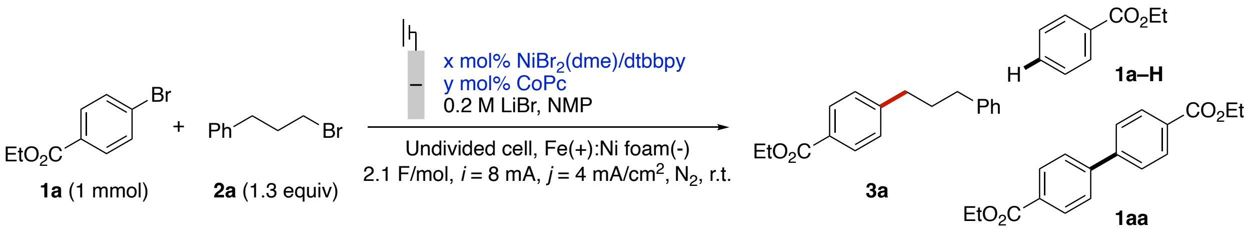

Entry	Ni/dtbbpy (mol%)	CoPc (mol%)	**1a** (%)	**2a** (%)	**3a** (%)	**1aa** (%)	**1a–H** (%)	Cross-selectivity	MB (%)
1	2.5	0.5	0	0	24	67	7	0.3:1	98
2	2.5	1	0	0	31	63	3	0.5:1	97
3	2.5	2.5	0	0	33	58	7	0.5:1	98
4	2.5	5	0	0	36	54	9	0.6:1	99
5	1	2.5	0	0	49	45	7	0.9:1	101
6	7	5	0	0	39	51	9	0.7:1	99
7[b]	7	5	0	0	52	37	9	1.1:1	98
8[b,c]	7	5	0	0	83	10	7	4.9:1	100

[a]Yields were determined by ^{1}H NMR spectroscopy of the crude reaction mixture following the "Work-up for NMR analysis" procedure described in **GP 1**, with 1,3,5-trimethoxybenzene as an internal standard. Yields of homocoupled dimers are reported with respect to the stoichiometry of the substrate, i.e., the maximal theoretical **1aa** yield is 100%. Cross-selectivity was defined as **3a** yield:(**1aa** yield + **1a–H** yield). MB = mass balance. [b]With 20 mol% TDAE in a standard divided cell. [c]At 60 °C.

Table S6. Screening of ET-mediators with optimized reaction condition[a]

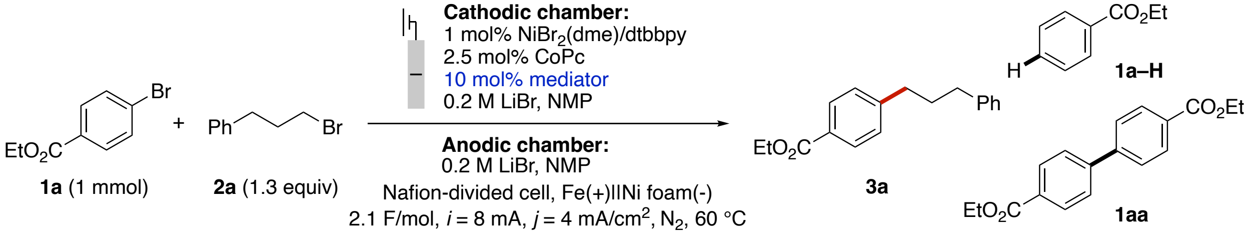

Entry	ET mediator	1a (%)	2a (%)	3a (%)	1aa (%)	1a–H (%)	Cross-selectivity	MB (%)
1	none	0	0	45	52	3	0.8:1	100
2	TME	8	0	61	35	7	1.9:1	101
3	TPiE	4	0	71	20	4	3.0:1	99
4	TDAE	0	0	78	19	2	3.7:1	99
5	$CoCp_2$	0	0	80	10	4	5.7:1	94
6	$Co(CpEt)_2$	0	0	85	9	5	6.1:1	99
7	$Co(CpMe_4)_2$	5	0	48	26	17	0.9:1	96
8	$CoCp^*_2$	11	0	42	23	18	0.9:1	94

[a]Yields were determined by ^1H NMR spectroscopy of the crude reaction mixture following the "Work-up for NMR analysis" procedure described in **GP 1**, with 1,3,5-trimethoxybenzene as an internal standard. Yields of homocoupled dimers are reported with respect to the stoichiometry of the substrate, i.e., the maximal theoretical **1aa** yield is 100%. Cross-selectivity was defined as **3a** yield:(**1aa** yield + **1a–H** yield). MB = mass balance.

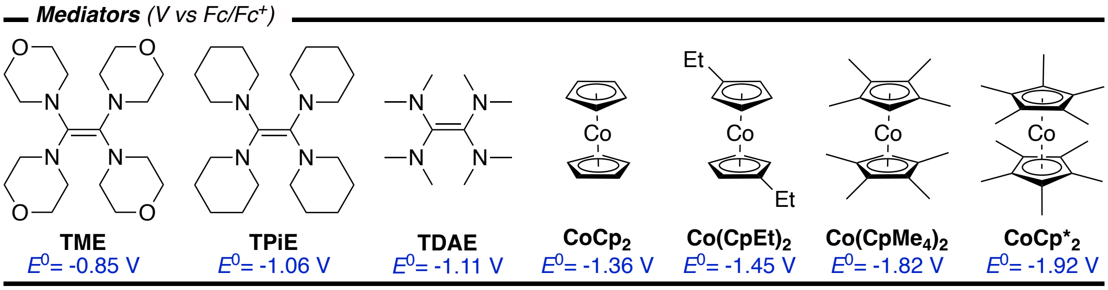

Table S7. Other screenings and control experiments[a]

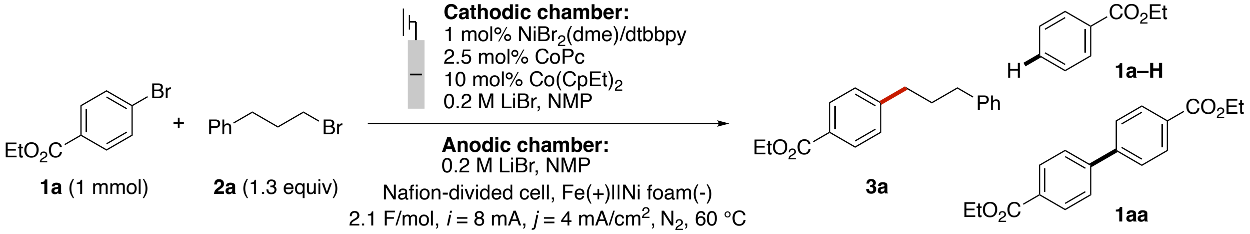

Entry	Variations	1a (%)	2a (%)	3a (%)	1aa (%)	1a–H (%)	Cross-selectivity	MB (%)
1	none	0	0	85	9	5	6.1:1	99
2	no $Co(CpEt)_2$	0	0	45	52	3	0.8:1	100
3	no Ni/dtbbpy	63	54	0	3	35	-	101
4	no CoPc	9	52	12	56	26	0.1:1	103
5	no CoPc, 2 mA/cm^2	0	0	79	22	0	3.6:1	101
6	50 °C instead of 60 °C	0	0	59	34	6	1.5:1	99
7	r.t. instead of 60 °C	0	0	39	45	17	0.6:1	101
8	80 °C instead of 60 °C	0	0	82	10	6	5.1:1	98
9	SS instead of Fe anode	3	0	66	25	7	2.1:1	101
10	Zn instead of Fe anode	3	0	75	18	4	3.4:1	100
11	no electrolysis	98	126	0	0	2	-	100
12[b]	no electrolysis, 2.2 equiv $Co(CpEt)_2$	52	70	45	0	0	-	97
13	in undivided cell	0	0	71	21	8	2.4:1	100
14	12 mA instead of 8 mA	0	7	91	7	0	13:1	98
15	12 mA instead of 8 mA, no $Co(CpEt)_2$	27	49	35	26	11	0.9:1	99
16	12 mA instead of 8 mA, no $Co(CpEt)_2$, 4 mol% Ni/dtbbpy, 10 mol% CoPc	30	9	38	30	5	1.1:1	103

[a]Yields were determined by ^{1}H NMR spectroscopy of the crude reaction mixture following the "Work-up for NMR analysis" procedure described in **GP 1**, with 1,3,5-trimethoxybenzene as an internal standard. Yields of homocoupled dimers are reported with respect to the stoichiometry of the substrate, i.e., the maximal theoretical **1aa** yield is 100%. Cross-selectivity was defined as **3a** yield:(**1aa** yield + **1a–H** yield). MB = mass balance. [b]Added 0.1 equiv $Co(CpEt)_2$ to the cell initially, followed by gradual, constant addition of the additional 2.1 equiv $Co(CpEt)_2$ over 7 h using a syringe pump.

Table S8. Screening of flow electrolysis conditions with a parallel plate reactor[a]

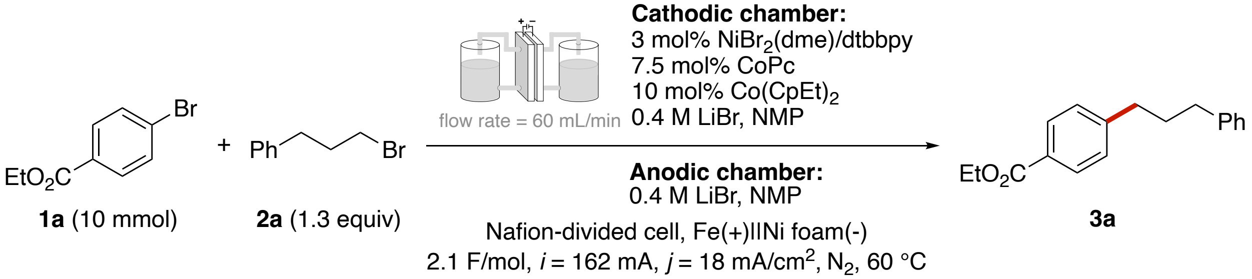

Entry	Variations	**1a** (%)	**2a** (%)	**3a** (%)	**1aa** (%)	**1a–H** (%)	Cross-selectivity	MB (%)
1	none	0	3	96	3	0	32:1	99
2	1 mol% Ni/dtbbpy, 2.5 mol% CoPc, 12 mA/cm^2	0	0	81	14	0	5.8:1	95
3	2 mol% Ni/dtbbpy, 5 mol% CoPc, 12 mA/cm^2	0	2	92	8	0	11.5:1	100
4	4 mol% Ni/dtbbpy, 10 mol% CoPc, 24 mA/cm^2	57	26	2	2	1	0.7:1	62
5	4 mol% Ni/dtbbpy, 10 mol% CoPc, 20 mol% Co(CpEt)$_2$, 24 mA/cm^2	37	0	33	17	5	1.5:1	92
6	100 mL/min flow rate, 24 mA/cm^2	45	9	19	4	22	0.7:1	90
7	No Co(CpEt)$_2$	52	16	17	22	7	0.6:1	98
8	No Co(CpEt)$_2$, 3 mA/cm^2	0	0	73	22	0	3.3:1	95
9	0.2 M LiBr	6	0	68	18	0	3.8:1	92

[a]Yields were determined by ^{1}H NMR spectroscopy of the crude reaction mixture following the "Work-up for NMR analysis" procedure described in **GP 2**, with 1,3,5-trimethoxybenzene as an internal standard. Yields of homocoupled dimers are reported with respect to the stoichiometry of the substrate, i.e., the maximal theoretical **1aa** yield is 100%. Cross-selectivity was defined as **3a** yield:(**1aa** yield + **1a–H** yield). MB = mass balance.

Table S9. Screening of optimized condition with or without a mediator[a]

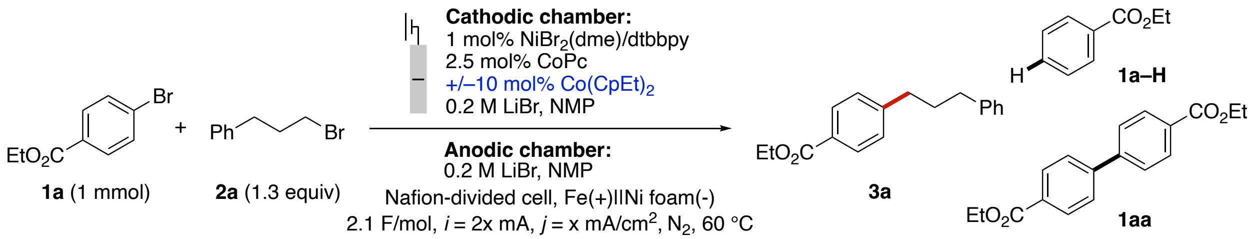

Entry	Current density (mA/cm^2)	1a (%)	3a (%)	1aa (%)	1a–H (%)	Cross-selectivity	MB (%)
+ $Co(CpEt)_2$	4	0	**80**	14	5	4.2:1	99
	8	0	**85**	15	0	5.7:1	100
	12	0	**91**	5	3	11.4:1	99
	14	0	**75**	26	4	2.5:1	105
	16	0	**70**	19	8	2.6:1	97
– $Co(CpEt)_2$	4	3	**46**	45	4	0.9:1	98
	8	0	**44**	29	17	1.0:1	90
	12	46	**20**	18	5	0.9:1	89
	14	30	**16**	18	19	0.4:1	83
	16	21	**8**	5	29	0.2:1	63

[a]Yields were determined by ^{1}H NMR spectroscopy of the crude reaction mixture following the "Work-up for NMR analysis" procedure described in **GP 2**, with 1,3,5-trimethoxybenzene as an internal standard. Yields of homocoupled dimers are reported with respect to the stoichiometry of the substrate, i.e., the maximal theoretical **1aa** yield is 100%. Cross-selectivity was defined as **3a** yield:(**1aa** yield + **1a–H** yield). MB = mass balance.

3.2 Comparison with overcharge protector strategy for improving current density

A straightforward comparison between the overcharge protection strategy used by the Sevov group[1] and the ET-mediator strategy (introduced in this work) was conducted. Conceptually, these two strategies target the cross-selectivity issue of high current density Ni-catalyzed electrochemical XEC from different directions: for the overcharge protection strategy, the overcharge protector is more reducing than the productive catalyst reduction. An undivided cell is used, and the overcharge protector is added to avoid catalyst decomposition and **improve the catalyst stability** (Figure S3A); on the other hand, the ET-mediator strategy uses a compound that is less reducing than the catalyst reduction potential and will be the species first reduced in the system. In this case, a divided cell is used, and the mediator is added to the solution to **improve the catalyst performance** and help with the system turnover (Figure S3B).

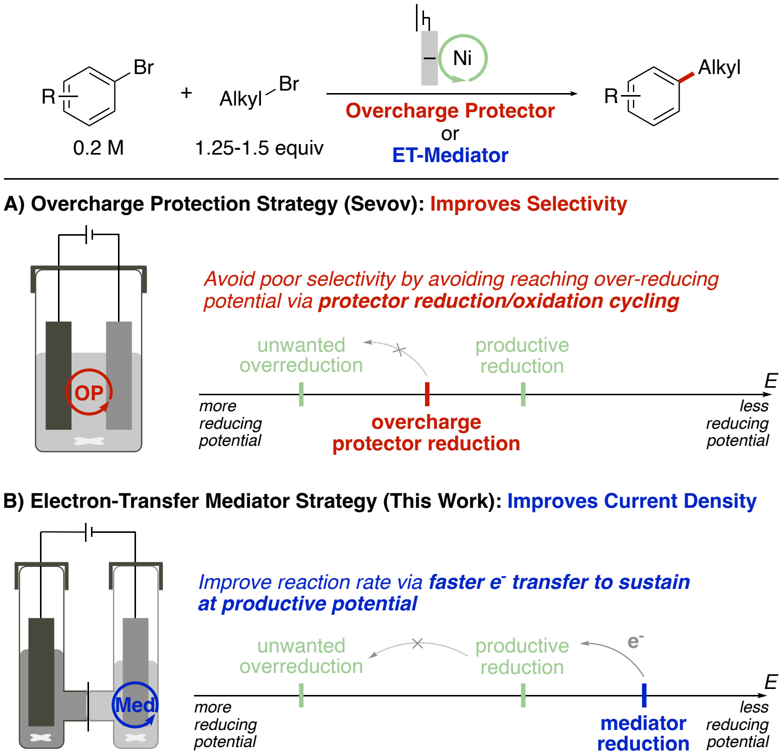

Figure S3. Graphic depictions of two representative strategies to improve selectivity in high current density Ni-catalyzed electrochemical XEC. A) Overcharge protector strategy that works by improving catalyst stability. B) ET-mediator strategy (this work) that works by improving catalyst performance.

To compare these two strategies, their yields and Faradaic efficiencies as a function of current density were evaluated. The reported procedure was strictly followed for the overcharge protector system[1] (see below for detailed experimental procedure), and **GP 1** was followed for the ET-mediator system. The same model substrates were used for a direct comparison, and a longer reaction time (more charge passed) was used as needed until the aryl substrate was fully converted in each case. The exact charge passed to reach full aryl substrate conversion in each reaction is documented in Table S7. The yields and Faradaic efficiencies at current densities of 2, 4, 6, 7, and 8 mA/cm^2 are shown in Figure S4. In this screening, the overcharge protector system demonstrated a drop in both yield and Faradaic efficiency at ≥6 mA/cm^2 (red data in Figure S4), while the ET-

mediator maintained a good to decent yield and Faradaic efficiency throughout all screened current densities (blue data in Figure S4).

Overcharge protection system reaction set-up: In a nitrogen-filled glovebox, an in-house made 6 mL undivided cell was charged with catalyst (MeBPI)Ni-OAc (11.1 mg, 0.025 mmol, 0.025 equiv), overcharge protector $(MeBPI)_2Ni$ (35.6 mg, 0.05 mmol, 0.05 equiv), KPF_6 (73.6 mg, 0.40 mmol, 0.1 M), **1a** (1 mmol, 1 equiv), **2a** (1.5 mmol, 1.5 equiv), DMA (4 mL), and a magnetic stir bar. A 1.0 cm × 1.0 cm nickel foam cathode and a zinc anode were inserted through the septa cap, and the undivided cell was sealed and removed from the glovebox. The reaction mixture was stirred at 1100 rpm on a magnetic stir plate at room temperature, and a thin Teflon tube was introduced into the cell to allow continuous nitrogen bubbling. The reaction mixture was electrolyzed under constant current electrolysis at varied applied currents.

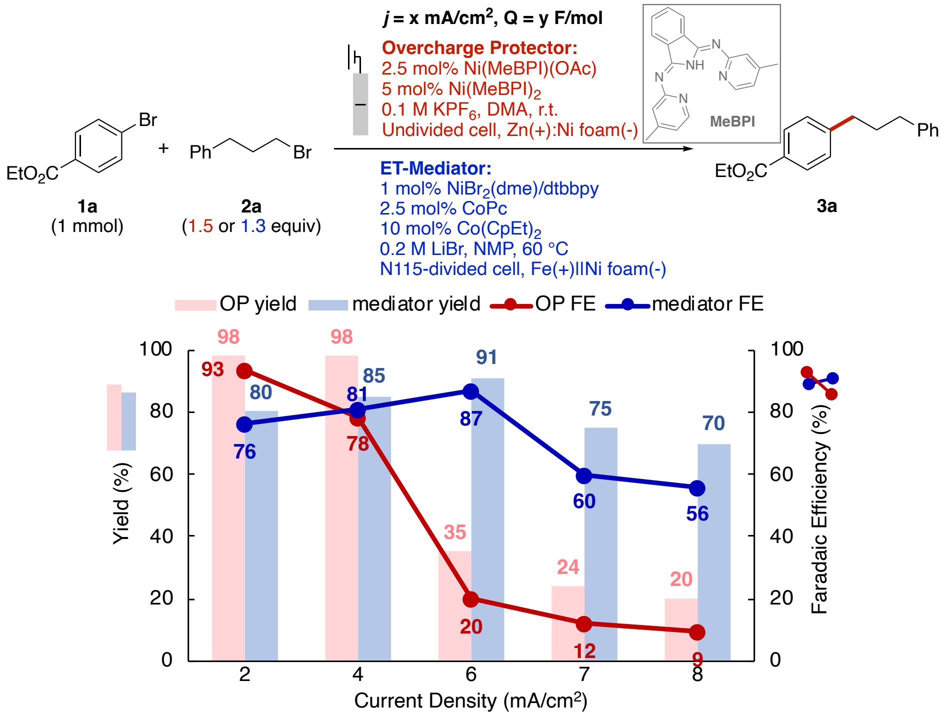

Figure S4. Yields and Faradaic efficiencies for the overcharge protector strategy (red) and the ET-mediator strategy (blue) carried out at varying current density. Yields were determined by ^1H NMR spectroscopy of the crude reaction mixture following the "Work-up for NMR analysis" procedure described in **GP 1**, with 1,3,5-trimethoxybenzene as an internal standard. Faradaic efficiencies were calculated based on the charge passed and the experimental yields assuming 2 e^-/mol product are consumed productively.

Table S10. Charge passed for each experiment to achieve full aryl substrate conversion.

System	Charge passed at varied current density (F/mol)				
	2 mA/cm²	4 mA/cm²	6 mA/cm²	7 mA/cm²	8 mA/cm²
Overcharge Protector	2.1	2.5	2.5	2.8	3.0
ET-Mediator	2.1	2.1	2.1	2.1	2.1

4. Improving Literature-Reported Methods with ET-Mediator

Additional attempts at improving yield and selectivity for other literature-reported Ni-catalyzed electrochemical XEC strategies were made by adding 10 mol% $Co(CpEt)_2$ as the ET-mediator to the reported optimized systems. Three reported systems were assessed, one from Weix and co-workers published in 2016,[2] one from Hansen and co-workers published in 2019,[3] and the system reported by us shown in Figure 1B in the main text.[4] Their reported conditions and procedures were adapted and translated to a divided cell setup for each system, and $Co(CpEt)_2$ was added together with the substrates (see below for detailed experimental procedures). To calibrate the results and determine whether any change in yield is simply due to changing the cell configuration, a "without mediator" entry was also conducted in divided cells. Results from both conditions showed that without the ET-mediator, the reactions carried out in the divided cell yielded similar results to the literature yields. However, by simply adding 10 mol% $Co(CpEt)_2$ to the system, substantial increases in yield were obtained for all tested conditions and substrates (Figure S5 and S6).

Weix 2016[2] system reaction set-up: An in-house made divided cell was transferred to a nitrogen-filled glovebox. To the cathodic chamber of the divided cell equipped with a cross-shaped stir bar was added $NiBr_2$(dme) (21.6 mg, 7 mol%), 4,4′-di-*tert*-butyl-2,2′-bipyridine (18.8 mg, 7 mol%), aryl bromide substrate **1** (1 mmol, 1.0 equiv), benzyl chloride substrate **4** (2 mmol, 2 equiv), LiCl (34 mg, 0.8 mmol, 0.2 M), bis(ethylcyclopentadienyl)cobalt(II) (24.5 mg, 16.2 µL, 10 mol%), and DMA (4 mL). Then to the anodic chamber of the divided cell fitted with a cross-shaped stir bar was added LiCl (68 mg, 1.6 mmol, 0.2 M) and anhydrous DMA (8 mL). The cathodic chamber was then equipped with a 1.0 cm × 1.0 cm Ni foam cathode, and the anodic chamber was equipped with a Fe rod anode. The two chambers were sealed with rubber septa and removed from the glovebox. To each chamber was introduced a thin Teflon tube to allow continuous nitrogen bubbling. The reaction mixture was stirred at 1100 rpm for 30 min in a sand bath heated to 80 °C to allow full dissolution of LiCl and exclusion of adventitious oxygen. After that, the reaction mixture was electrolyzed under a constant current electrolysis at 8 mA for 8.4 h (2.5 F/mol) at 80 °C.

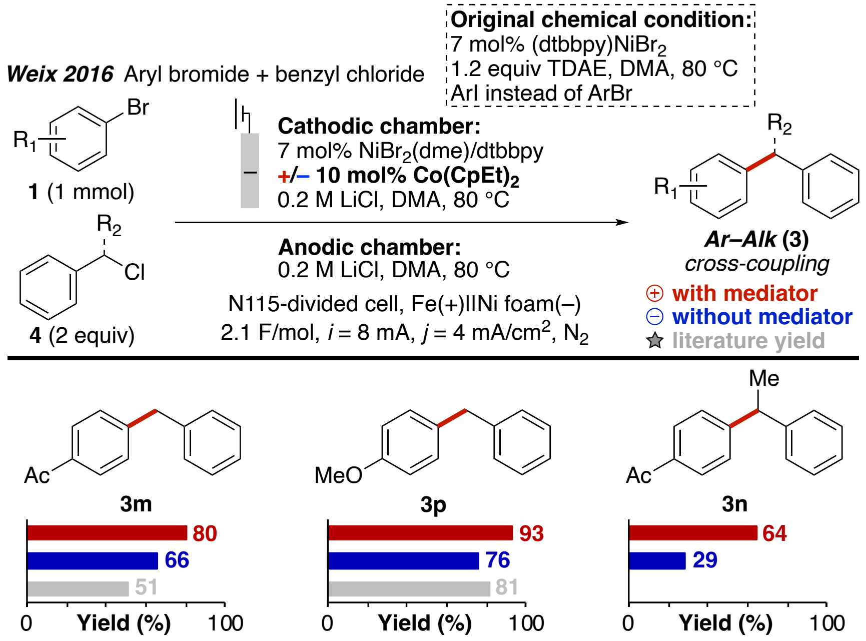

Figure S5. Yields with ET-mediator (red) and without ET-mediator (blue) in a divided cell compared to literature yields (gray). Yields were determined by ^{1}H NMR spectroscopy of the crude reaction mixture following the "Work-up for NMR analysis" procedure described in **GP 1**, with 1,3,5-trimethoxybenzene as an internal standard.

Hansen 2019[3] system reaction set-up: In a nitrogen-filled glovebox, $NiBr_2$(dme) (21.6 mg, 7 mol%), 4,4′-di-*tert*-butyl-2,2′-bipyridine (15.0 mg, 5.6 mol%), 4,4′,4″-tri-*tert*-butyl-2,2′:6′,2″-terpyridine (5.6 mg, 1.4 mol%), and anhydrous NMP (5 mL) were added to a 2-dram vial equipped with a PTFE-coated stir-bar. This solution was stirred at 800 rpm in the glove box for 1 h to allow complexation of Ni with the ligand.

To the cathodic chamber of the divided cell fitted with a cross-shaped stir bar was added aryl bromide substrate **1** (1 mmol, 1.0 equiv), alkyl bromide substrate **6** (1.2 mmol, 1.2 equiv), LiBr (87 mg, 1 mmol, 0.2 M), bis(ethylcyclopentadienyl)cobalt(II) (24.5 mg, 16.2 µL, 10 mol%), and the pre-stirred catalyst solution (5 mL). Then to the anodic chamber of the divided cell fitted with a cross-shaped stir bar was added LiBr (139.2 mg, 1.6 mmol, 0.2 M) and anhydrous NMP (8 mL). The cathodic chamber was then equipped with a 1.0 cm × 1.0 cm Ni foam cathode, and the anodic chamber was equipped with a Fe rod anode. The two chambers were sealed with rubber septa and removed from the glovebox. To each chamber was introduced a thin Teflon tube to allow continuous nitrogen bubbling. The reaction mixture was stirred at 1100 rpm for 30 min in a sand bath heated to 60 °C to allow full dissolution of LiBr and exclusion of adventitious oxygen. After that, the reaction mixture was electrolyzed under a constant current electrolysis at 8 mA for 7 h (2.1 F/mol) at 60 °C.

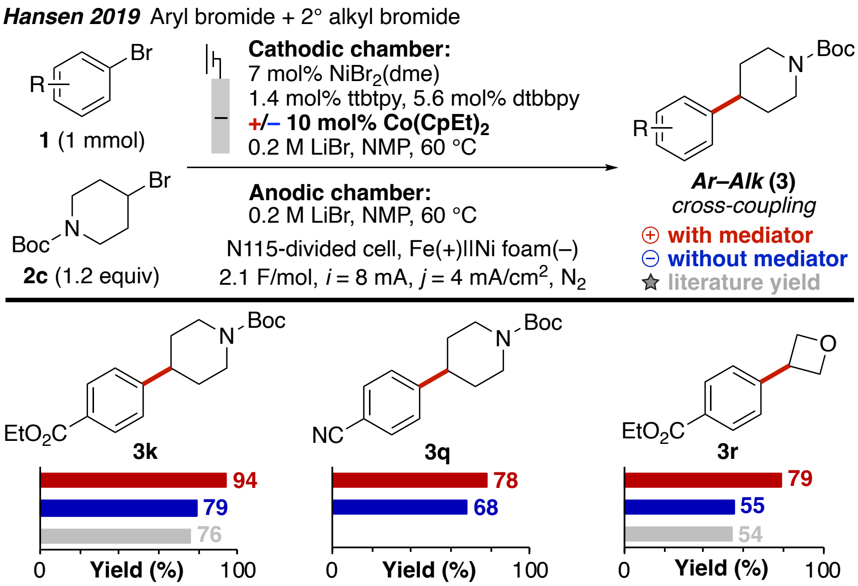

Figure S6. Yields with ET-mediator (red) and without ET-mediator (blue) in a divided cell compared to literature yields (gray). Yields were determined by ^1H NMR spectroscopy of the crude reaction mixture following the "Work-up for NMR analysis" procedure described in **GP 1**, with 1,3,5-trimethoxybenzene as an internal standard.

Figure 1B of the manuscript presents an example of a recent current density study of a Ni-catalyzed eXEC flow electrolysis where the reaction failed at or above 8 mA/cm^2.[4] We assessed whether our mediator strategy could rescue the reactivity at higher current densities in flow electrolysis (Figure S7). In the setup used for our current study, $Co(CpEt)_2$ was added to the cathodic reservoir, and the H_2 anode was replaced by a Fe sacrificial anode instead to simplify the reaction setup.

Current density study setup: In a nitrogen-filled glovebox, $NiBr_2 \cdot 3H_2O$ (218.0 mg, 10 mol%), 4,4′-di-*tert*-butyl-2,2′-bipyridine (188.7 mg, 8.8 mol%), 4,4′,4″-tri-*tert*-butyl-2,2′:6′,2″-terpyridine (70.8 mg, 2.2 mol%), and anhydrous NMP (20 mL) were added to a 6-dram vial equipped with a PTFE-coated stir-bar. This solution was stirred at 800 rpm in the glove box for 1 h to allow complexation of Ni with the ligand.

Two 100-mL round bottom flasks were dried in a 120 °C oven for 1 h and then transferred to a nitrogen-filled glovebox. To one of the round bottom flasks (cathodic reservoir) fitted with a cross-shaped stir bar was added **5a** (1.62 g, 8 mmol, 1.0 equiv), **2f** (1.79 g, 1.29 mL, 12 mmol, 1.5 equiv), bis(ethylcyclopentadienyl)cobalt(II) (196 mg, 129.3 µL, 10 mol%), LiBr (1.04 g, 12 mmol, 0.6 M), 1,3,5-trimethoxybenzene (448 mg, 2.7 mmol, 0.33 equiv), and the pre-stirred catalyst solution (20 mL). Then to another 100-mL round bottom flask (anodic reservoir) fitted with a cross-shaped stir bar was added LiBr (2.09 g, 24 mmol, 0.6 M) and anhydrous NMP (40 mL). The two reservoirs were sealed with rubber septa. To each flask was introduced a thin Teflon tube to allow continuous nitrogen bubbling after removal from the glovebox. The flasks were removed from the glovebox and both cathodic and anodic solutions were stirred at 600 rpm for 10 min to allow full dissolution of LiBr and exclusion of adventitious oxygen. Both cathode and anode solutions were pushed via a peristaltic pump to pass through the divided flow cell, with a flow rate of 20 mL min^{-1}. After that, the reaction was electrolyzed under a constant current electrolysis at 18 mA (2 mA/cm^2), 36

mA (4 mA/cm^2), 72 mA (8 mA/cm^2), 108 mA (12 mA/cm^2), or 144 mA (16 mA/cm^2), for 3.5 F/mol of charge in each run.

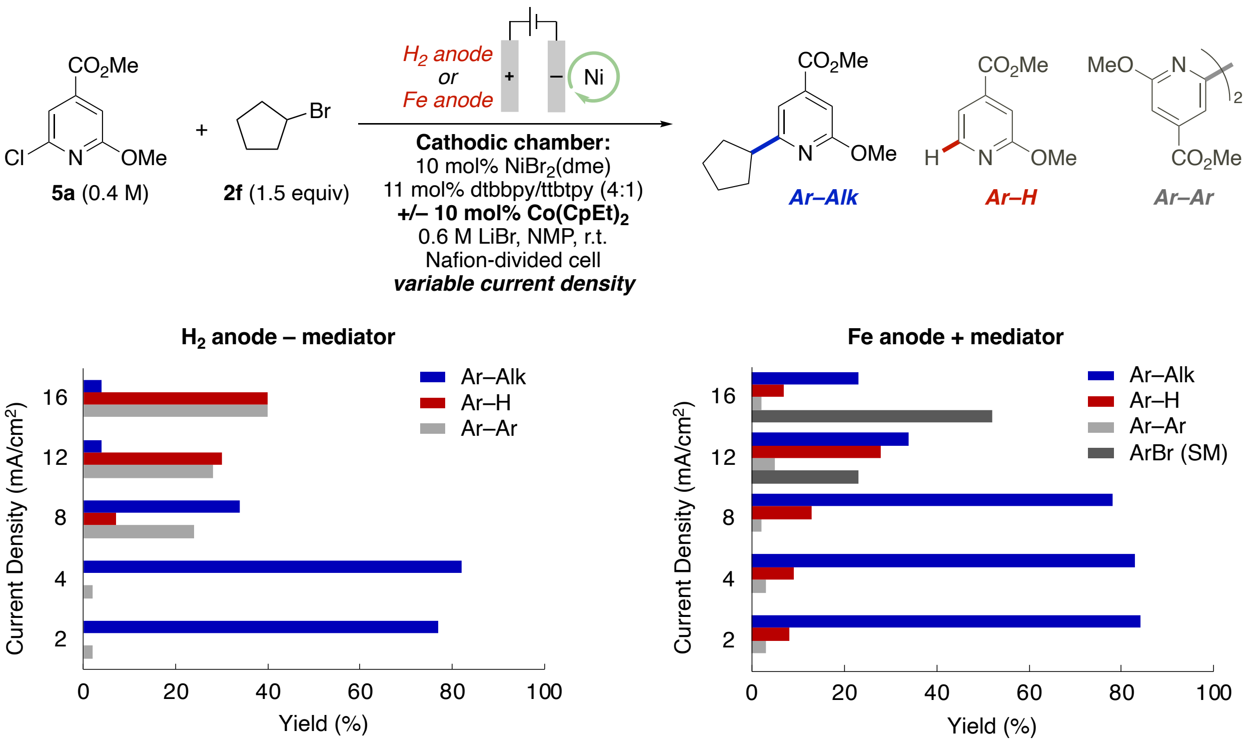

Figure S7. Reaction performance in flow electrolysis at different current densities reported in the literature without mediator (left) and assessed in this study with mediator (right). Yields were determined by ^{1}H NMR spectroscopy of the crude reaction mixture following the "Work-up for NMR analysis" procedure described in **GP 1**, with 1,3,5-trimethoxybenzene as an internal standard.

5. Time Course Analysis of Mediated- and Non-Mediated-Reactions and Mechanistic Cycle

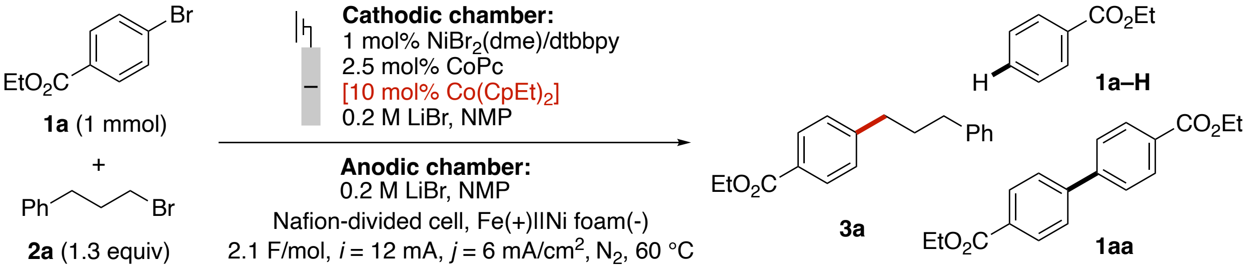

To further probe how the Co(CpEt)$_2$ mediator helps improve the reaction, two reactions of **1a** and **2a** following either **GP 1** or **GP 1-4** with some minor modifications were conducted to enable comparisons between mediated and non-mediated conditions. In the cathodic chamber of each reaction setup, a Ag/AgNO$_3$ reference electrode was included to allow monitoring of the working potential throughout the reaction. The reaction mixtures were electrolyzed using Bio-Logic BP-300 bipotentiostat under constant current electrolysis at 12 mA for 4.7 h (2.1 F/mol) at 60 °C. The working potential of each reaction was recorded.

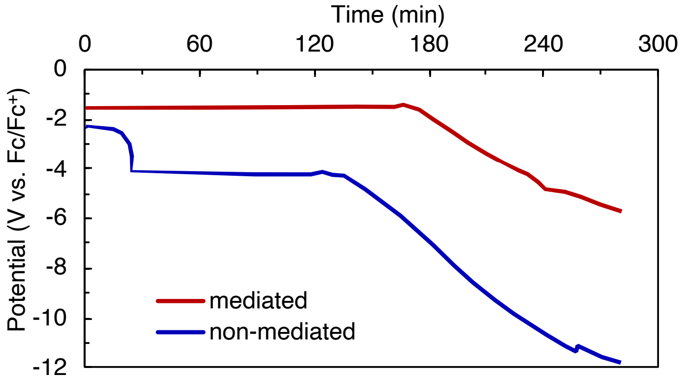

Figure S8. Monitored potential at the working electrode for optimized mediated condition (red) and non-mediated (without $Co(CpEt)_2$) condition (blue). The potentials were referenced to ferrocene/ferrocenium.

During the electrolysis, an aliquot of the reaction mixture (~50 µL) was taken, worked up following the procedure described in **GP 1** and analyzed via ^{1}H NMR spectroscopy every 30 min to monitor the progress of the reactions.

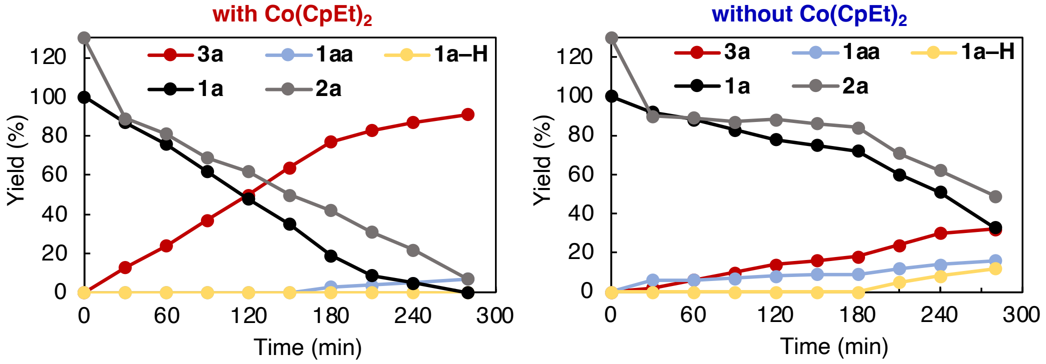

Figure S9. Time course of optimized reaction conditions at 6 mA/cm^2 current density, with $Co(CpEt)_2$ (left) or without $Co(CpEt)_2$ (right). Yields were determined by ^{1}H NMR spectroscopy of the crude reaction mixture following the "Work-up for NMR analysis" procedure described in **GP 1**, with 1,3,5-trimethoxybenzene as an internal standard. Yields of **1aa** determined with respect to the stoichiometry of the substrate, i.e., the maximal theoretical **1aa** yield is 100%.

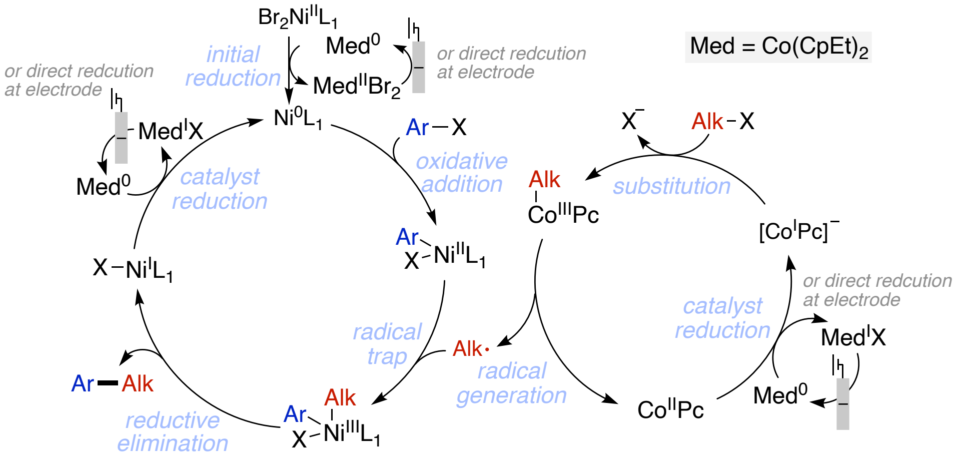

Figure S10. Proposed catalytic mechanism for the optimized reaction conditions, illustrating the distinct roles of the CoPc cocatalyst (alkyl halide activation) and cobaltocene mediator (Med, electron transfer).

6. Cyclic Voltammetry (CV) Studies

*6.1 CV studies of Ni/dtbbpy and Co(CpEt)₂, with or without model substrates **1a** and **2a***

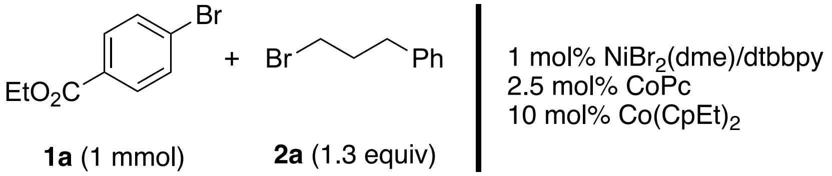

In the substrate scope, **3a** – **3j** were synthesized using a Ni/dtbbpy and CoPc catalyst system, so a CV study was carried out with **1a** and **2a** as the model substrates. However, CoPc was omitted from this study due to its insolubility.

In a nitrogen-filled glovebox, a stock solution containing 10 mM $NiBr_2$(dme) and 10 mM 4,4′-di-*tert*-butyl-2,2′-bipyridine in anhydrous NMP (20 mL) was prepared. This solution was stirred at 800 rpm in the glovebox for 3 h to allow complexation of Ni with the ligand. Then LiBr (0.1 M) was added to the solution and stirred until fully dissolved. The resultant solution was used as the stock solution for preparation of any CV solutions containing Ni/dtbbpy.

To avoid the chemical reduction of Ni/dtbbpy by $Co(CpEt)_2$, $Co(CpEt)_2$ was oxidized to $[Co(CpEt)_2]^+$ via constant current electrolysis at 5 mA for 64 min (1 F/mol) in a divided cell. A stock solution containing 10 mM $[Co(CpEt)_2]^+$ in NMP (20 mL) with LiBr (0.1 M) was prepared and used for preparation of any CV solutions containing $[Co(CpEt)_2]^+$.

Solutions for all CV studies were prepared according to the description in the caption of each figure. A glassy carbon (GC) working electrode, a platinum counter electrode, and a Ag/$AgNO_3$ reference electrode were used as a three-electrode configuration for all CV experiments (see "Electrochemical experiments" portion in "General Experimental Considerations" for details of the electrodes). A sand bath was used to heat the CV cell and reaction solutions to 60 °C.

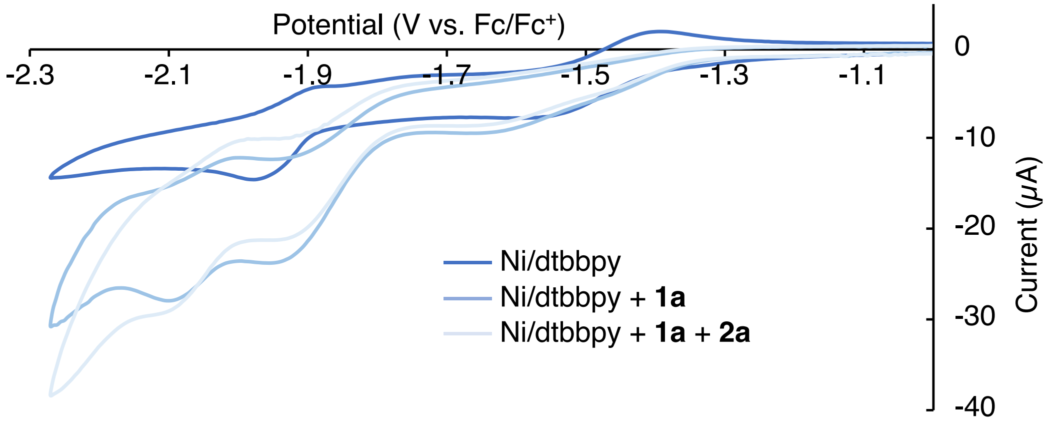

Figure S11. CVs of $NiBr_2$(dme)/dtbbpy only (dark blue), $NiBr_2$(dme)/dtbbpy + **1a** (blue), and $NiBr_2$(dme)/dtbbpy + **1a** + **2a** (light blue). Dark blue trace: 5.0 mM $NiBr_2$(dme)/dtbbpy. Blue trace: 5.0 mM $NiBr_2$(dme)/dtbbpy + 15.0 mM **1a**. Light blue trace: 5.0 mM $NiBr_2$(dme)/dtbbpy + 15.0 mM **1a** + 15.0 mM **2a**. All CVs were recorded in NMP (4 mL) with LiBr (0.1 M) as supporting electrolyte, under N_2 atmosphere, at 60 °C, scan rate = 100 mV/s, initially scanning towards a more negative potential.

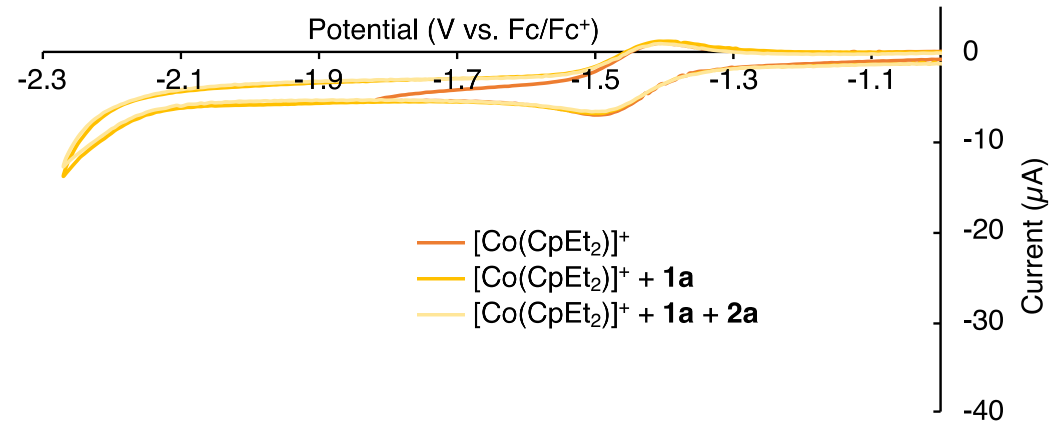

Figure S12. CVs of $[Co(CpEt)_2]^+$ only (orange), $[Co(CpEt)_2]^+$ + **1a** (yellow), and $[Co(CpEt)_2]^+$ + **1a** + **2a** (light yellow). Orange trace: 5.0 mM $[Co(CpEt)_2]^+$. Yellow trace: 5.0 mM $[Co(CpEt)_2]^+$ + 15.0 mM **1a**. Light yellow trace: 5.0 mM $[Co(CpEt)_2]^+$ + 15.0 mM **1a** + 15.0 mM **2a**. All CVs were recorded in NMP (4 mL) with LiBr (0.1 M) as supporting electrolyte, under N_2 atmosphere, at 60 °C, scan rate = 100 mV/s, initially scanning towards a more negative potential.

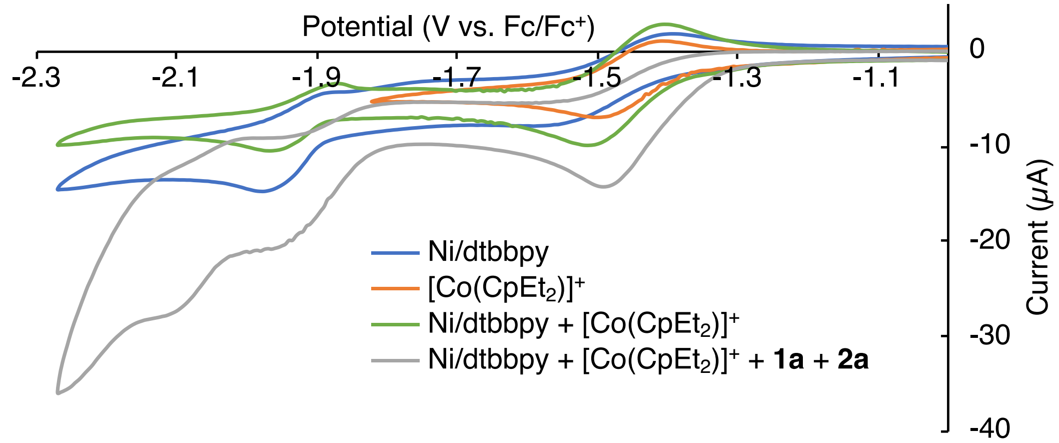

Figure S13. CVs of $NiBr_2$(dme)/dtbbpy only (blue), $[Co(CpEt)_2]^+$ only (orange), $NiBr_2$(dme)/dtbbpy + $[Co(CpEt)_2]^+$ + **1a** (green), and $NiBr_2$(dme)/dtbbpy + $[Co(CpEt)_2]^+$ + **1a** + **2a** (gray). Blue trace: 5.0 mM $NiBr_2$(dme)/dtbbpy. Orange trace: 5.0 mM $[Co(CpEt)_2]^+$. Green trace: 5.0 mM $NiBr_2$(dme)/dtbbpy + 5.0 mM $[Co(CpEt)_2]^+$. Gray trace: 5.0 mM $NiBr_2$(dme)/dtbbpy + 5.0 mM $[Co(CpEt)_2]^+$ + 15.0 mM **1a** + 15.0 mM **2a**. All CVs were recorded in NMP (4 mL) with LiBr (0.1 M) as supporting electrolyte, under N_2 atmosphere, at 60 °C, scan rate = 100 mV/s, initially scanning towards a more negative potential.

To more clearly compare changes in the peak heights upon mixing the Ni catalyst and the mediator, the maximum currents of first peak observed at approximately –1.5 V in the Ni-only (blue), mediator-only (orange) and Ni + mediator (green) traces in Figure S13 were recorded and are shown in Table S10 below.

Table S11. Peak heights of Ni-only, mediator-only, and Ni + mediator conditions.

**Condition**	**Peak Height (μA)**
Ni/dtbbpy *(Ni-only)*	–5.61
$[Co(CpEt)_2]^+$ *(mediator-only)*	–4.83
Ni/dtbbpy + $[Co(CpEt)_2]^+$	–9.76

In this case, the peak height of the Ni + mediator trace was slightly lower than the sum of the peak heights of the Ni-only and mediator-only traces. This may indicate that the increase of the peak height of the Ni + mediator condition relative to the Ni-only or mediator-only condition comes from the integration of the Ni-only and mediator-only peaks into one peak.

*6.2 CV studies of Ni/(dtbbpy/ttbtpy) and $Co(CpEt)_2$, with or without substrates **1a** and **2c***

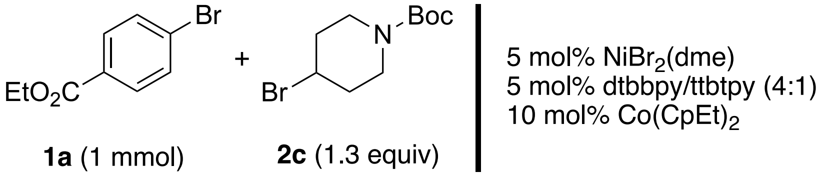

Since CoPc was not included in the previous CV study, that system was not a complete representation of the catalytic system studied in this work (no XAT reagent). To better understand the role of the mediator in an effective catalytic system, CV studies of substrates **1a** and **2c** using Ni/(dtbbpy/ttbtpy) were also conducted.

In a nitrogen-filled glovebox, a stock solution containing 10 mM $NiBr_2$(dme), 8 mM 4,4′-di-*tert*-butyl-2,2′-bipyridine, and 2 mM 4,4′,4″-tri-*tert*-butyl-2,2′:6′,2″-terpyridine in anhydrous NMP (10 mL) was prepared. This solution was stirred at 800 rpm in the glovebox for 3 h to allow complexation of Ni with the ligand. Then LiBr (0.1 M) was added to the solution and stirred until fully dissolved. The resultant solution was used as the stock solution for preparation of any CV solutions containing Ni/(dtbbpy/ttbtpy).

To avoid the chemical reduction of Ni/(dtbbpy/ttbtpy) by $Co(CpEt)_2$, $Co(CpEt)_2$ was oxidized to $[Co(CpEt)_2]^+$ via constant current electrolysis at 5 mA for 64 min (1 F/mol) in a divided cell. A stock solution containing 20 mM $[Co(CpEt)_2]^+$ in NMP (10 mL) with LiBr (0.1 M) was prepared and used for preparation of any CV solutions containing $[Co(CpEt)_2]^+$.

Solutions for all CV studies were prepared according to the description in the caption of each figure. A glassy carbon (GC) working electrode, a platinum counter electrode, and a $Ag/AgNO_3$ reference electrode were used as a three-electrode configuration for all CV experiments (see "Electrochemical experiments" portion in "General Experimental Considerations" for details of the electrodes). A sand bath was used to heat the CV cell and reaction solutions to 60 °C.

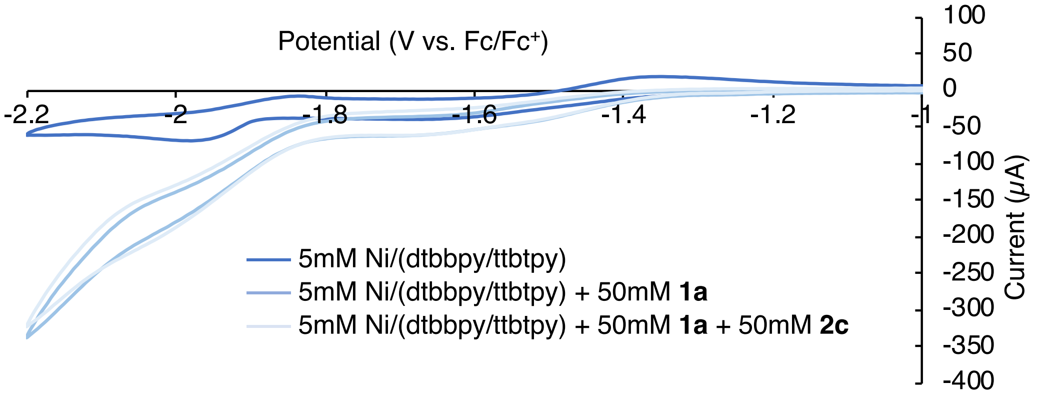

Figure S14. CVs of $NiBr_2$(dme)/(dtbbpy/ttbtpy) only (dark blue), $NiBr_2$(dme)/(dtbbpy/ttbtpy) + **1a** (blue), and $NiBr_2$(dme)/(dtbbpy/ttbtpy) + **1a** + **2c** (light blue). Dark blue trace: 5.0 mM $NiBr_2$(dme)/(dtbbpy/ttbtpy). Blue trace: 5.0 mM $NiBr_2$(dme)/(dtbbpy/ttbtpy) + 50.0 mM **1a**. Light blue trace: 5.0 mM $NiBr_2$(dme)/(dtbbpy/ttbtpy) + 50.0 mM **1a** + 50.0 mM **2c**. All CVs were recorded in NMP (4 mL) with LiBr (0.1 M) as supporting electrolyte, under N_2 atmosphere, at 60 °C, scan rate = 100 mV/s, initially scanning towards a more negative potential.

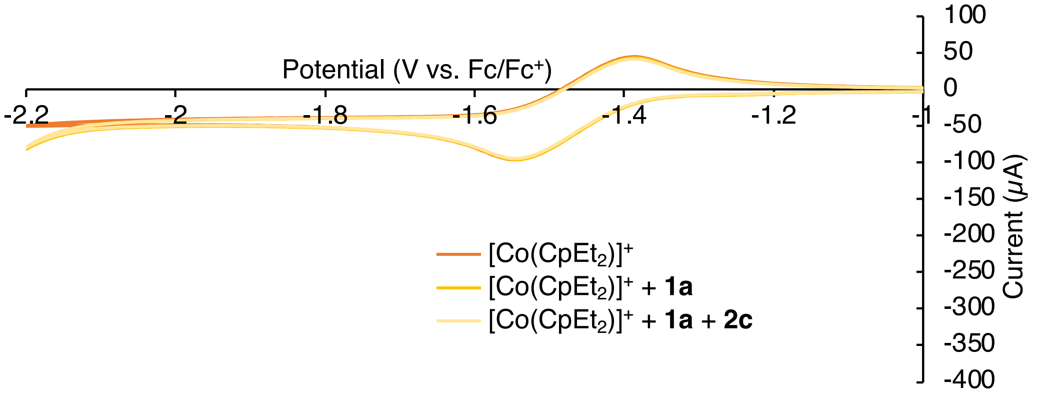

Figure S15. CVs of $[Co(CpEt)_2]^+$ only (orange), $[Co(CpEt)_2]^+$ + **1a** (yellow), and $[Co(CpEt)_2]^+$ + **1a** + **2c** (light yellow). Orange trace: 10.0 mM $[Co(CpEt)_2]^+$. Yellow trace: 10.0 mM $[Co(CpEt)_2]^+$ + 50.0 mM **1a**. Light yellow trace: 10.0 mM $[Co(CpEt)_2]^+$ + 50.0 mM **1a** + 50.0 mM **2c**. All CVs were recorded in NMP (4 mL) with LiBr (0.1 M) as supporting electrolyte, under N_2 atmosphere, at 60 °C, scan rate = 100 mV/s, initially scanning towards a more negative potential.

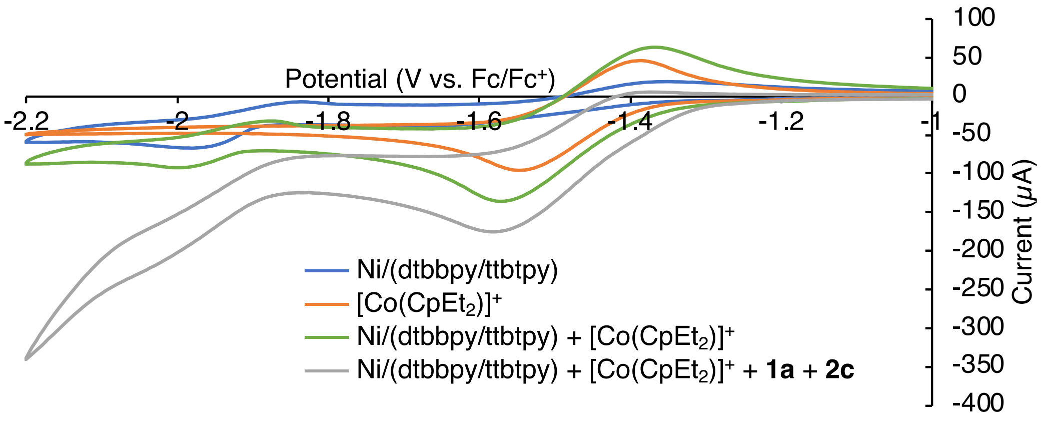

Figure S16. CVs of $NiBr_2$(dme)/(dtbbpy/ttbtpy) only (blue), $[Co(CpEt)_2]^+$ only (orange), $NiBr_2$(dme)/(dtbbpy/ttbtpy) + $[Co(CpEt)_2]^+$ + **1a** (green), and $NiBr_2$(dme)/(dtbbpy/ttbtpy) + $[Co(CpEt)_2]^+$ + **1a** + **2c** (gray). Blue trace: 5.0 mM $NiBr_2$(dme)/(dtbbpy/ttbtpy). Orange trace: 10.0 mM $[Co(CpEt)_2]^+$. Green trace: 5.0 mM $NiBr_2$(dme)/(dtbbpy/ttbtpy) + 10.0 mM $[Co(CpEt)_2]^+$. Gray trace: 5.0 mM $NiBr_2$(dme)/(dtbbpy/ttbtpy) + 10.0 mM $[Co(CpEt)_2]^+$ + 50.0 mM **1a** + 50.0 mM **2c**. All CVs were recorded in NMP (4 mL) with LiBr (0.1 M) as supporting electrolyte, under N_2 atmosphere, at 60 °C, scan rate = 100 mV/s, initially scanning towards a more negative potential.

To more clearly compare changes in the peak heights upon mixing the Ni catalyst and the mediator, the maximum current of the first peak observed at approximately –1.5 V in the Ni-only (blue), mediator-only (orange) and Ni + mediator (green) traces in Figure S16 were recorded and are shown in Table S11 below.

Table S12. Peak heights of Ni-only, mediator-only, and Ni + mediator conditions.

**Condition**	**Peak Height (μA)**
Ni/(dtbbpy/ttbtpy) *(Ni-only)*	–35.73
$[Co(CpEt)_2]^+$ *(mediator-only)*	–90.37
Ni/(dtbbpy/ttbtpy) + $[Co(CpEt)_2]^+$	–131.2

In this case, the peak height of the Ni + mediator trace was greater than the sum of the peak heights of the Ni-only and mediator-only traces, indicating that the increase in the maximum current of the peak observed in the Ni + mediator condition was not merely from the integration of the two individual peaks from the Ni-only and mediator-only condition. This implies that the mediator could be helping catalyst reduction, improving catalyst performance, or both.

6.3 Stability test for $Co(CpEt)_2$

To assess the stability of the optimal ET-mediator, $Co(CpEt)_2$, we attempted to take CVs of the full reaction mixture both before and after the electrolysis. However, the peak arising from the mediator was not identifiable due to its overlap with other peaks from the Ni and Co catalysts. Therefore, a solution containing only 20 mM $Co(CpEt)_2$ in NMP (5 mL) with LiBr (0.1 M) was added to a CV cell, and CV was performed for 1000 cycles between –1.05 V to –1.75 V without stirring at 60 °C. The results showed that there were neither obvious changes in the shape and height of the CV traces nor obvious changes in the i_{pa}/i_{pc} ratios (Figure S15).

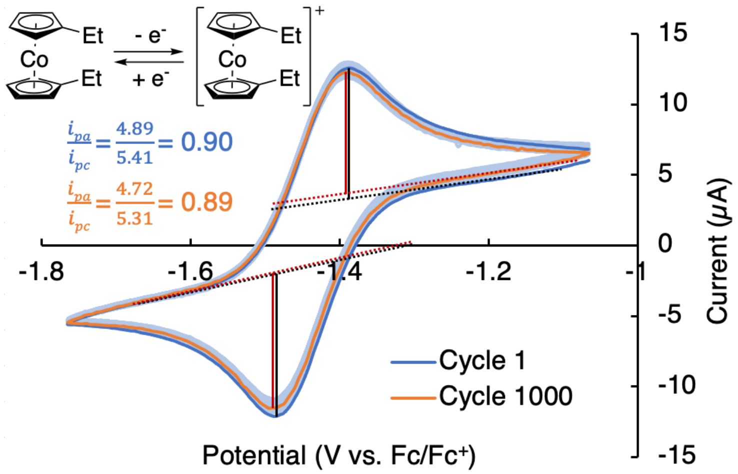

Figure S17. CVs of 1000 cycles of the redox shuttling of $Co(CpEt)_2$. Blue trace: The 1st CV cycle. Orange trace: The 1000th CV cycle. Light blue traces: The 2nd – 999th cycles of the redox shuttling. All CVs were recorded with 20 mM $Co(CpEt)_2$ in NMP (5 mL) with LiBr (0.1 M) as supporting electrolyte, under N_2 atmosphere, at 60 °C, scan rate = 100 mV/s, initially scanning towards a more negative potential.

7. Electrochemically Active Surface Area (ECSA) Measurement of Ni Foam

Although all the current densities reported in this manuscript were based on the geometric surface area of the Ni foam, a measurement of the electrochemically active surface area (ECSA) was conducted by measuring the double-layer capacitance (C_{dl}) of the Ni foam electrode and specific capacitance (C_{sp}) of a Ni rod electrode by CV in a non-Faradaic potential region. A nickel rod (1.6 mm diameter, 6 mm length) with defined surface area was used to derive the value of C_{sp}, and a 1 × 1 cm Ni foam was used for the calculation of C_{dl} and its ECSA (Figure S18).

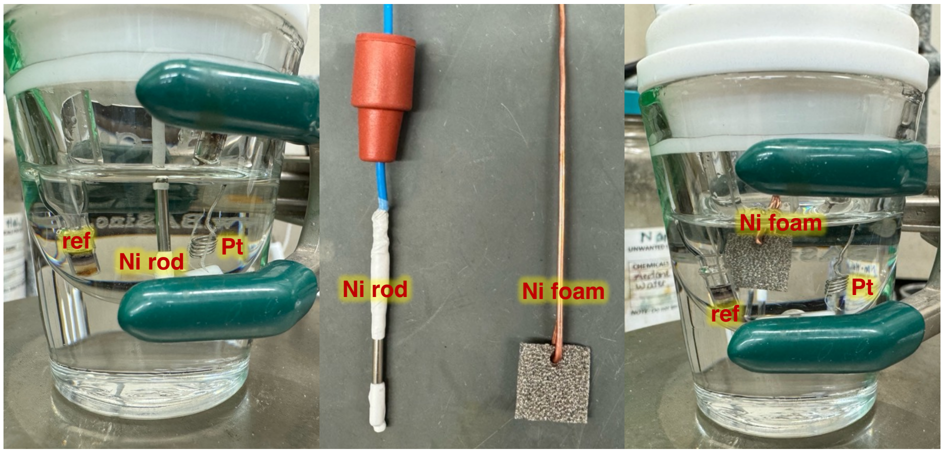

Figure S18. Graphic illustration of the setup of the CV cell for Ni rod (left) or for Ni foam (right), and the close look at both Ni electrodes (middle).

A 10-mL solution containing 0.2 M LiBr in NMP was prepared for the CV experiments. Each electrode was fully immersed in the solution and scanned one CV cycle from –0.9 V to –1.1 V vs Ag/$AgNO_3$. A series of scan rates ranging from 25 to 300 mV/s were used to run the CV experiments (Figure S19), where the maximum currents of the cathodic and anodic waves were obtained from each run (Figure S20).

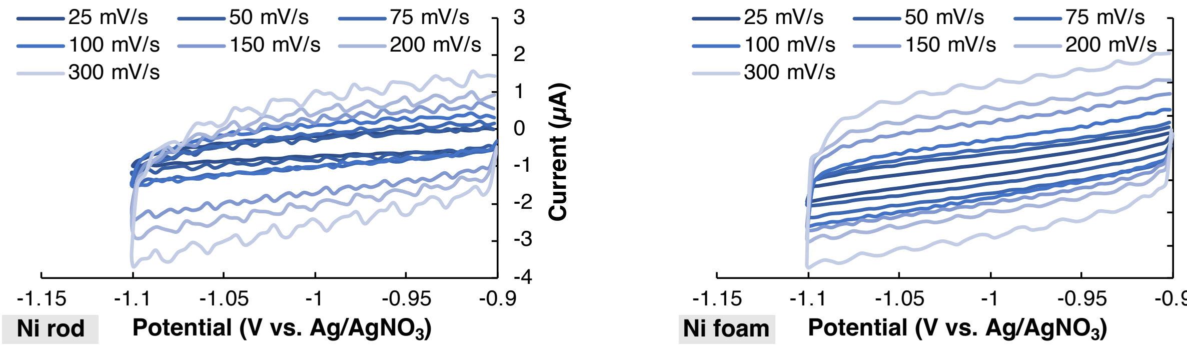

Figure S19. CVs taken with a Ni rod (left) or Ni foam (right) working electrode at different scan rates. All CVs were recorded with 0.2 M LiBr in NMP (10 mL), under N_2 atmosphere, at room temperature with variable scan rates, initially scanning towards a more negative potential.

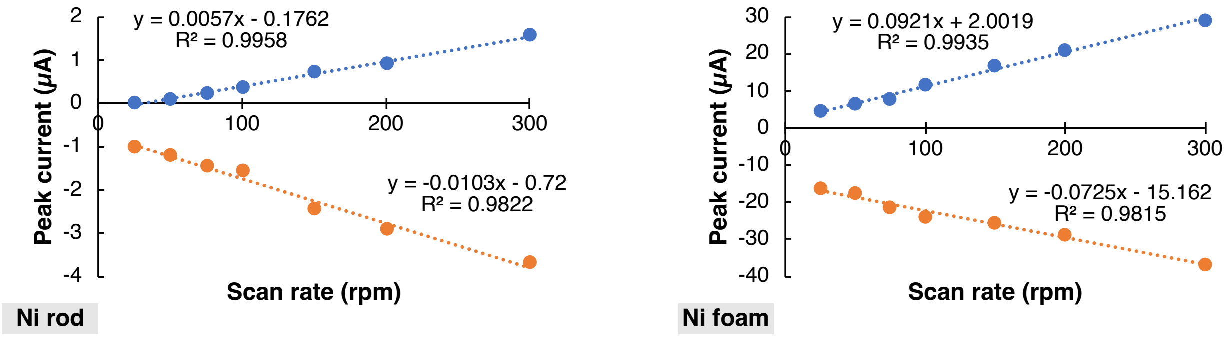

Figure S20. Scan-rate dependance of current of Ni rod (left) or Ni foam (right) working electrode, plotted using anodic (blue) and cathodic (orange) CV maximum currents.

The ECSA of the Ni foam was determined as following:

$$C_{dl,foam} = (|slope_{ox}| + |slope_{red}|) / 2 *1000 = 82.27\ \mu F;$$

$$C_{sp,rod} = C_{dl,rod} / A_{rod} = 26.55\ \mu F/cm^2;$$

$$\mathbf{ECSA = C_{dl,foam}/C_{sp,rod} = 3.10\ cm^2}$$

According to the calculation, the ECSA of a 1 × 1 cm piece of Ni foam (which gives a geometric surface area of 2 cm^2 in batch since both sides of the foam electrode are exposed to solution/active) was 3.10 cm^2, resulting in a 2.6 mA/cm^2 current density for batch reactions of the substrate scope (**Figure 3** of the manuscript) in comparison to 4 mA/cm^2 calculated from the geometric surface area. For flow electrolysis, one side of the 3 × 3 cm Ni foam was blocked, making it difficult to directly calculate the ECSA of the electrode inside the flow reactor.

8. Compound Characterization Data

ethyl 4-(3-phenylpropyl)benzoate (**3a**)

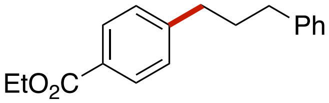

GP 1 was followed using ethyl 4-bromobenzoate (229.1 mg, 1 mmol, 1.0 equiv) and 1-bromo-3-phenylpropane (258.7 mg, 1.3 mmol, 1.3 equiv) as starting materials, $NiBr_2$(dme) (6.2 mg, 2 mol%) complexed with 4,4′-di-*tert*-butyl-2,2′-bipyridine (5.4 mg, 2 mol%), and cobalt phthalocyanine (28.6 mg, 5 mol%) as the catalysts, and bis(ethylcyclopentadienyl)cobalt (24.5 mg, 16.2 µL, 10 mol%) as the mediator. Electrolyte solution (0.2 M LiBr in NMP) was added to each chamber, 5 mL and 8 mL to the cathode and anode, respectively. Constant current electrolysis was performed at 8 mA until the passage of 2.1 F/mol. Upon completion of the reaction the catholyte was collected and extracted with EtOAc from H_2O/NMP, and the organic layer was concentrated to dryness. The resultant residue was purified by silica gel chromatography using hexane/EtOAc (100:0 to 90:10 gradient) to afford the title compound as a colorless oil (254.6 mg, 95% yield). The spectroscopic data matched those reported in the literature.[5]

^{1}H NMR (500 MHz, $CDCl_3$) δ 7.96 (d, J = 8.3 Hz, 2H), 7.33 – 7.22 (m, 5H), 7.19 (ddd, J = 8.1, 7.0, 1.6 Hz, 3H), 4.36 (q, J = 7.1 Hz, 3H), 2.03 – 1.91 (m, 2H), 1.39 (t, J = 7.1 Hz, 4H).

^{13}C NMR (126 MHz, $CDCl_3$) δ 166.8, 147.8, 142.1, 129.8, 128.6, 128.5, 128.3, 126.0, 60.9, 35.6, 35.5, 32.7, 14.5.

4-(3-phenylpropyl)benzonitrile (**3b**)

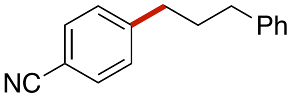

GP 1 was followed using ethyl 4-bromobenzonitrile (182.0 mg, 1 mmol, 1.0 equiv) and 1-bromo-3-phenylpropane (258.7 mg, 1.3 mmol, 1.3 equiv) as starting materials, $NiBr_2$(dme) (6.2 mg, 2 mol%) complexed with 4,4′-di-*tert*-butyl-2,2′-bipyridine (5.4 mg, 2 mol%), and cobalt phthalocyanine (28.6 mg, 5 mol%) as the catalysts, and bis(ethylcyclopentadienyl)cobalt (24.5 mg, 16.2 µL, 10 mol%) as the mediator. Electrolyte solution (0.2 M LiBr in NMP) was added to each chamber, 5 mL and 8 mL to the cathode and anode respectively. Constant current electrolysis was performed at 8 mA until the passage of 2.1 F/mol. Upon completion of the reaction the catholyte was collected and extracted with EtOAc from H_2O/NMP, and the organic layer was concentrated to dryness. The resultant residue was purified by silica gel chromatography using hexane/EtOAc (100:0 to 90:10 gradient) to afford the title compound as a yellow oil (216.6 mg, 98% yield). The spectroscopic data matched those reported in the literature.[6]

^{1}H NMR (500 MHz, $CDCl_3$) δ 7.56 (d, J = 8.2 Hz, 2H), 7.32 – 7.24 (m, 4H), 7.20 (d, J = 7.2 Hz, 1H), 7.19 – 7.13 (m, 2H), 2.70 (d, J = 7.7 Hz, 2H), 2.65 (t, J = 7.6 Hz, 2H), 2.00 – 1.92 (m, 2H).

^{13}C NMR (126 MHz, $CDCl_3$) δ 148.1, 141.7, 132.3, 129.4, 128.6, 128.5, 126.1, 119.2, 109.9, 35.6, 35.4, 32.5.

1-methoxy-4-(3-phenylpropyl)benzene (**3c**)

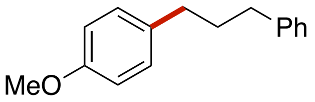

GP 1 was followed using 1-bromo-4-methoxybenzene (187.0 mg, 1 mmol, 1.0 equiv) and 1-bromo-3-phenylpropane (258.7 mg, 1.3 mmol, 1.3 equiv) as starting materials, $NiBr_2$(dme) (3.1 mg, 1 mol%) complexed with 4,4′-di-*tert*-butyl-2,2′-bipyridine (2.7 mg, 1 mol%), and cobalt phthalocyanine (14.3 mg, 2.5 mol%) as the catalysts, and bis(ethylcyclopentadienyl)cobalt (24.5 mg, 16.2 µL, 10 mol%) as the mediator. Electrolyte solution (0.2 M LiBr in NMP) was added to each chamber, 5 mL and 8 mL to the cathode and anode respectively. Constant current electrolysis was performed at 8 mA until the passage of 2.1 F/mol. Upon completion of the reaction the catholyte was collected and extracted with EtOAc from H_2O/NMP, and the organic layer was concentrated to dryness. The resultant residue was purified by silica gel chromatography using hexane/EtOAc (100:0 to 80:20 gradient) to afford the title compound as a colorless oil (171.8 mg, 76% yield). The spectroscopic data matched those reported in the literature.[6]

^{1}H NMR (500 MHz, $CDCl_3$) δ 7.31 – 7.23 (m, 2H), 7.18 (d, *J* = 6.5 Hz, 3H), 7.10 (d, *J* = 8.3 Hz, 2H), 6.86 – 6.80 (m, 2H), 3.78 (s, 3H), 2.64 (t, *J* = 7.8 Hz, 2H), 2.59 (t, *J* = 7.7 Hz, 2H), 1.93 (dt, *J* = 14.6, 7.5 Hz, 2H).

^{13}C NMR (126 MHz, $CDCl_3$) δ 157.9, 142.5, 134.5, 129.4, 128.6, 128.4, 125.8, 113.9, 55.4, 35.5, 34.7, 33.3.

ethyl 3-(3-phenylpropyl)benzoate (**3d**)

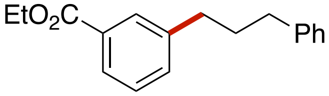

GP 1 was followed using ethyl 3-bromobenzoate (229.1 mg, 1 mmol, 1.0 equiv) and 1-bromo-3-phenylpropane (258.7 mg, 1.3 mmol, 1.3 equiv) as starting materials, $NiBr_2$(dme) (3.1 mg, 1 mol%) complexed with 4,4′-di-*tert*-butyl-2,2′-bipyridine (2.7 mg, 1 mol%), and cobalt phthalocyanine (14.3 mg, 2.5 mol%) as the catalysts, and bis(ethylcyclopentadienyl)cobalt (24.5 mg, 16.2 µL, 10 mol%) as the mediator. Electrolyte solution (0.2 M LiBr in NMP) was added to each chamber, 5 mL and 8 mL to the cathode and anode respectively. Constant current electrolysis was performed at 8 mA until the passage of 2.1 F/mol. Upon completion of the reaction the catholyte was collected and extracted with EtOAc from H_2O/NMP, and the organic layer was concentrated to dryness. The resultant residue was purified by silica gel chromatography using hexane/EtOAc (100:0 to 95:5 gradient) to afford the title compound as a colorless oil (214.4 mg, 80% yield). The spectroscopic data matched those reported in the literature.[7]

^{1}H NMR (500 MHz, $CDCl_3$) δ 7.89 – 7.84 (m, 2H), 7.39 – 7.32 (m, 2H), 7.31 – 7.24 (m, 2H), 7.21 – 7.15 (m, 3H), 4.37 (q, *J* = 7.1 Hz, 2H), 2.70 (d, *J* = 7.5 Hz, 2H), 2.66 (d, *J* = 7.6 Hz, 2H), 1.98 (p, *J* = 7.8 Hz, 2H), 1.40 (t, *J* = 7.1 Hz, 3H).

^{13}C NMR (126 MHz, $CDCl_3$) δ 167.0, 142.7, 142.2, 133.1, 130.7, 129.6, 128.6, 128.5, 128.4, 127.2, 126.0, 61.0, 35.5, 35.3, 33.0, 14.5.

ethyl 2-(3-phenylpropyl)benzoate (**3e**)

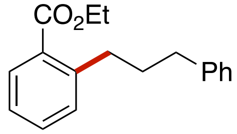

GP 1 was followed using ethyl 2-bromobenzoate (229.1 mg, 1 mmol, 1.0 equiv) and 1-bromo-3-phenylpropane (258.7 mg, 1.3 mmol, 1.3 equiv) as starting materials, $NiBr_2$(dme) (3.1 mg, 1 mol%) complexed with 4,4′-di-*tert*-butyl-2,2′-bipyridine (2.7 mg, 1 mol%), and cobalt phthalocyanine (14.3 mg, 2.5 mol%) as the catalysts, and bis(ethylcyclopentadienyl)cobalt (24.5 mg, 16.2 µL, 10 mol%) as the mediator. Electrolyte solution (0.2 M LiBr in NMP) was added to each chamber, 5 mL and 8 mL to the cathode and anode respectively. Constant current electrolysis was performed at 8 mA until the passage of 2.1 F/mol. Upon completion of the reaction the catholyte was collected and extracted with EtOAc from H_2O/NMP, and the organic layer was concentrated to dryness. The resultant residue was purified by silica gel chromatography using hexane/EtOAc (100:0 to 95:5 gradient) to afford the title compound as a colorless oil (238.5 mg, 89% yield). The spectroscopic data matched those reported in the literature.[5]

^{1}H NMR (500 MHz, $CDCl_3$) δ 7.96 (d, *J* = 8.2 Hz, 2H), 7.31 – 7.26 (m, 2H), 7.24 (d, *J* = 8.2 Hz, 2H), 7.21 – 7.15 (m, 3H), 4.36 (q, *J* = 7.1 Hz, 2H), 2.70 (t, *J* = 7.7 Hz, 2H), 2.65 (t, *J* = 7.7 Hz, 2H), 1.98 (d, *J* = 7.6 Hz, 2H), 1.38 (t, *J* = 7.1 Hz, 3H).

^{13}C NMR (126 MHz, $CDCl_3$) δ 166.8, 147.8, 142.1, 129.8, 128.6, 128.5, 128.3, 126.0, 60.9, 35.6, 35.5, 32.7, 14.5.

7-(3-phenylpropyl)-3,4-dihydronaphthalen-1(2*H*)-one (**3f**)

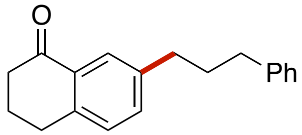

GP 1 was followed using 7-bromo-3,4-dihydronaphthalen-1(2*H*)-one (225.1 mg, 1 mmol, 1.0 equiv) and 1-bromo-3-phenylpropane (258.7 mg, 1.3 mmol, 1.3 equiv) as starting materials, $NiBr_2$(dme) (3.1 mg, 1 mol%) complexed with 4,4′-di-*tert*-butyl-2,2′-bipyridine (2.7 mg, 1 mol%), and cobalt phthalocyanine (14.3 mg, 2.5 mol%) as the catalysts, and bis(ethylcyclopentadienyl)cobalt (24.5 mg, 16.2 µL, 10 mol%) as the mediator. Electrolyte solution (0.2 M LiBr in NMP) was added to each chamber, 5 mL and 8 mL to the cathode and anode respectively. Constant current electrolysis was performed at 8 mA until the passage of 2.1 F/mol. Upon completion of the reaction the catholyte was collected and extracted with EtOAc from H_2O/NMP, and the organic layer was concentrated to dryness. The resultant residue was purified by silica gel chromatography using hexane/EtOAc (100:0 to 95:5 gradient) to afford the title compound as a colorless oil (166.5 mg, 63% yield).

^{1}H NMR (500 MHz, $CDCl_3$) δ 7.87 (s, 1H), 7.32 – 7.23 (m, 3H), 7.21 – 7.12 (m, 4H), 2.93 (t, *J* = 6.1 Hz, 2H), 2.69 – 2.60 (m, 6H), 2.12 (p, *J* = 6.4 Hz, 2H), 1.95 (pt, *J* = 9.3, 6.9 Hz, 2H).

^{13}C NMR (126 MHz, $CDCl_3$) δ 198.7, 142.1, 140.9, 133.9, 132.6, 128.9, 128.5, 128.5, 128.4, 126.8, 125.9, 39.3, 35.5, 35.0, 32.9, 29.5, 23.5.

HRMS (ESI$^+$) Calc: $[M+H]^+$ ($C_{19}H_{21}O$) 265.1587; measured: 265.1584 = 1.1 ppm difference.

1-methyl-5-(3-phenylpropyl)-1*H*-indole (**3g**)

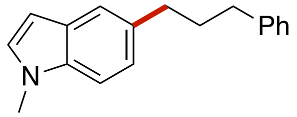

GP 1 was followed using 5-bromo-1-methyl-1*H*-indole (210.1 mg, 1 mmol, 1.0 equiv) and 1-bromo-3-phenylpropane (258.7 mg, 1.3 mmol, 1.3 equiv) as starting materials, $NiBr_2$(dme) (3.1 mg, 1 mol%) complexed with 4,4′-di-*tert*-butyl-2,2′-bipyridine (2.7 mg, 1 mol%), and cobalt phthalocyanine (14.3 mg, 2.5 mol%) as the catalysts, and bis(ethylcyclopentadienyl)cobalt (24.5 mg, 16.2 µL, 10 mol%) as the mediator. Electrolyte solution (0.2 M LiBr in NMP) was added to each chamber, 5 mL and 8 mL to the cathode and anode respectively. Constant current electrolysis was performed at 8 mA until the passage of 2.1 F/mol. Upon completion of the reaction the catholyte was collected and extracted with EtOAc from H_2O/NMP, and the organic layer was concentrated to dryness. The resultant residue was purified by silica gel chromatography using hexane/EtOAc (100:0 to 95:5 gradient) to afford the title compound as a colorless oil (199.2 mg, 80% yield).

^{1}H NMR (500 MHz, $CDCl_3$) δ 7.44 – 7.40 (m, 1H), 7.25 (dd, *J* = 15.1, 7.9 Hz, 3H), 7.21 – 7.14 (m, 3H), 7.06 (dd, *J* = 8.4, 1.7 Hz, 1H), 7.00 (d, *J* = 3.1 Hz, 1H), 6.41 (dd, *J* = 3.1, 0.9 Hz, 1H), 3.76 (s, 3H), 2.75 (t, *J* = 7.6 Hz, 2H), 2.67 (d, *J* = 7.9 Hz, 2H), 2.01 (p, *J* = 7.8 Hz, 2H).

^{13}C NMR (126 MHz, $CDCl_3$) δ 142.8, 135.5, 133.2, 129.0, 128.8, 128.6, 128.4, 125.7, 122.7, 120.2, 109.1, 100.6, 35.7, 35.6, 33.9, 33.0.

HRMS (ESI$^+$) Calc: $[M+H]^+$ ($C_{18}H_{20}N$) 250.1590; measured: 250.1587 = 1.2 ppm difference.

1-(6-(3-phenylpropyl)-3,4-dihydroquinolin-1(2*H*)-yl)ethan-1-one (**3h**)

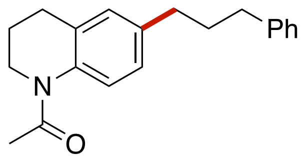

GP 1 was followed using 1-(6-bromo-3,4-dihydroquinolin-1(2*H*)-yl)ethan-1-one (254.1 mg, 1 mmol, 1.0 equiv) and 1-bromo-3-phenylpropane (258.7 mg, 1.3 mmol, 1.3 equiv) as starting materials, $NiBr_2$(dme) (3.1 mg, 1 mol%) complexed with 4,4′-di-*tert*-butyl-2,2′-bipyridine (2.7 mg, 1 mol%), and cobalt phthalocyanine (14.3 mg, 2.5 mol%) as the catalysts, and bis(ethylcyclopentadienyl)cobalt (24.5 mg, 16.2 µL, 10 mol%) as the mediator. Electrolyte solution (0.2 M LiBr in NMP) was added to each chamber, 5 mL and 8 mL to the cathode and anode respectively. Constant current electrolysis was performed at 8 mA until the passage of 2.1 F/mol. Upon completion of the reaction the catholyte was collected and extracted with EtOAc from H_2O/NMP, and the organic layer was concentrated to dryness. The resultant residue was purified by silica gel chromatography using hexane/EtOAc (100:0 to 75:25 gradient) to afford the title compound as a pale yellow oil (223.0 mg, 76% yield).

^{1}H NMR (500 MHz, $CDCl_3$) δ 7.31 – 7.24 (m, 2H), 7.22 – 7.15 (m, 3H), 7.02 – 6.93 (m, 3H), 3.78 (t, *J* = 7.3 Hz, 2H), 2.76 – 2.64 (m, 4H), 2.61 (t, *J* = 7.8 Hz, 2H), 2.22 (s, 3H), 2.03 – 1.86 (m, 4H).

^{13}C NMR (126 MHz, $CDCl_3$) δ 170.2, 142.3, 128.6 (3C), 128.5 (4C), 126.2, 125.9, 124.5, 35.6, 35.0, 33.0, 27.1, 24.2, 23.3.

HRMS (ESI$^+$) Calc: $[M+H]^+$ ($C_{20}H_{24}NO$) 294.1852; measured: 294.1849 = 1.0 ppm difference.

2-(3-phenylpropyl)-4-(trifluoromethyl)pyridine (**3i**)

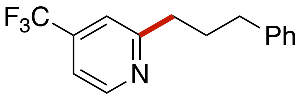

GP 1 was followed using 2-bromo-4-(trifluoromethyl)pyridine (226.0 mg, 1 mmol, 1.0 equiv) and 1-bromo-3-phenylpropane (258.7 mg, 1.3 mmol, 1.3 equiv) as starting materials, $NiBr_2$(dme) (3.1 mg, 1 mol%) complexed with 4,4′-di-*tert*-butyl-2,2′-bipyridine (2.7 mg, 1 mol%), and cobalt phthalocyanine (14.3 mg, 2.5 mol%) as the catalysts, and bis(ethylcyclopentadienyl)cobalt (24.5 mg, 16.2 μL, 10 mol%) as the mediator. Electrolyte solution (0.2 M LiBr in NMP) was added to each chamber, 5 mL and 8 mL to the cathode and anode respectively. Constant current electrolysis was performed at 8 mA until the passage of 2.1 F/mol. Upon completion of the reaction the catholyte was collected and extracted with EtOAc from H_2O/NMP, and the organic layer was concentrated to dryness. The resultant residue was purified by silica gel chromatography using hexane/EtOAc (100:0 to 90:10 gradient) to afford the title compound as a pale yellow oil (140.6 mg, 53% yield).

^{1}H NMR (500 MHz, $CDCl_3$) δ 8.71 (d, J = 5.0 Hz, 1H), 7.35 – 7.31 (m, 2H), 7.31 – 7.24 (m, 2H), 7.22 – 7.16 (m, 3H), 2.91 (t, J = 7.5 Hz, 2H), 2.70 (t, J = 7.7 Hz, 2H), 2.11 (pt, J = 7.8, 2.2 Hz, 2H).

^{13}C NMR (126 MHz, $CDCl_3$) δ 163.8, 150.4, 141.8, 138.8 (q, J = 33.7 Hz), 128.6, 128.5, 126.1, 123.1 (q, J = 273.2 Hz), 118.5 (q, J = 3.6 Hz), 116.8 (q, J = 3.5 Hz), 38.0, 35.6, 31.2.

^{19}F NMR (377 MHz, $CDCl_3$) δ –64.8.

HRMS (ESI$^+$) Calc: $[M+H]^+$ ($C_{15}H_{15}F_3N$) 266.1151; measured: 266.1148 = 1.1 ppm difference.

ethyl 4-(4-ethoxy-4-oxobutyl)benzoate (**3j**)

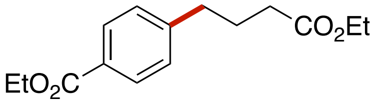

GP 1 was followed using ethyl 4-bromobenzoate (229.1 mg, 1 mmol, 1.0 equiv) and ethyl 4-bromobutanoate (253.6 mg, 1.3 mmol, 1.3 equiv) as starting materials, $NiBr_2$(dme) (3.1 mg, 1 mol%) complexed with 4,4′-di-*tert*-butyl-2,2′-bipyridine (2.7 mg, 1 mol%), and cobalt phthalocyanine (14.3 mg, 2.5 mol%) as the catalysts, and bis(ethylcyclopentadienyl)cobalt (24.5 mg, 16.2 μL, 10 mol%) as the mediator. Electrolyte solution (0.2 M LiBr in NMP) was added to each chamber, 5 mL and 8 mL to the cathode and anode respectively. Constant current electrolysis was performed at 8 mA until the passage of 2.1 F/mol. Upon completion of the reaction the catholyte was collected and extracted with EtOAc from H_2O/NMP, and the organic layer was concentrated to dryness. The resultant residue was purified by silica gel chromatography using

hexane/EtOAc (100:0 to 95:5 gradient) to afford the title compound as a colorless oil (187.4 mg, 71% yield). The spectroscopic data matched those reported in the literature.[5]

^{1}H NMR (500 MHz, $CDCl_3$) δ 7.96 (d, *J* = 8.3 Hz, 2H), 7.24 (d, *J* = 8.3 Hz, 2H), 4.36 (q, *J* = 7.1 Hz, 2H), 4.13 (q, *J* = 7.1 Hz, 2H), 2.71 (t, *J* = 7.8 Hz, 2H), 2.31 (t, *J* = 7.4 Hz, 2H), 1.98 (p, *J* = 7.7 Hz, 2H), 1.39 (t, *J* = 7.1 Hz, 3H), 1.25 (t, *J* = 7.2 Hz, 3H).

^{13}C NMR (126 MHz, $CDCl_3$) δ 173.4, 166.8, 147.0, 129.9, 128.6, 128.6, 60.9, 60.5, 35.3, 33.7, 26.3, 14.5, 14.4.

tert-butyl 4-(4-(ethoxycarbonyl)phenyl)piperidine-1-carboxylate (**3k**)

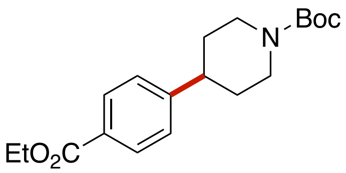

GP 1-2 was followed using ethyl 4-bromobenzoate (229.1 mg, 1 mmol, 1.0 equiv) and *tert*-butyl 4-bromopiperidine-1-carboxylate (343.4 mg, 1.3 mmol, 1.3 equiv) as starting materials, $NiBr_2$(dme) (15.5 mg, 5 mol%) complexed with 4,4′-di-*tert*-butyl-2,2′-bipyridine (10.7 mg, 4 mol%) and 4,4′,4″-tri-tert-butyl-2,2′:6′,2″-terpyridine (4.0 mg, 1 mol%) as the catalysts, and bis(ethylcyclopentadienyl)cobalt (24.5 mg, 16.2 µL, 10 mol%) as the mediator. Electrolyte solution (0.2 M LiBr in NMP) was added to each chamber, 5 mL and 8 mL to the cathode and anode respectively. Constant current electrolysis was performed at 8 mA until the passage of 2.1 F/mol. Upon completion of the reaction the catholyte was collected and extracted with EtOAc from H_2O/NMP, and the organic layer was concentrated to dryness. The resultant residue was purified by silica gel chromatography using hexane/EtOAc (100:0 to 95:5 gradient) to afford the title compound as a pale yellow oil (310.1 mg, 93% yield). The spectroscopic data matched those reported in the literature.[3]

^{1}H NMR (500 MHz, $CDCl_3$) δ 7.98 (d, *J* = 8.4 Hz, 2H), 7.27 (d, *J* = 8.3 Hz, 2H), 4.36 (q, *J* = 7.1 Hz, 2H), 4.26 (s, 2H), 2.81 (t, *J* = 13.0 Hz, 2H), 2.71 (tt, *J* = 12.2, 3.6 Hz, 1H), 1.86 – 1.79 (m, 2H), 1.63 (qd, *J* = 12.5, 4.3 Hz, 2H), 1.49 (s, 9H), 1.38 (t, *J* = 7.1 Hz, 3H).

^{13}C NMR (126 MHz, $CDCl_3$) δ 166.5, 154.8, 151.0, 129.9, 128.8, 126.8, 79.5, 60.8, 42.8, 33.0, 28.5, 14.4.

ethyl 4-benzylbenzoate (**3l**)

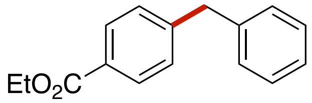

GP 1-3 was followed using ethyl 4-bromobenzoate (229.1 mg, 1 mmol, 1.0 equiv) and *tert*-butyl 4-bromopiperidine-1-carboxylate (343.4 mg, 1.3 mmol, 1.3 equiv) as starting materials, $NiBr_2$(dme) (15.5 mg, 5 mol%) complexed with 4,4′-di-*tert*-butyl-2,2′-bipyridine (13.4 mg, 5 mol%) as the catalysts, and bis(ethylcyclopentadienyl)cobalt (24.5 mg, 16.2 µL, 10 mol%) as the mediator. Electrolyte solution (0.2 M LiBr in NMP) was added to each chamber, 5 mL and 8 mL to the cathode and anode respectively. Constant current electrolysis was performed at 8 mA until

the passage of 2.1 F/mol. Upon completion of the reaction the catholyte was collected and extracted with EtOAc from H_2O/NMP, and the organic layer was concentrated to dryness. The resultant residue was purified by silica gel chromatography using hexane/EtOAc (100:0 to 95:5 gradient) to afford the title compound as a pale yellow oil (199.4 mg, 83% yield). The spectroscopic data matched those reported in the literature.[8]

^{1}H NMR (500 MHz, $CDCl_3$) δ 7.96 (d, J = 8.3 Hz, 2H), 7.32 – 7.19 (m, 5H), 7.17 (d, J = 6.7 Hz, 2H), 4.36 (q, J = 7.1 Hz, 2H), 4.03 (s, 2H), 1.37 (s, 3H).

^{13}C NMR (126 MHz, $CDCl_3$) δ 166.7, 146.5, 140.3, 129.9, 129.1, 129.1, 128.7, 128.6, 126.5, 61.0, 42.1, 14.5.

9. References

(1) Truesdell, B. L.; Hamby, T. B.; Sevov, C. S. General C(sp^2)–C(sp^3) Cross-Electrophile Coupling Reactions Enabled by Overcharge Protection of Homogeneous Electrocatalysts. *J. Am. Chem. Soc.* **2020**, *142* (12), 5884–5893. https://doi.org/10.1021/jacs.0c01475.

(2) Anka-Lufford, L. L.; Huihui, K. M. M.; Gower, N. J.; Ackerman, L. K. G.; Weix, D. J. Nickel-Catalyzed Cross-Electrophile Coupling with Organic Reductants in Non-Amide Solvents. *Chem. – Eur. J.* **2016**, *22* (33), 11564–11567. https://doi.org/10.1002/chem.201602668.

(3) Perkins, R. J.; Hughes, A. J.; Weix, D. J.; Hansen, E. C. Metal-Reductant-Free Electrochemical Nickel-Catalyzed Couplings of Aryl and Alkyl Bromides in Acetonitrile. *Org. Process Res. Dev.* **2019**, *23* (8), 1746–1751. https://doi.org/10.1021/acs.oprd.9b00232.

(4) Twilton, J.; Johnson, M. R.; Sidana, V.; Franke, M. C.; Bottecchia, C.; Lehnherr, D.; Lévesque, F.; Knapp, S. M. M.; Wang, L.; Gerken, J. B.; Hong, C. M.; Vickery, T. P.; Weisel, M. D.; Strotman, N. A.; Weix, D. J.; Root, T. W.; Stahl, S. S. Quinone-Mediated Hydrogen Anode for Non-Aqueous Reductive Electrosynthesis. *Nature* **2023**, *623* (7985), 71–76. https://doi.org/10.1038/s41586-023-06534-2.

(5) Hansen, E. C.; Pedro, D. J.; Wotal, A. C.; Gower, N. J.; Nelson, J. D.; Caron, S.; Weix, D. J. New Ligands for Nickel Catalysis from Diverse Pharmaceutical Heterocycle Libraries. *Nat. Chem.* **2016**, *8* (12), 1126–1130. https://doi.org/10.1038/nchem.2587.

(6) Charboneau, D. J.; Barth, E. L.; Hazari, N.; Uehling, M. R.; Zultanski, S. L. A Widely Applicable Dual Catalytic System for Cross-Electrophile Coupling Enabled by Mechanistic Studies. *ACS Catal.* **2020**, *10* (21), 12642–12656. https://doi.org/10.1021/acscatal.0c03237.

(7) Gao, N.; Li, Y.; Teng, D. Nickel-Catalysed Cross-Electrophile Coupling of Aryl Bromides and Primary Alkyl Bromides. *RSC Adv.* **2022**, *12* (6), 3569–3572. https://doi.org/10.1039/D2RA00010E.

(8) Cao, Q.; Howard, J. L.; Wheatley, E.; Browne, D. L. Mechanochemical Activation of Zinc and Application to Negishi Cross-Coupling. *Angew. Chem. Int. Ed.* **2018**, *57* (35), 11339–11343. https://doi.org/10.1002/anie.201806480.

10. NMR Spectra of Compounds

^{1}H NMR (500 MHz, $CDCl_3$) of **3a**

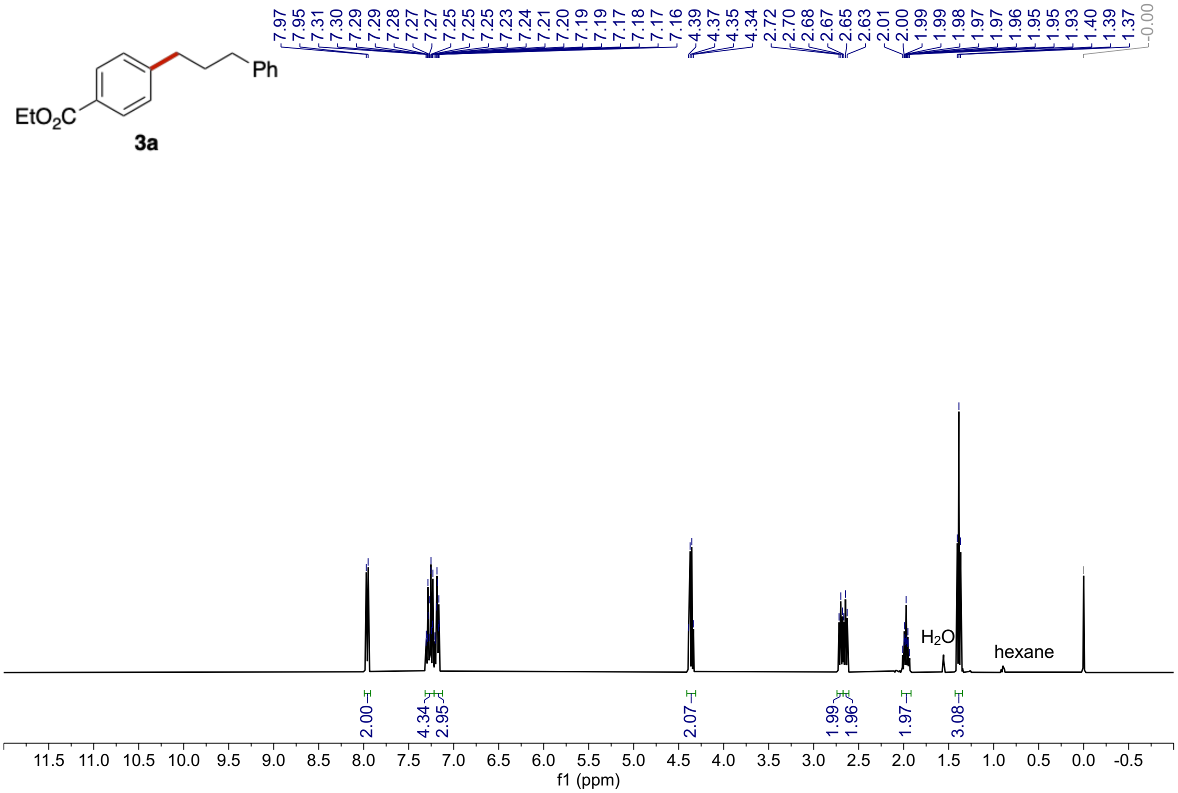

^{13}C NMR (500 MHz, $CDCl_3$) of **3a**

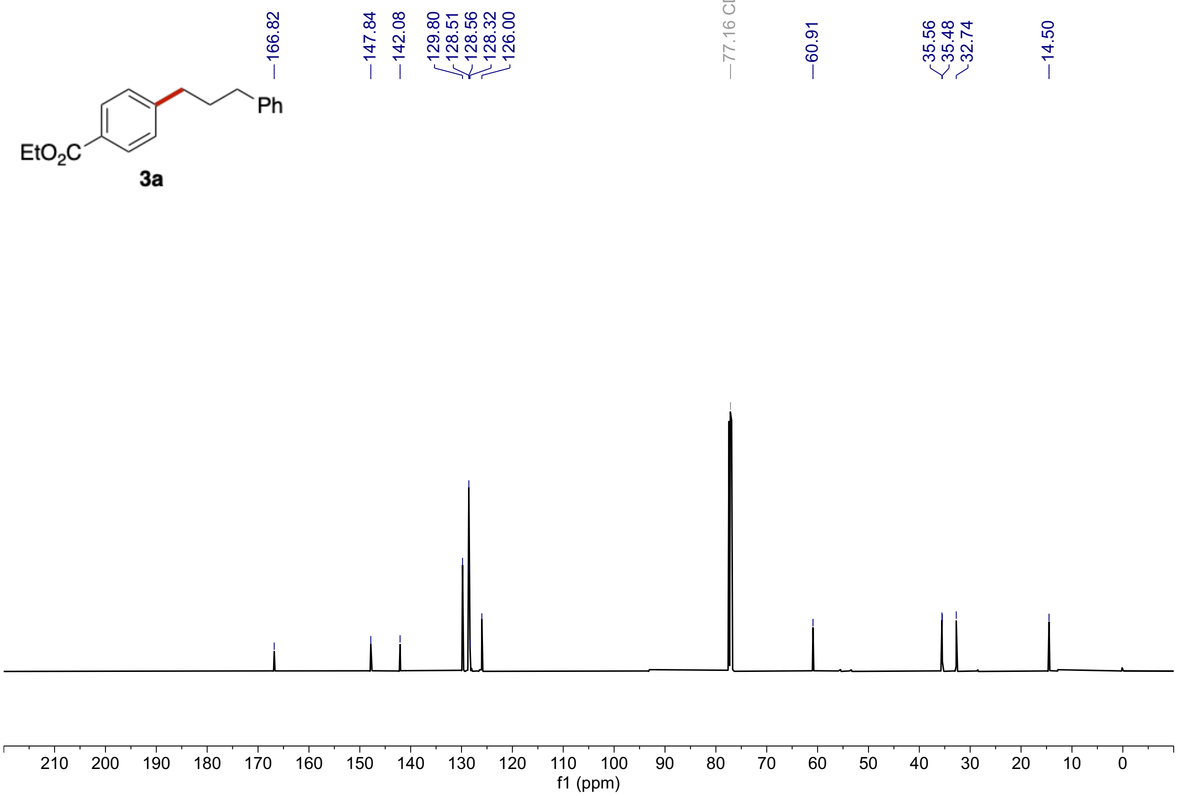

^{1}H NMR (500 MHz, $CDCl_3$) of **3b**

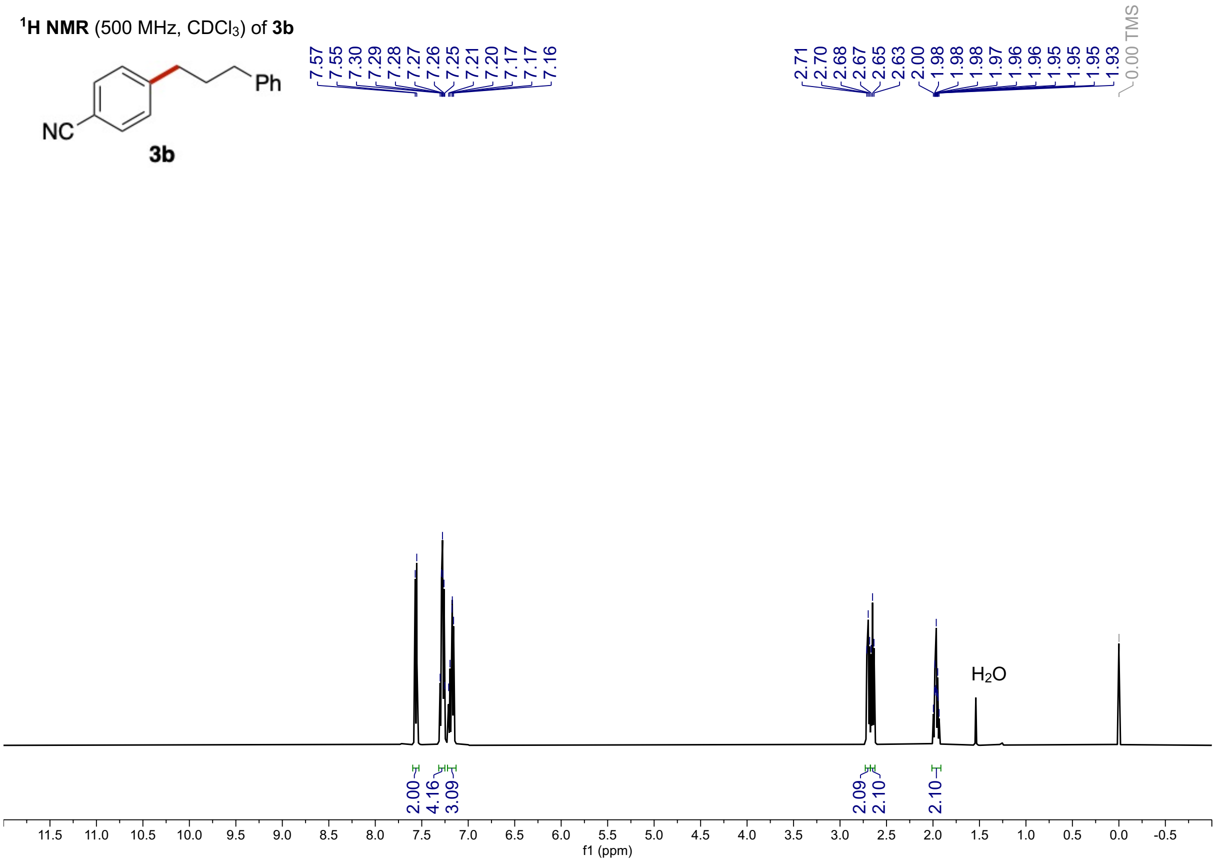

^{13}C NMR (500 MHz, $CDCl_3$) of **3b**

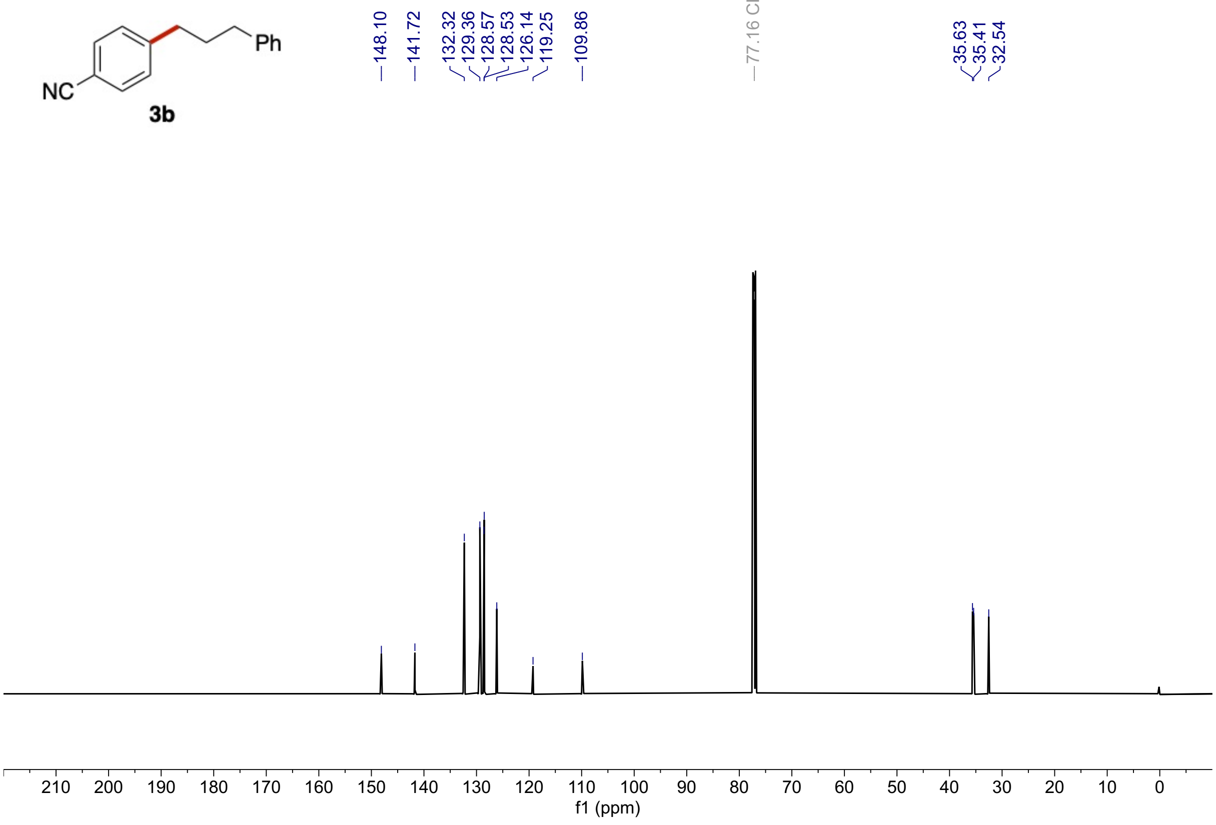

^{1}H NMR (500 MHz, $CDCl_3$) of **3c**

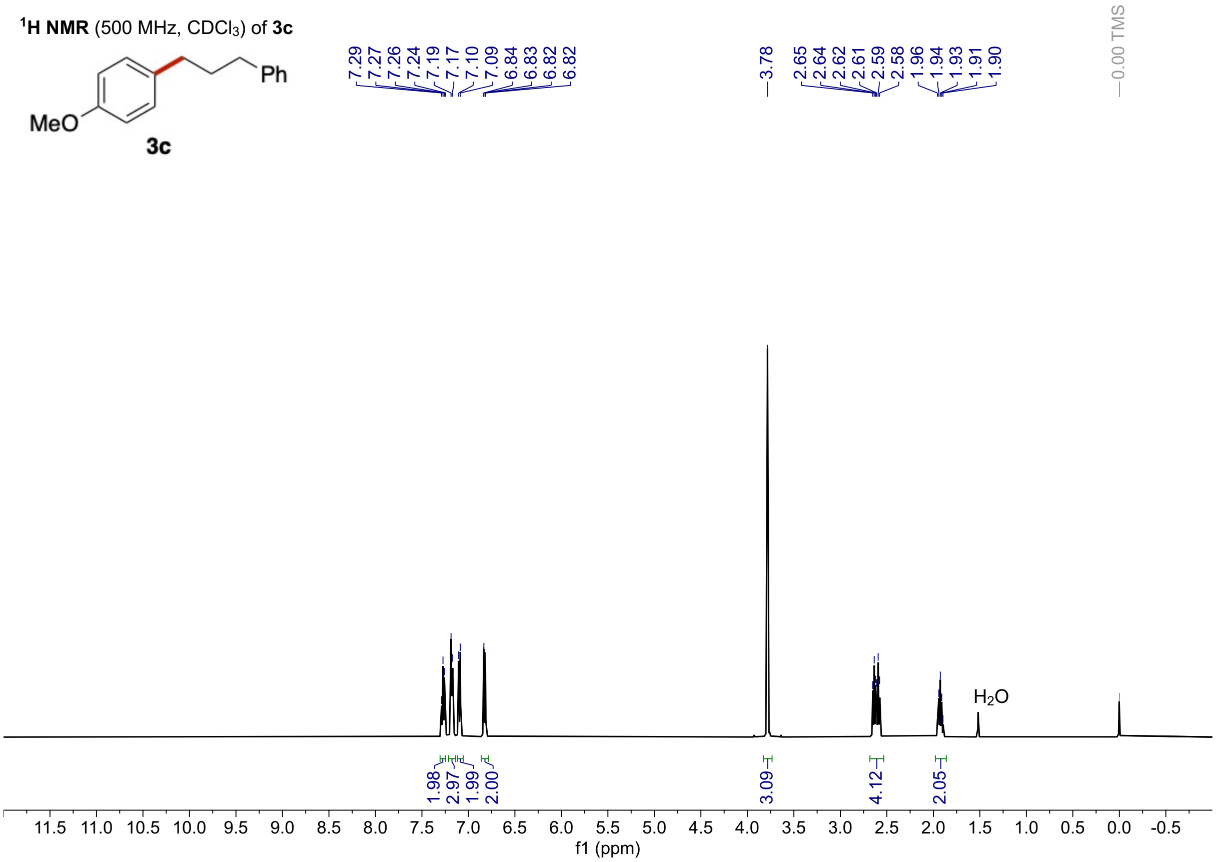

^{13}C NMR (500 MHz, $CDCl_3$) of **3c**

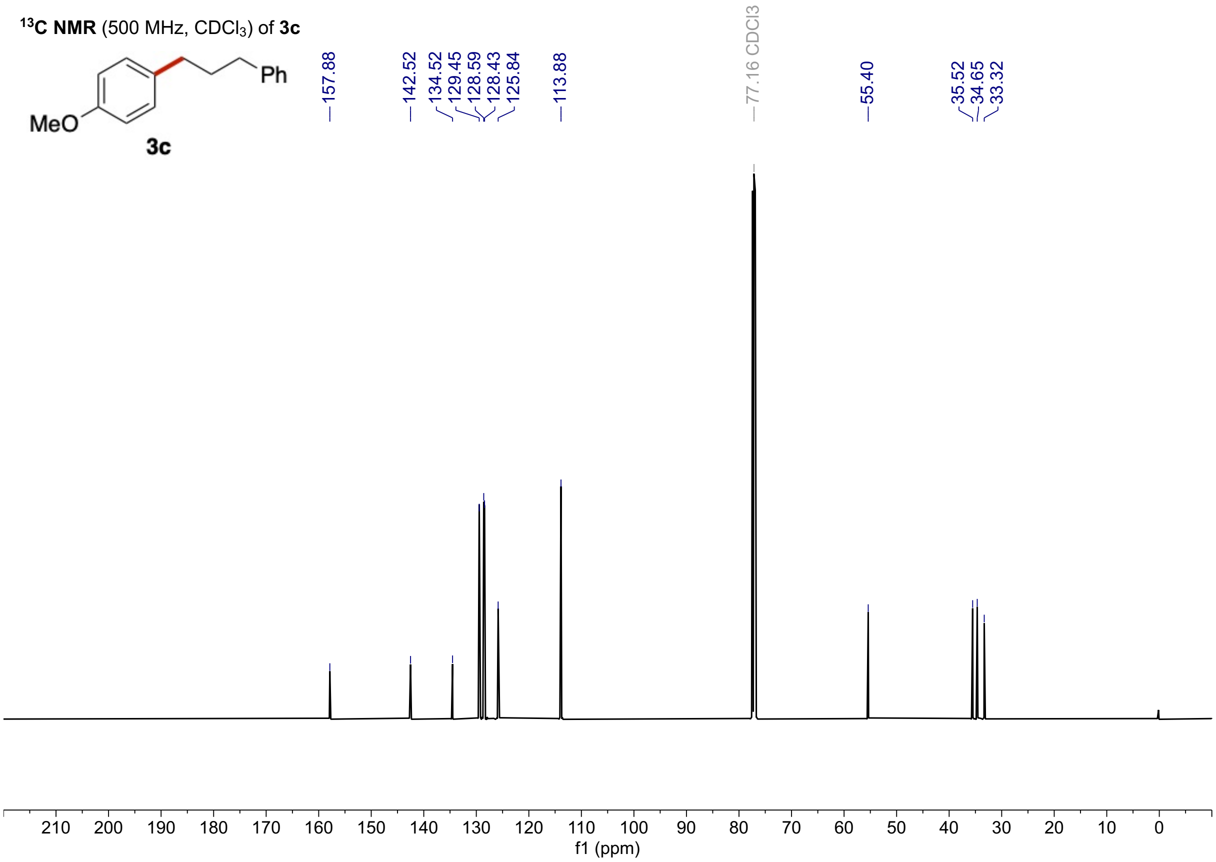

^{1}H NMR (500 MHz, $CDCl_3$) of **3d**

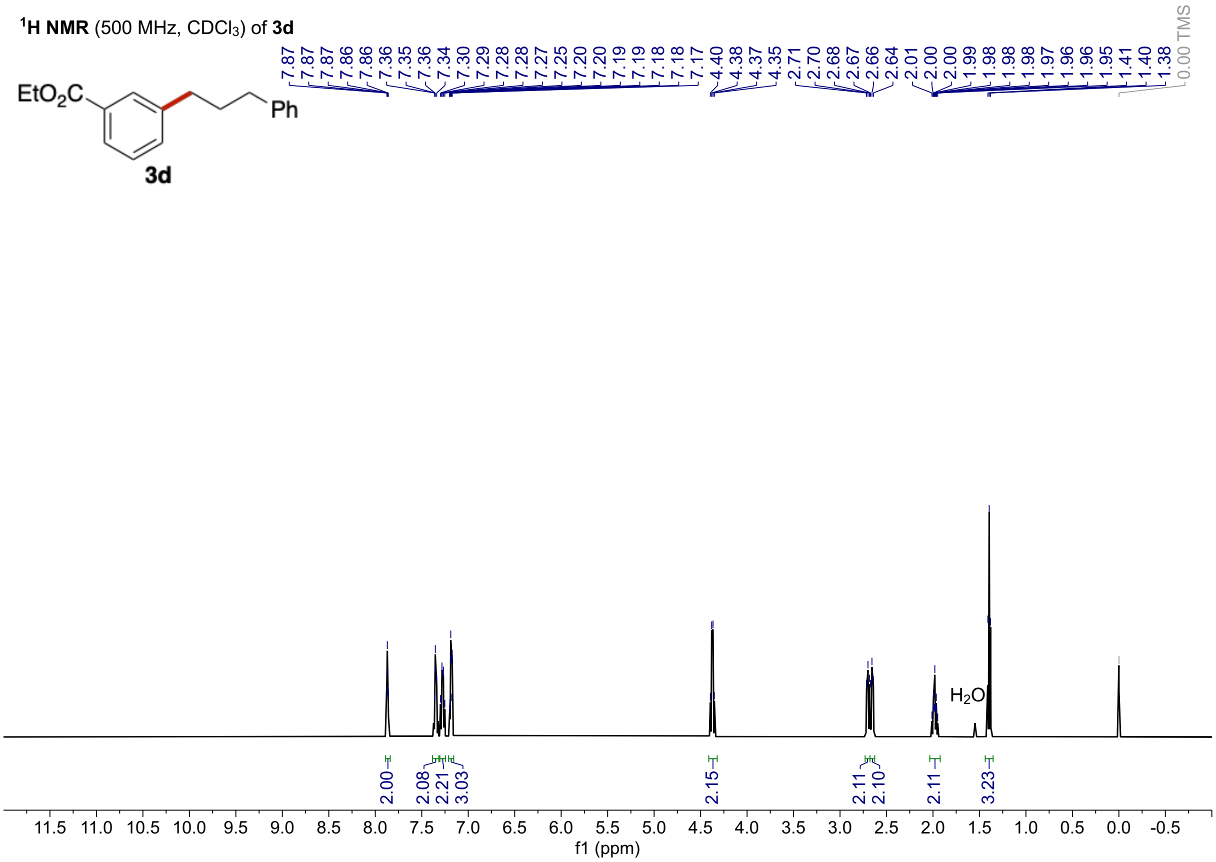

^{13}C NMR (500 MHz, $CDCl_3$) of **3d**

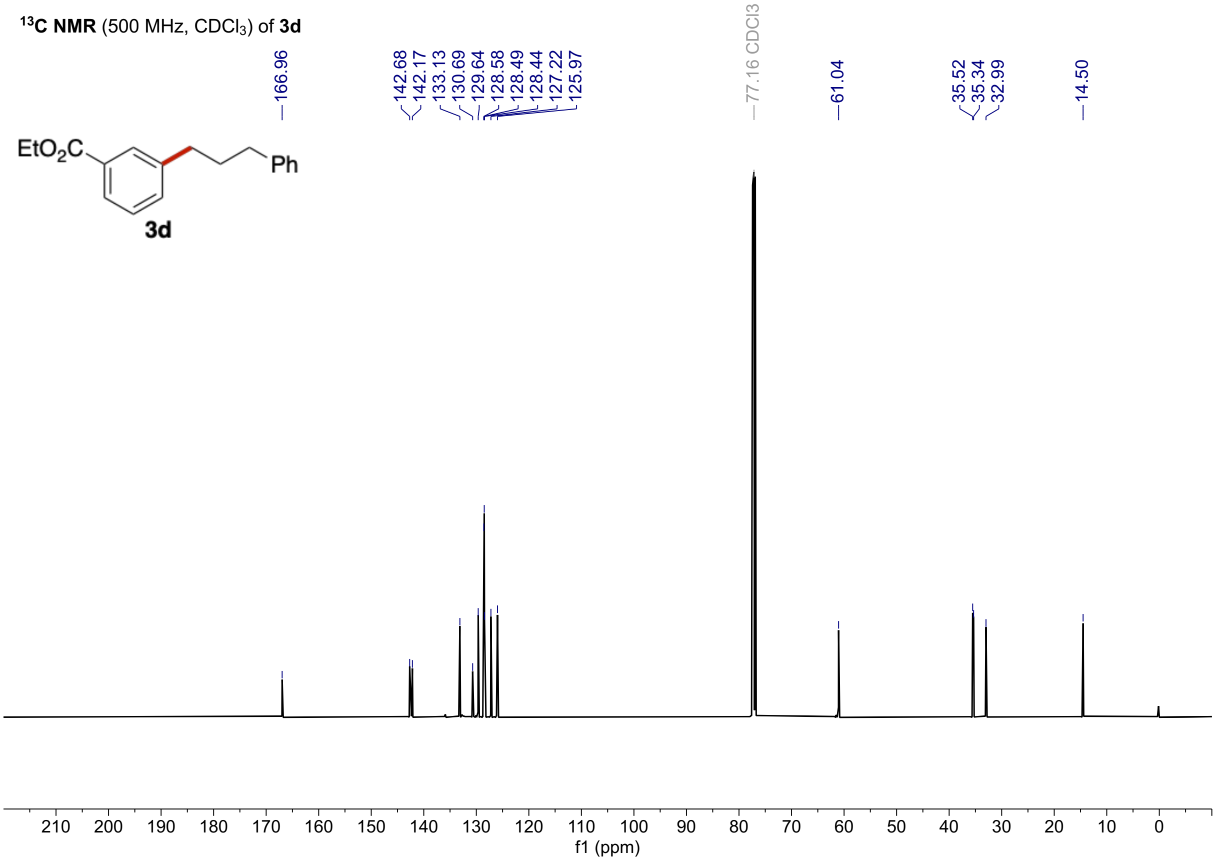

^{1}H NMR (500 MHz, $CDCl_3$) of **3e**

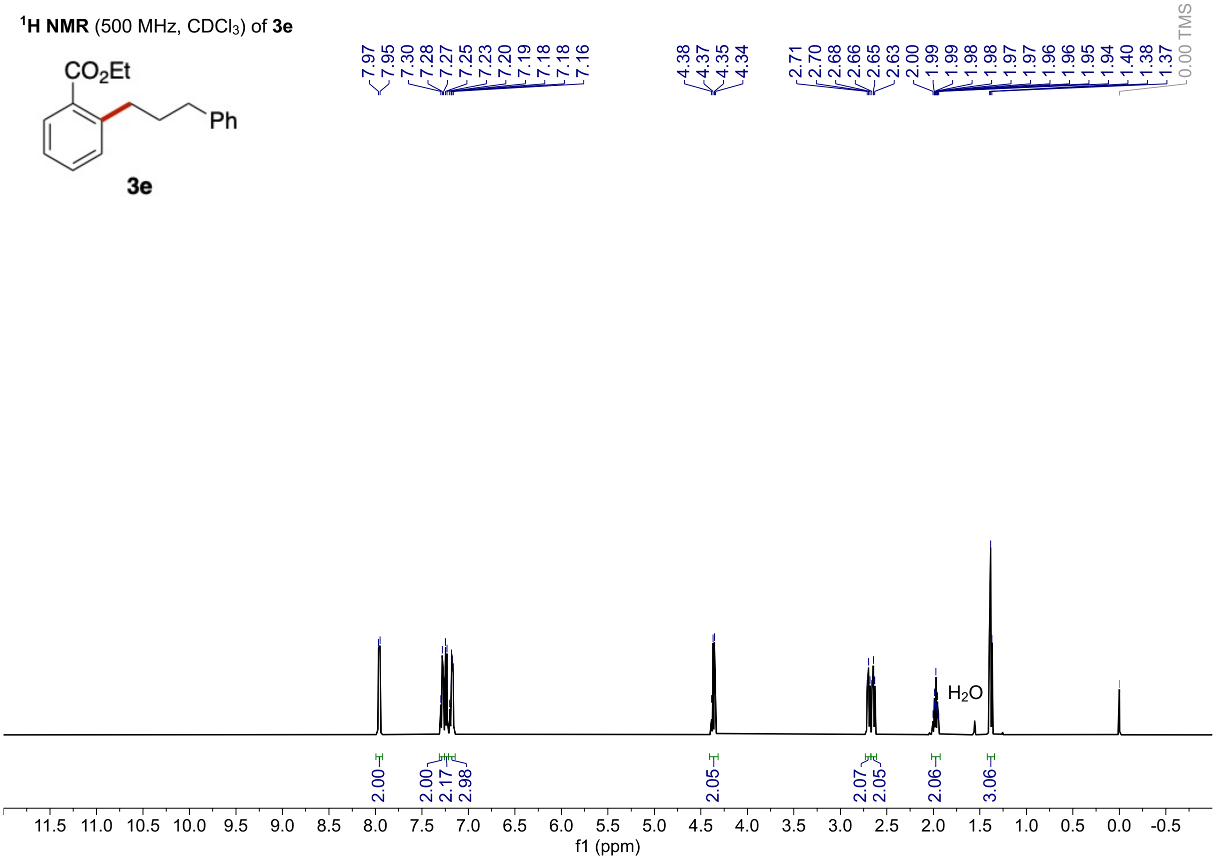

^{13}C NMR (500 MHz, $CDCl_3$) of **3e**

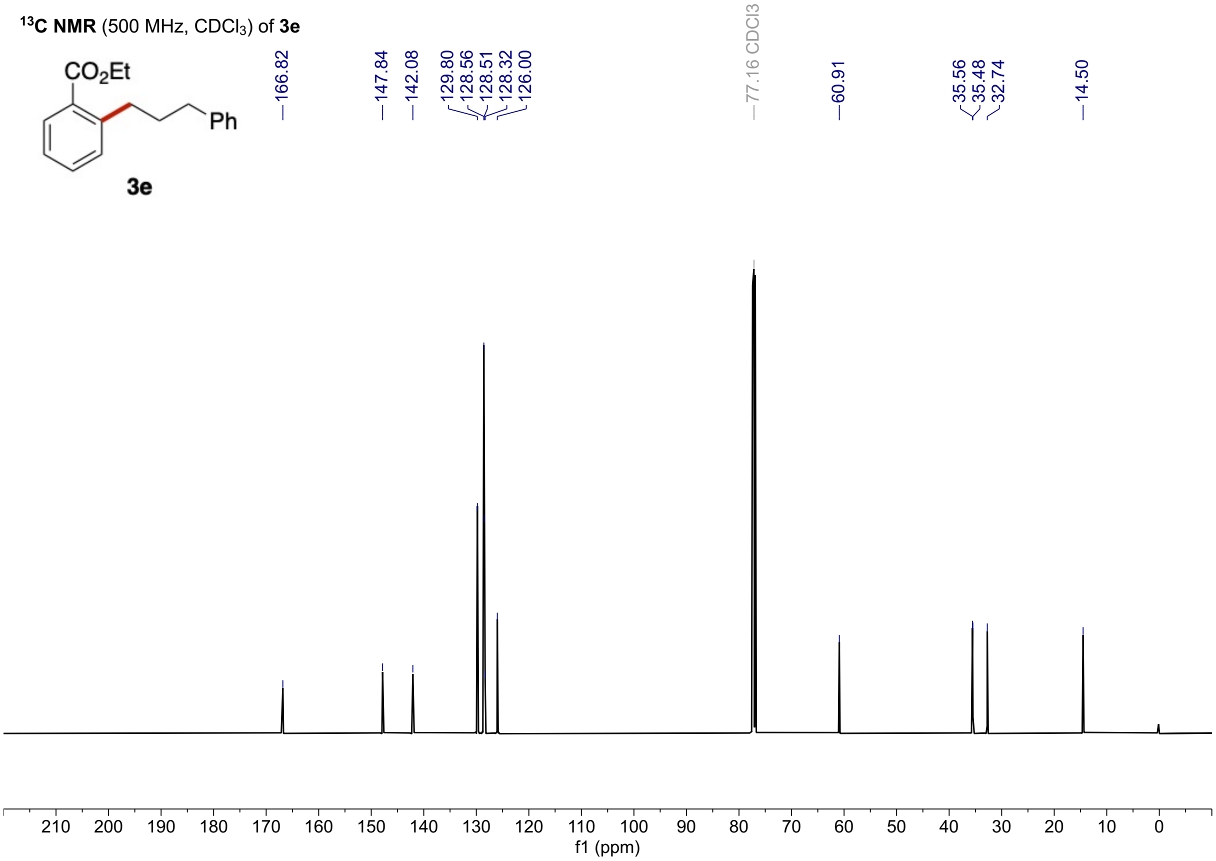

^{1}H NMR (500 MHz, $CDCl_3$) of **3f**

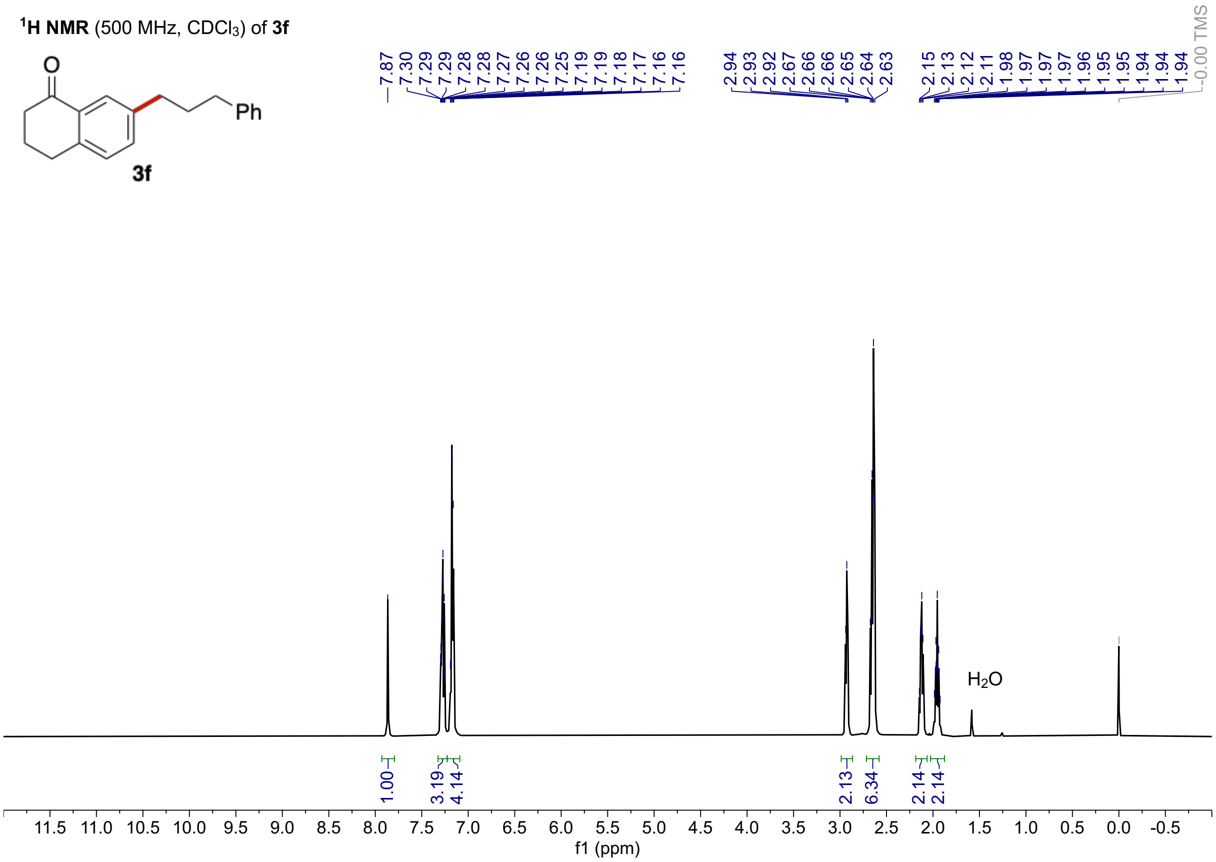

^{13}C NMR (500 MHz, $CDCl_3$) of **3f**

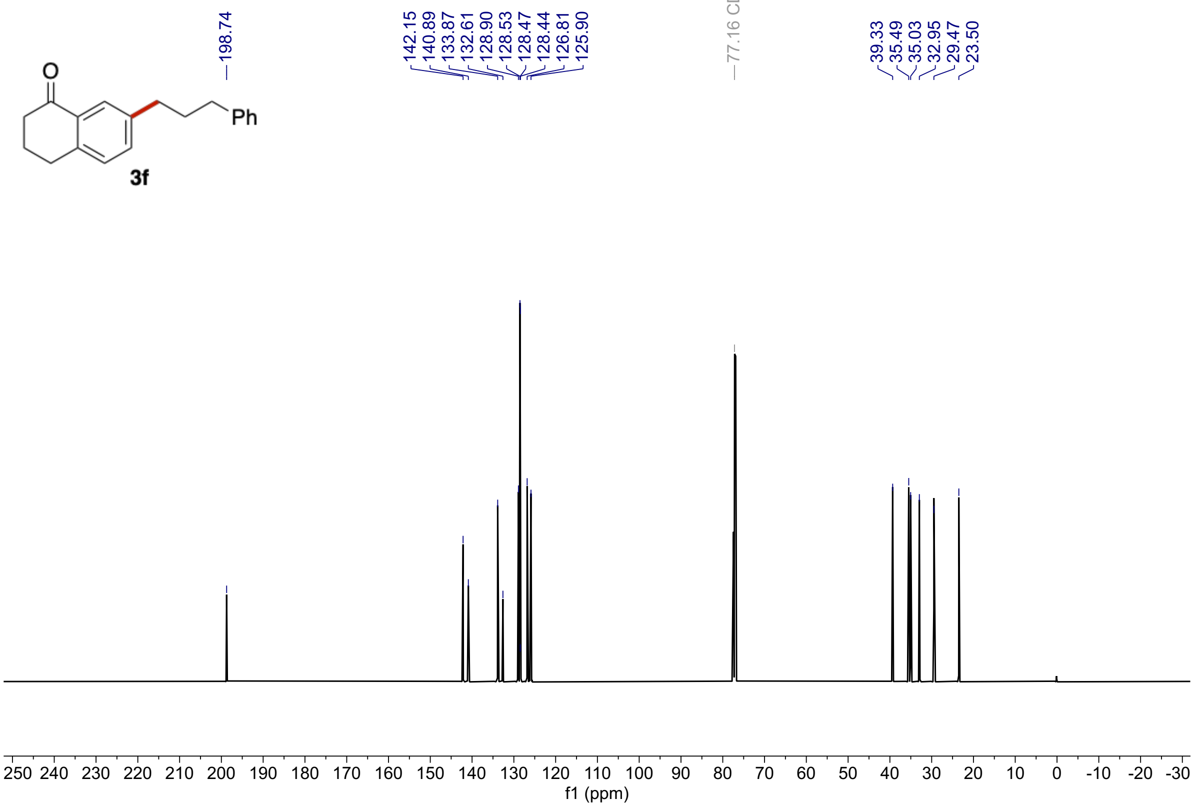

^{1}H NMR (500 MHz, $CDCl_3$) of **3g**

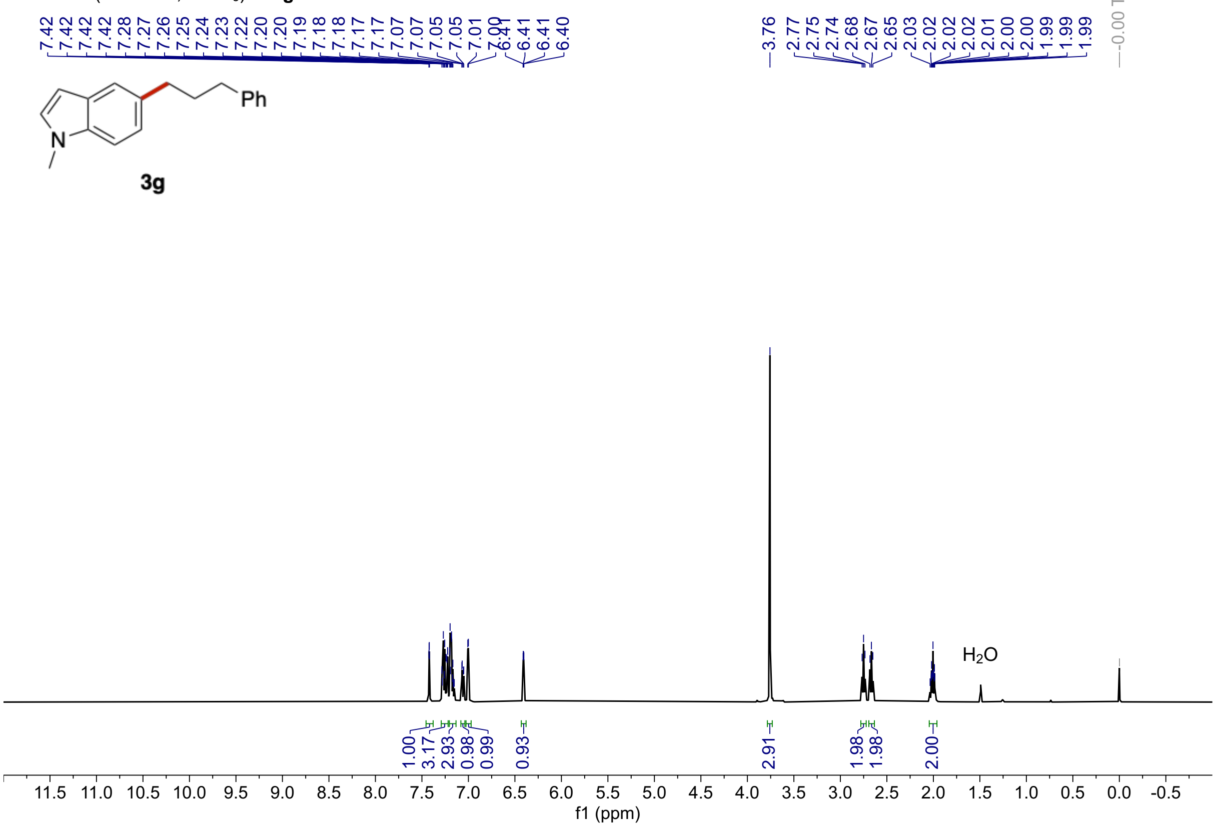

^{13}C NMR (500 MHz, $CDCl_3$) of **3g**

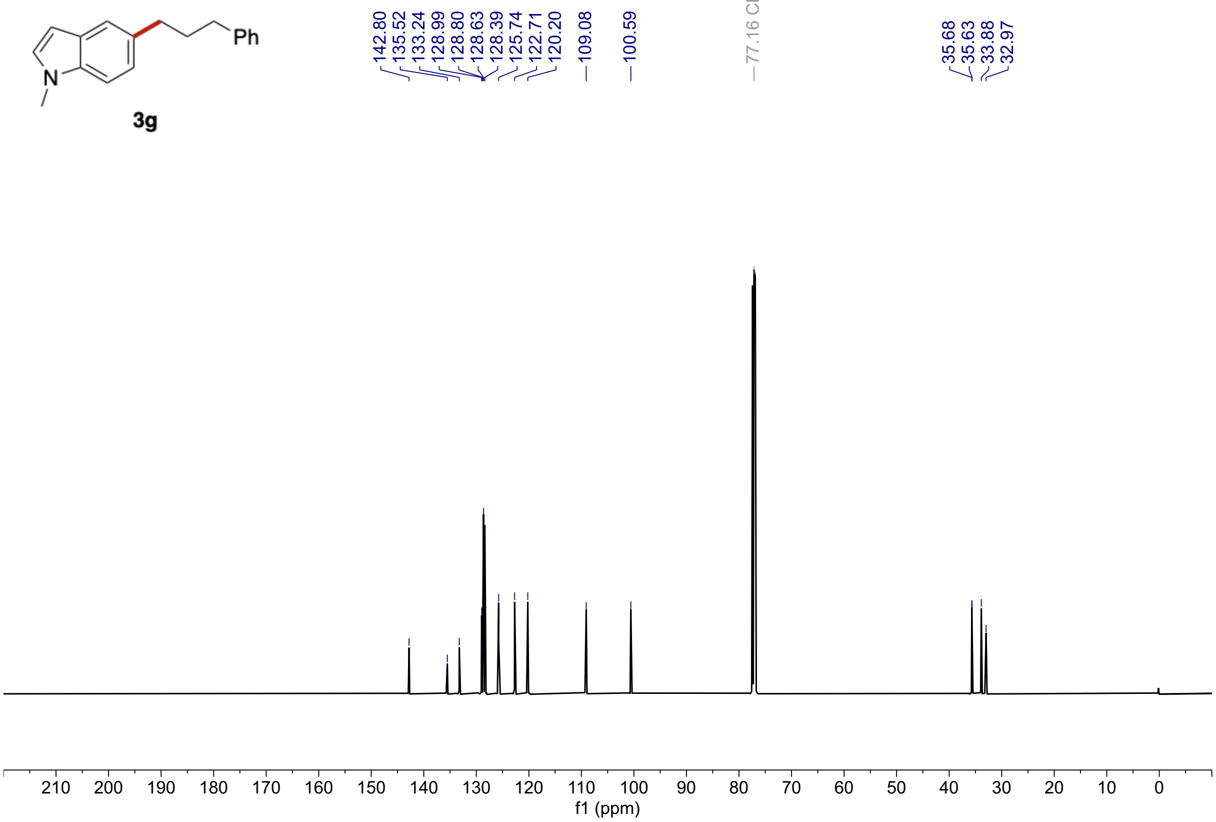

^{1}**H NMR** (500 MHz, $CDCl_3$) of **3h**

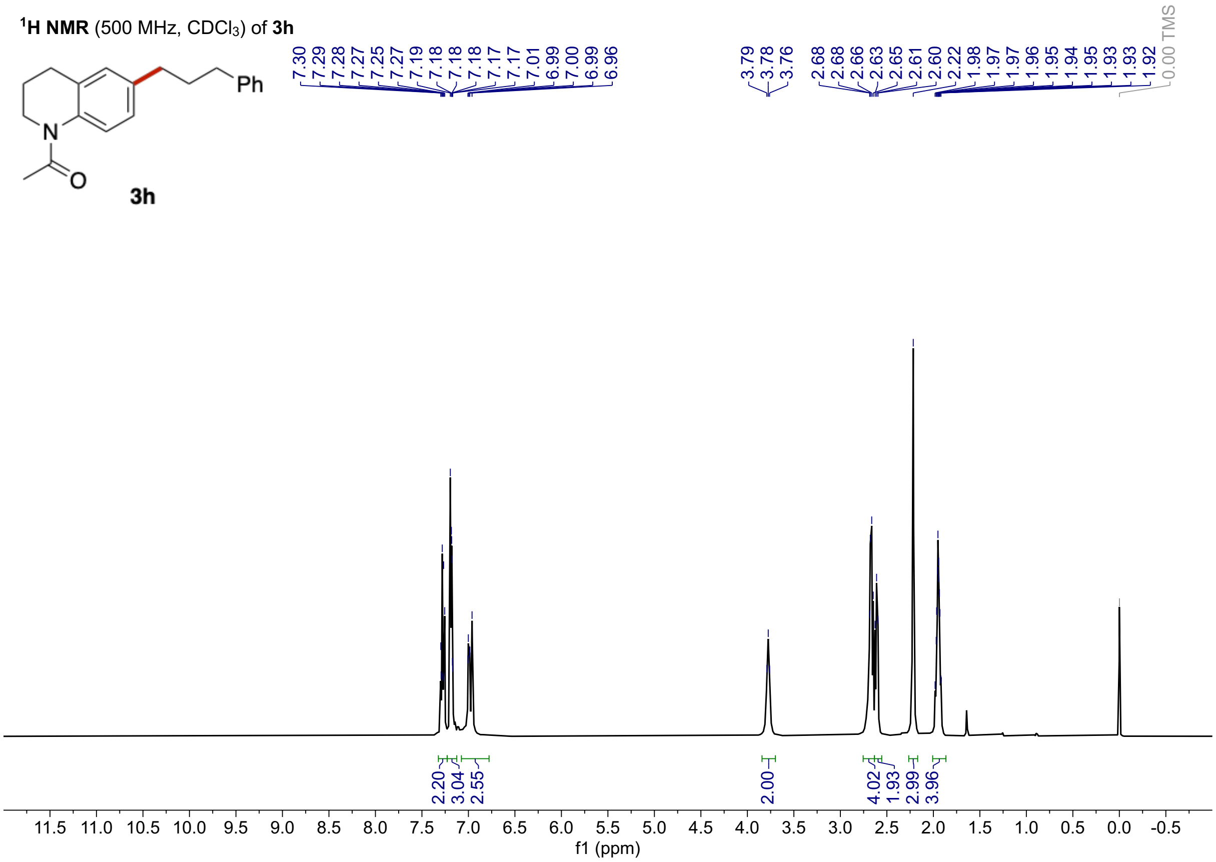

^{13}**C NMR** (500 MHz, $CDCl_3$) of **3h**

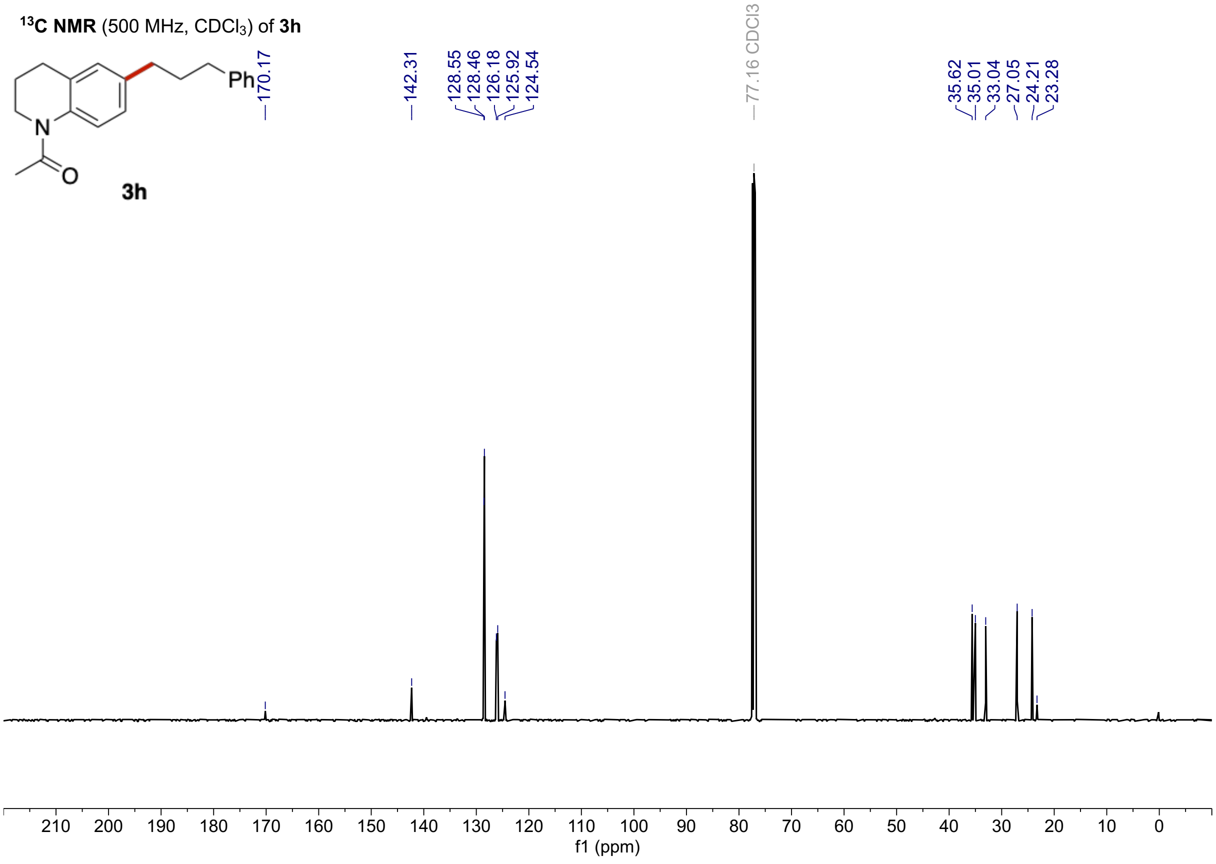

^{13}C quantitative NMR (500 MHz, $CDCl_3$) of **3h**

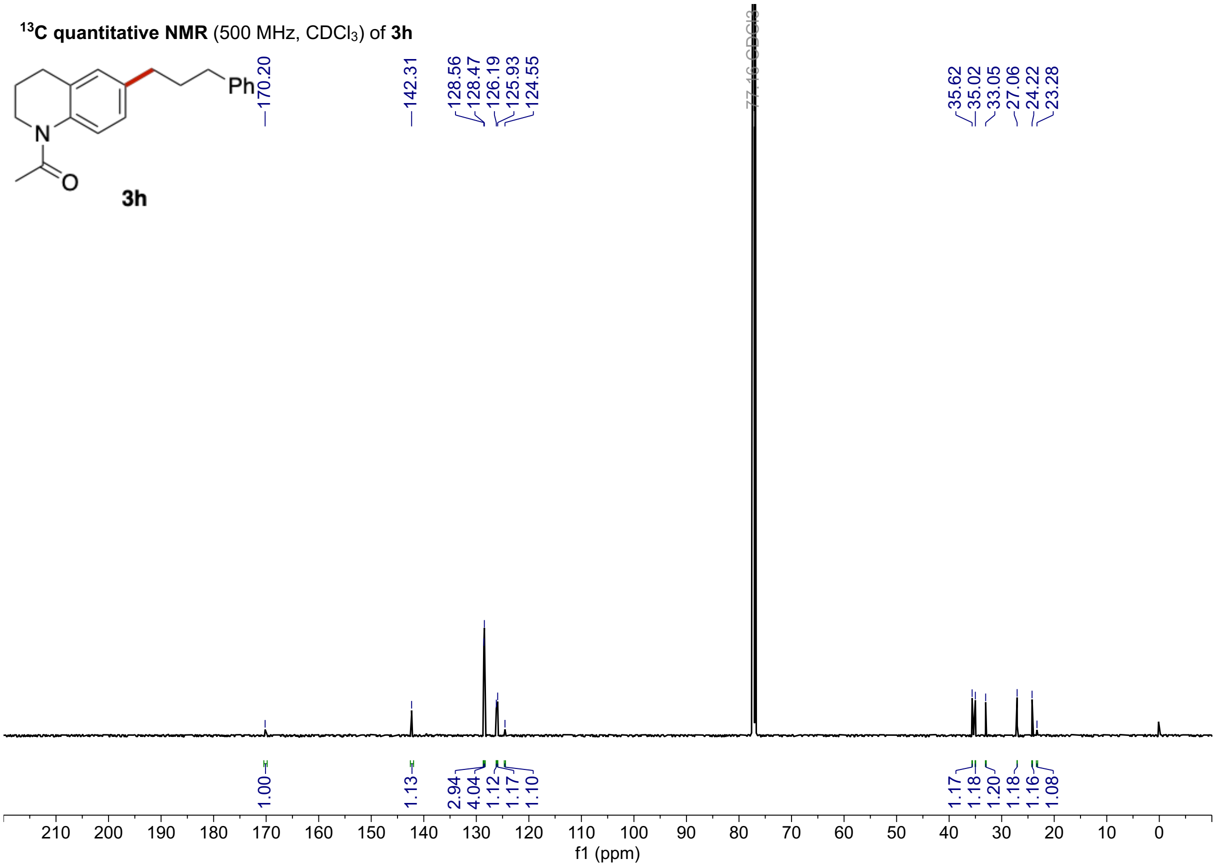

^{1}H NMR (500 MHz, $CDCl_3$) of **3i**

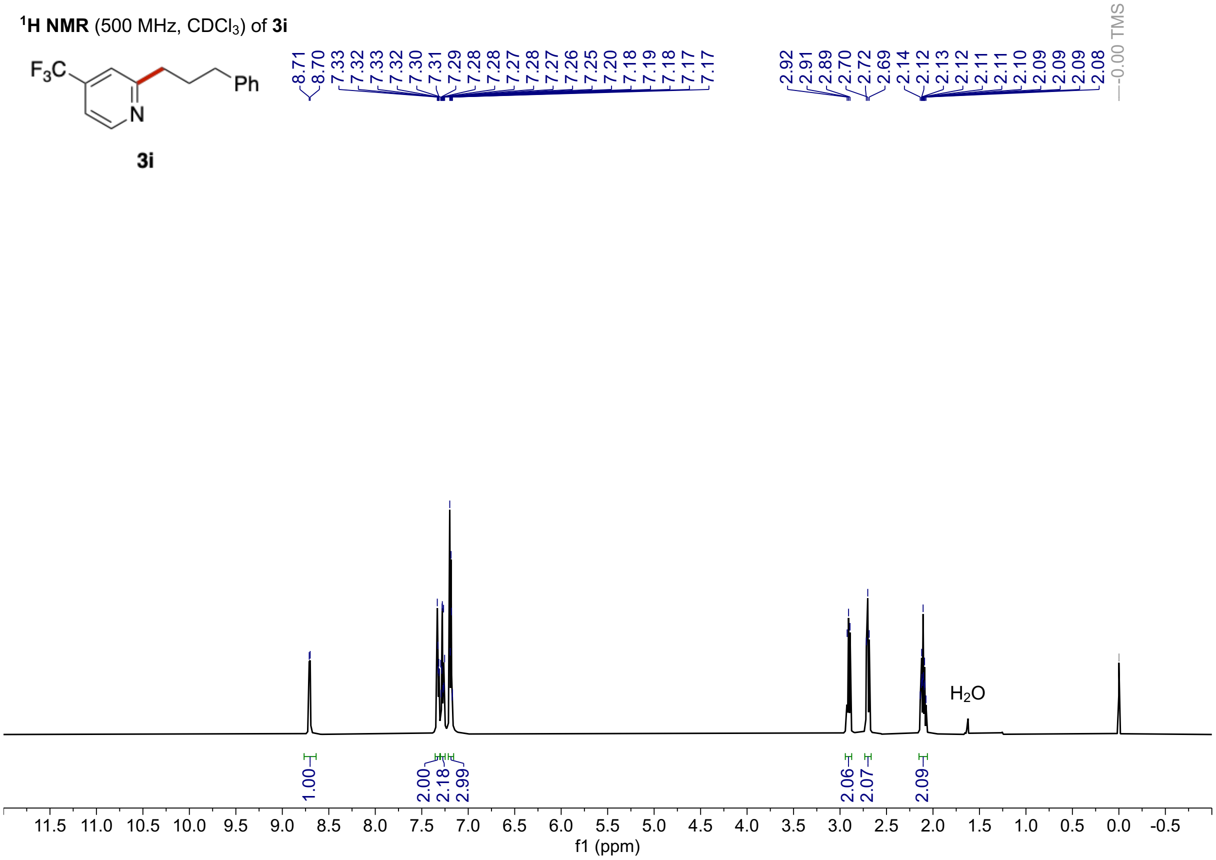

^{13}C NMR (500 MHz, $CDCl_3$) of **3i**

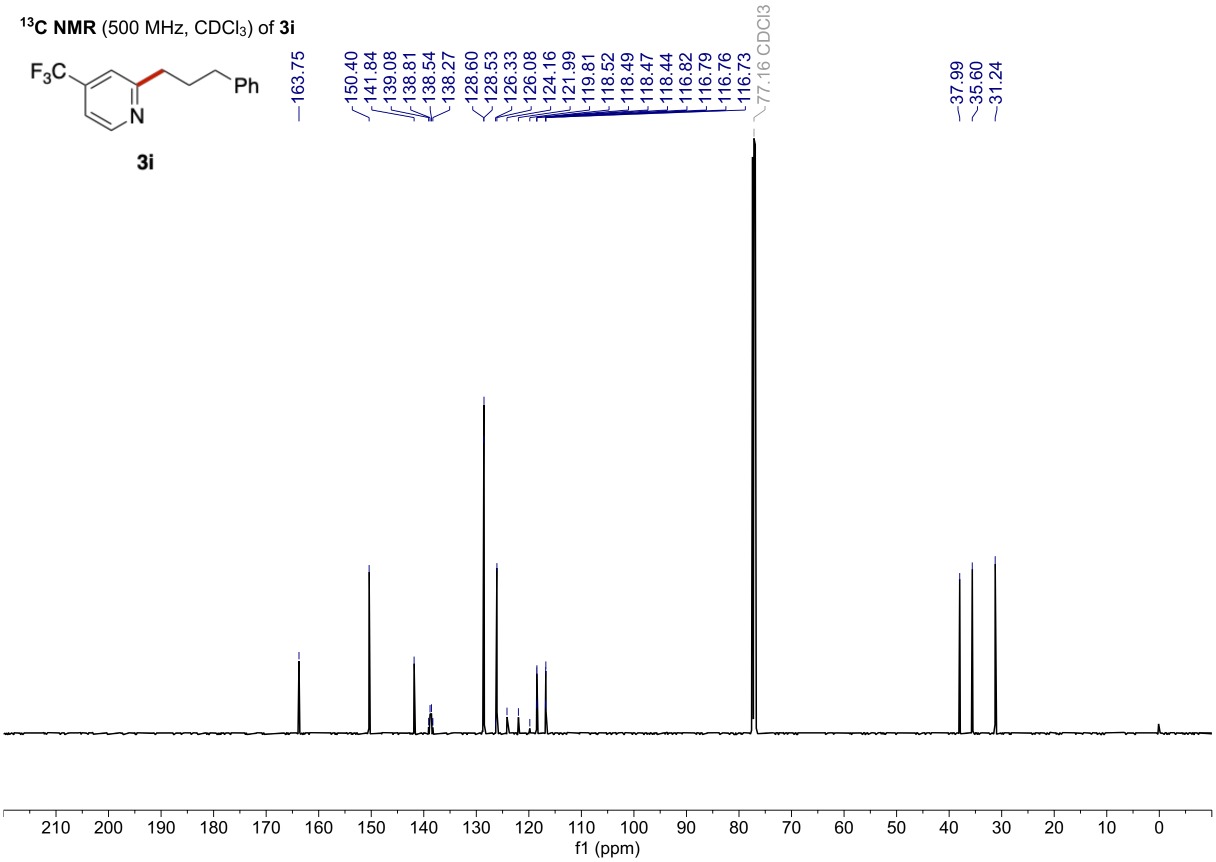

^{19}F NMR (500 MHz, $CDCl_3$) of **3i**

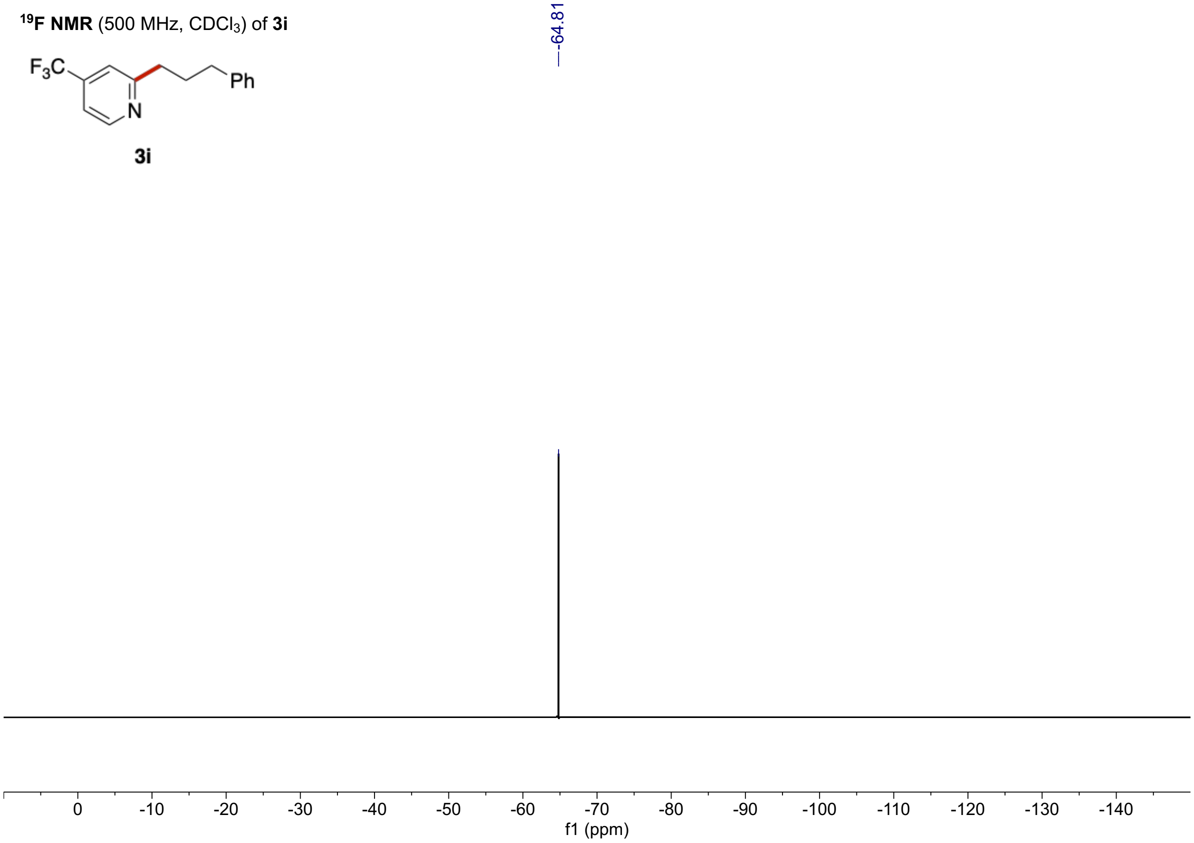

^{1}H NMR (500 MHz, $CDCl_3$) of **3j**

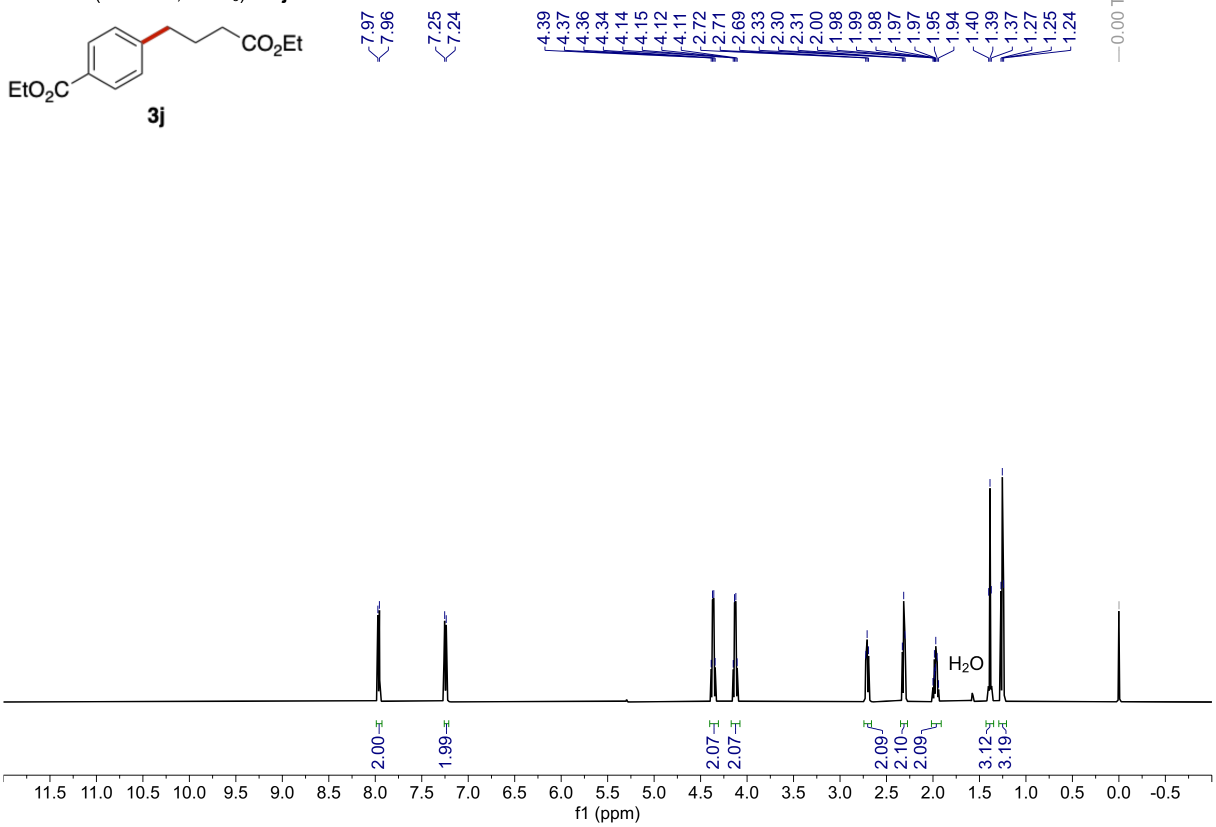

^{13}C NMR (500 MHz, $CDCl_3$) of **3j**

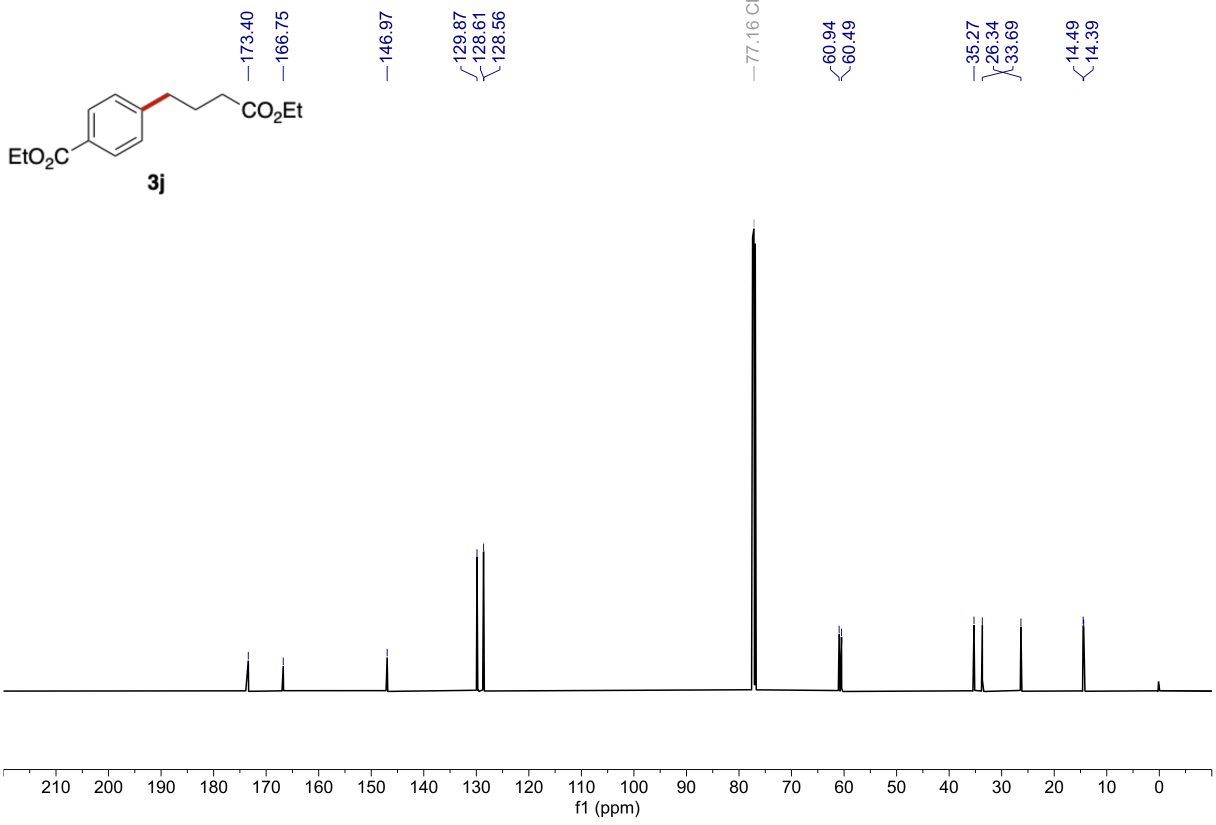

^{1}**H NMR** (500 MHz, $CDCl_3$) of **3k**

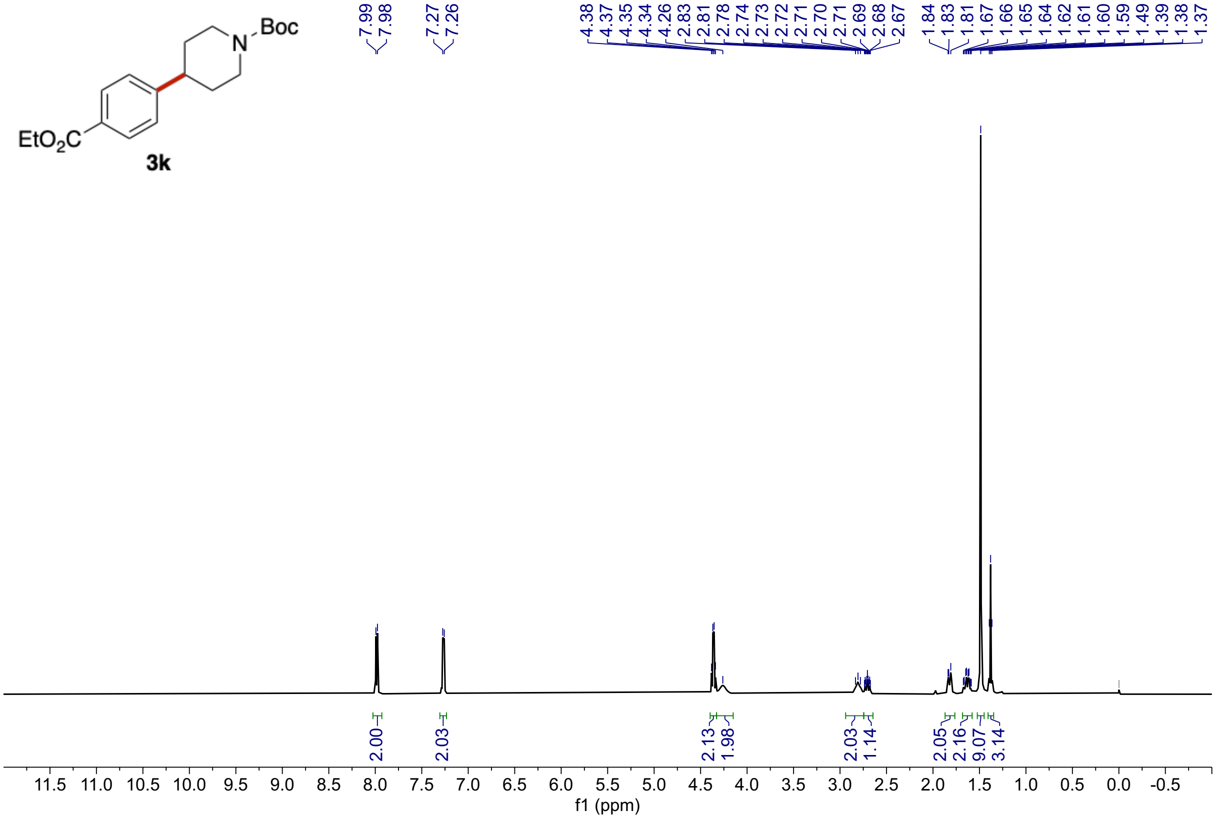

^{13}**C NMR** (500 MHz, $CDCl_3$) of **3k**

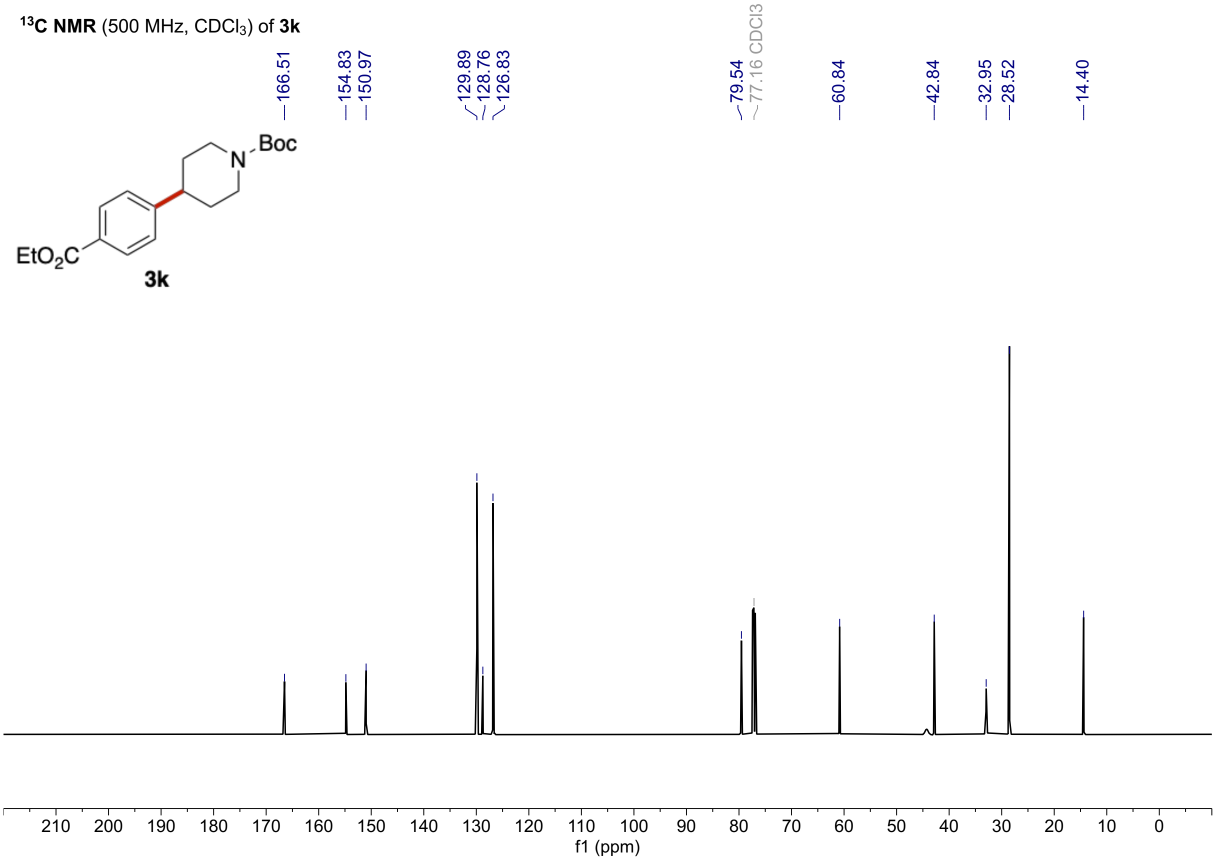

^{1}H NMR (500 MHz, $CDCl_3$) of **3l**

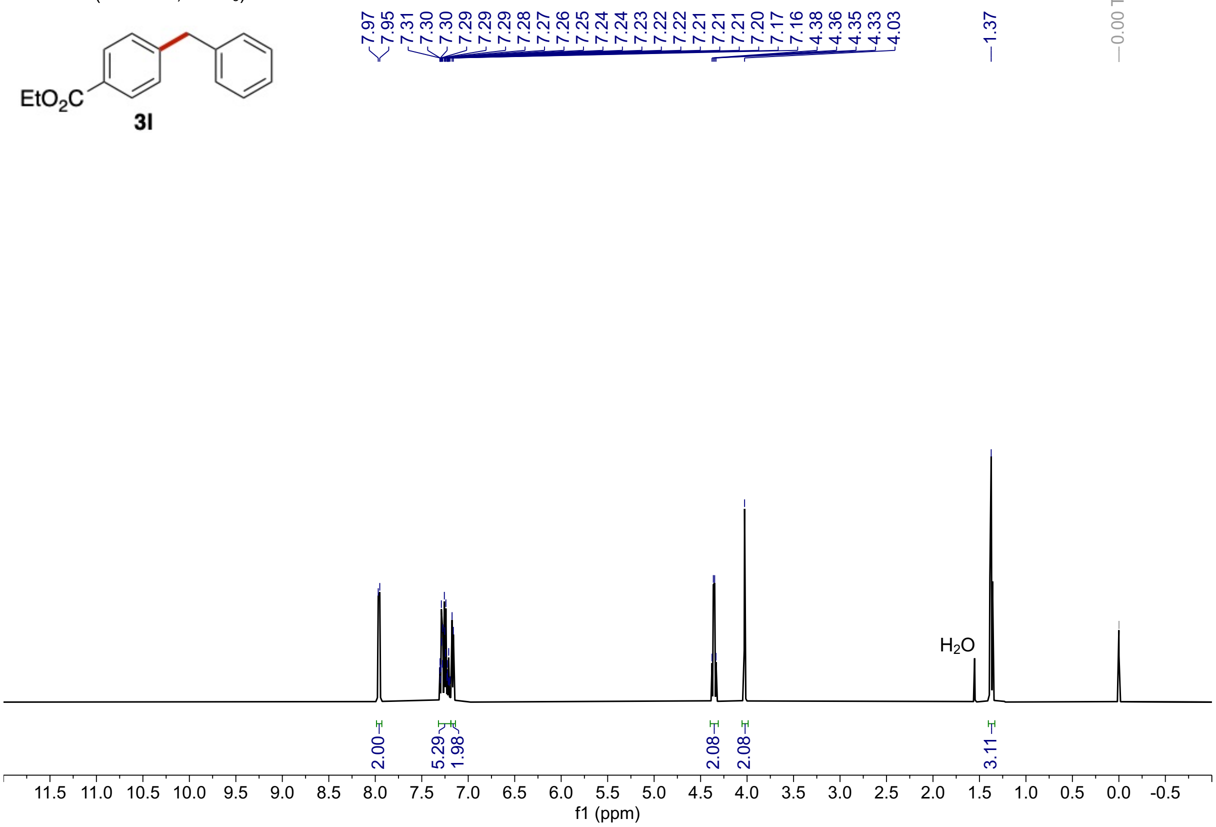

^{13}C NMR (500 MHz, $CDCl_3$) of **3l**

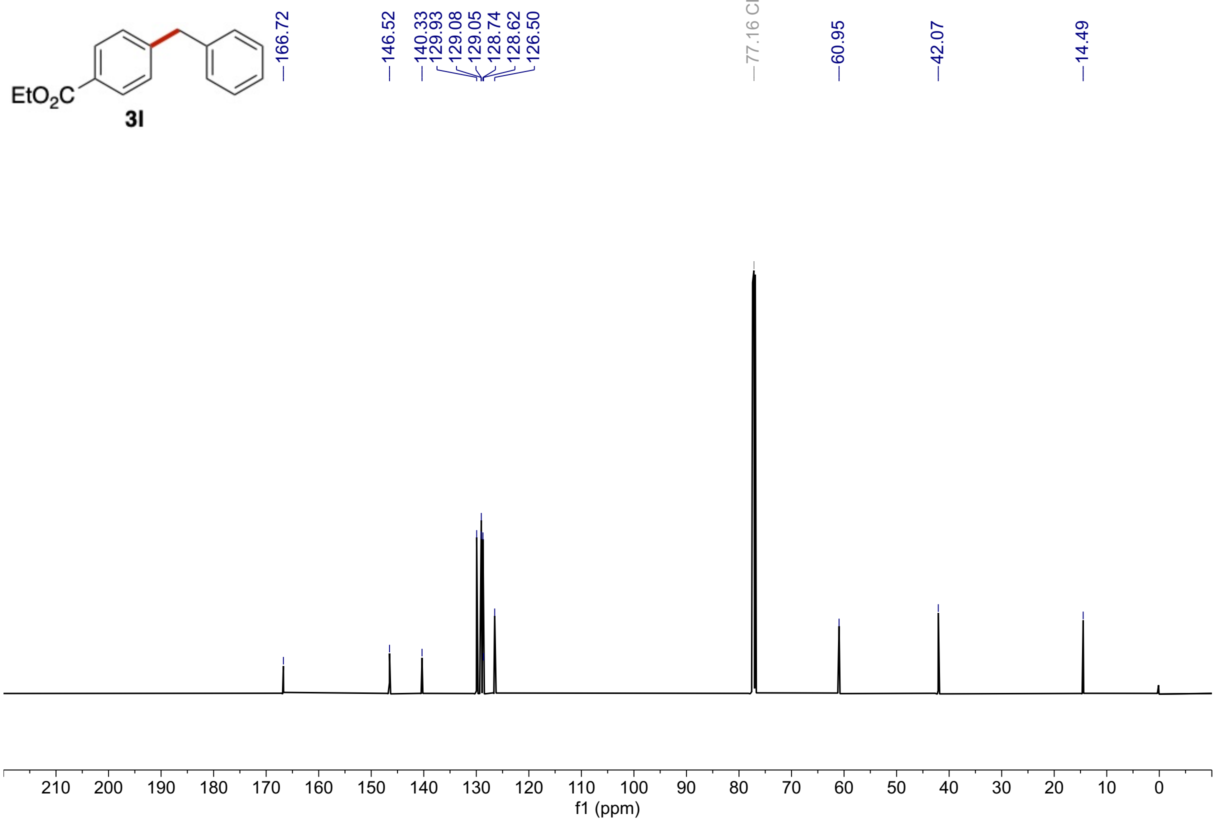

Supplement: Supplementary file 1 [file ja5c10599_si_001.pdf]
